# Wavelength-resolved heterodimer [2 + 2] photocycloadditions for reversible surface grafting

**DOI:** 10.1039/d5sc07978k

**Published:** 2026-01-27

**Authors:** Dushani Kanchana, Lauren Geurds, Aaron Micallef, Bryan Tuten, Kai Mundsinger, Christopher Barner-Kowollik

**Affiliations:** a School of Chemistry and Physics, Centre for Materials Science, Queensland University of Technology (QUT) 2 George Street Brisbane QLD 4000 Australia k.mundsinger@qut.edu.au christopher.barnerkowollik@qut.edu.au; b Central Analytical Research Facility, Queensland University of Technology (QUT) 2 George Street Brisbane QLD 4000 Australia; c Department of Chemistry and Biochemistry, The University of Texas at Tyler 3900 University Blvd. Tyler TX 75799 USA; d Institute of Functional Interfaces (IFG), Karlsruhe Institute of Technology (KIT) Herrmann-von-Helmholtz-Platz 1 76344 Eggenstein-Leopoldshafen Germany christopher.barner-kowollik@kit.edu

## Abstract

We report the first wavelength-dependent quantum yields of a [2 + 2] photocycloaddition generating the heterodimers of 7-hydroxycoumarin (7HCou) and styrene *via* a photochemical action plot. The wavelength-dependent heterodimer quantum yields are quantified at a constant number of photons at each wavelength between 310 and 370 nm. The resulting wavelength-dependent quantum yields demonstrate that the heterodimer is most efficiently generated at 345 nm, red-shifted by close to 25 nm compared to the absorption maximum of 7HCou at 320 nm. We subsequently translate these findings to photochemical surface functionalization by exploiting heterodimer formation between a surface bound coumarin derivative and *para*-styrene perfluoroalkyl ether (StyPFA) on surfaces under 345 nm irradiation to reversibly modulate surface hydrophobicity. The reversibility of the surface heterodimerization is demonstrated by removing StyPFA under UVC irradiation, and re-functionalization on the same surface. Functional heterodimer formation and the reversibility of the reaction on surface are followed *via* surface-sensitive X-ray photoelectron spectroscopy (XPS) and contact angle measurements. We subsequently apply our photochemical surface functionalization strategy to a dual cure photoresin based on a polyurethane-acrylate interpenetrating network, without deterioration of its mechanical properties, thereby confirming the feasibility of a photocycloaddition-based functionalization strategy for photoresins.

## Abstract 2

We report the first wavelength-dependent quantum yields of a [2 + 2] photocycloaddition generating the heterodimers of 7-hydroxycoumarin (7HCou) and styrene *via* a photochemical action plot.

## Graphical abstract

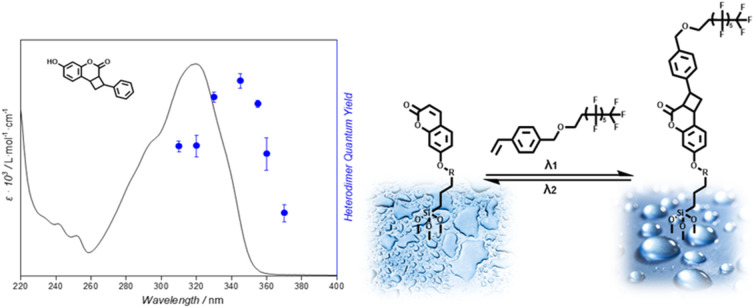

## Introduction

Photochemically induced cycloadditions offer a sophisticated and versatile platform for the reversible formation and cleavage of covalent bonds, allowing precise control through the use of distinct wavelengths of light.^1–3^ Among photocycloaddition reactions, the [2 + 2] cycloaddition is a powerful synthetic tool, resulting in the formation of a cyclobutane ring through the concerted conversion of two alkene double bonds into four single bonds upon photoexcitation.^4^ The photochemical [2 + 2] cycloaddition exhibits remarkable versatility, accommodating a broad range of substrates, from small molecules^5–7^ to complex (macro)molecular architectures.^8^

In soft matter materials science, photocycloadditions play a critical role in surface functionalization, enabling the design of responsive surfaces with reversible binding capabilities.^9^ They are particularly useful for applications such as self-healing materials,^10–12^ stimuli-responsive^13,14^ and functional coatings.^12,15^ Further, in polymer chemistry, [2 + 2] cycloadditions offer a powerful tool to crosslink polymer networks,^16^ facilitating the synthesis of tuneable materials with enhanced properties,^8,16^ and dynamic reversibility.^17–19^

The first known [2 + 2] photocycloaddition reaction was reported by Ciamician and Silber in 1908, involving the formation of carvone-camphor when exposing (+)-carvone to sunlight.^20^ Since then, extensive studies have been conducted on [2 + 2] cycloadditions, expanding our understanding of its reaction mechanism and scope, while exploring its applications in various fields. Generally, [2 + 2] cycloadditions do not occur thermally because they are symmetry forbidden, however upon light excitation an electron will transition from the HOMO to the LUMO, generating a new HOMO with a different orbital symmetry. The excited state HOMO matches the orbital symmetry of the ground state LUMO allowing the cycloaddition to proceed. Due to the short excited state lifetime, in the range of 10^−12^ s, the reaction requires both reaction partners to be in alignment already at the excitation event.^21^ Thus, the reaction is more likely to occur between identical molecules where molecular interactions such as π stacking, and hydrogen bonding are more likely to lead to a suitable alignment.^22,23^ Therefore, [2 + 2] cycloadditions are predominantly explored as homodimerizations.

Heterodimerizations are challenging not only due to the required orbital alignment, but factors related to reactivity, energy transfer and molecular geometry.^18,24,25^ Moreover, steric hindrance due to the differences in the size or shape of the heterodimer partners, and energy transfer processes such as non-radiative decay can further hinder the formation of heterodimers.

However, despite its inherent challenges, heterodimerization *via* [2 + 2] cycloaddition is a highly promising reaction due to the distinct properties and applications it offers, many of which are unattainable through homodimerization. Heterodimerization allows for the formation of asymmetrical cyclobutane derivatives. This structural diversity is critical for applications such as drug delivery systems, where unique structural motifs are required.^2,3^ Further, heterodimerization allows for the integration of two distinct functionalized alkenes, broadening the chemical and physical properties of the resulting product and leading to hybrid characteristics derived from each component. These features enable precise control over optical, electronic and mechanical properties, which is especially beneficial for photoresponsive materials and organic electronics.

Finally, compared to conventional photochemical protocols, (hetero)dimerization offers reversibility, and the careful choice of chromophore allows reactivity in only the desired wavelength regime. Heterodimerization specifically offers the advantage of a lower dimer extinction coefficient compared to homodimers when a suitable, smaller reaction partner is used. Small molecular reaction partners concomitantly offer easy availability in form of readily available commercial compounds, for example vinyl functionalized monomers. Many of these are available with functional handles allowing facile modification with desired functionalities.

To promote hetero- and reduce the homodimerization, various strategies can be utilized such as implementing pre-organization methods including covalent anchoring,^26^ adjusting concentrations,^27^ fine-tuning catalysts and sensitizers,^28^ and using selective irradiation.^29,30^

Photochemical action plots^31^ – introduced by our team – have become a key tool for understanding the wavelength-dependent reactivity of photochemical processes, including [2 + 2] cycloaddition reactions.^19,32,33^ Photochemical action plots demonstrate how different wavelengths lead to different photochemical reactivity, often revealing discrepancies between absorption maxima and wavelength dependent quantum yields for a given photochemical reaction trajectory.^31,32,34,35^

Over the last few years, a range of photochemical action plots has been recorded for homodimer forming [2 + 2] cycloadditions. One such example is a study investigating the green light-induced [2 + 2] cycloaddition of a halochromic system based on a styrylquinoxaline moiety.^33^ The pH-dependence of the photoreactivity was mapped *via* constant photon action plots and indicates that the choice of solvent strongly impacts the system's photoreactivity. Further, our team investigated visible light [2 + 2] cycloadditions for reversible polymer ligation of styrylpyrene, analysing the wavelength dependent photon efficiency.^19^ We demonstrated efficient reactivities for visible light induced cycloaddition and UV-triggered cycloreversion and further applied this system for polymer ligation. Wavelength resolved action plots identified the optimal wavelengths for dimerization (*λ* = 435 nm) and dissociation (*λ* = 330 nm), whereas the maximum absorption occurred at 375 nm. Moreover, we introduced a photoreactive group, acrylamidylpyrene conjugated to a linear PEG linker (AP-PEG), which can undergo a [2 + 2] cycloaddition reaction *via* visible light irradiation at unexpected wavelengths with the wavelength-dependent reactivity revealed through a photochemical action plot.^32^ The action plot revealed that the reactivity of the system is highest at 430 nm, whereas the absorption maximum is close to 370 nm. Critically, reactivity of AP-PEG was observed up to 490 nm – a wavelength where the absorption spectrum only displays weak absorption (*ε* < 500 L mol^−1^ cm^−1^) – making it one of the most red-shifted photocycloadditions reported to date.

Since then, the mismatch between the absorption maximum and photochemical reactivity was observed across other photochemical reactions – for example in photochemical ring contractions^36^ – and we recently provided an initial experimentally supported theory for this discrepancy, exemplified by the [2 + 2] photocycloaddition of a pyrene-chalcone.^37^ A solution of molecules will contain distinct microenvironments with varying solvent–solute interactions that give rise to different ground- and excited-state energies, due to orientation of the molecules' dipole moments. Under such conditions, longer-wavelength (lower-energy) irradiation can selectively excite molecules in the lowest-energy microenvironment. This phenomenon is photophysically underpinned by the red-edge effect (REE), wherein a subset of chromophores in a bulk solution can be selectively excited by longer wavelengths of light.^38^ The REE can be experimentally validated by fluorescence spectroscopy.^39,40^ Both steady-state and time-resolved fluorescence spectroscopy confirm the observed selectivity, revealing significant red-edge effects in fluorescence spectra.^41,42^ Thus, the differences in solvent–solute interactions in both ground and excited states – together with variations in excited-state lifetimes – substantially influence the extent of photochemical reactivity and account for the observed mismatch between absorption and wavelength-dependent quantum yields on the red-side of the absorption spectrum. Therefore, understanding excited state lifetimes and their wavelength dependency is beneficial for most photochemical systems, including bimolecular reactions such as [2 + 2] cycloadditions.^37^

**Scheme 1:**
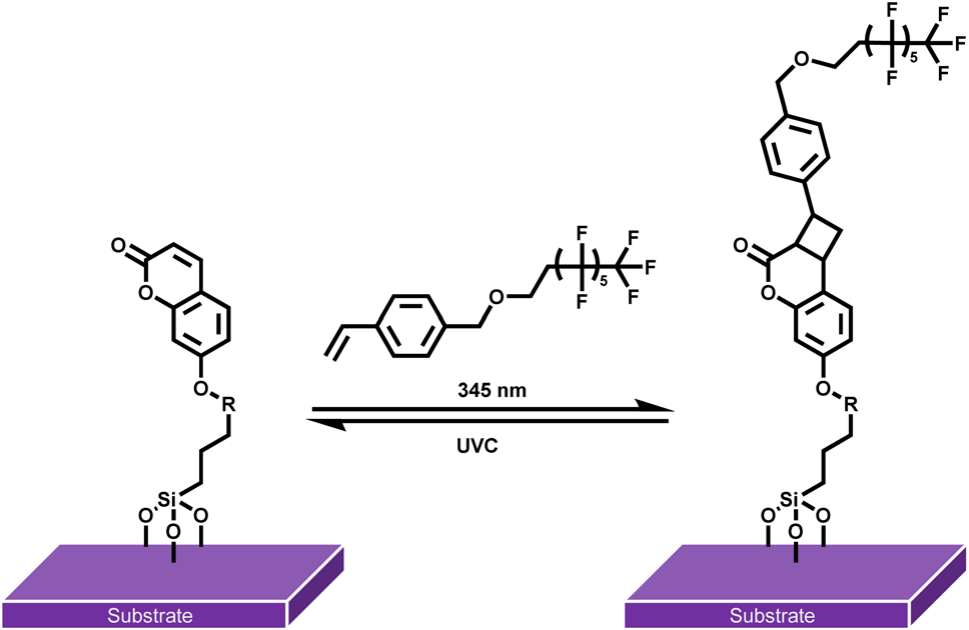
Reversible surface grafting of *para*-styrene perfluoroalkyl ether (StyPFA) onto SiO_2_ substrates *via* [2 + 2] heterodimerization with surface bound coumarin.

Although numerous photochemical action plots have been reported for [2 + 2] homodimerization, heterodimerizations remain unexplored in a wavelength resolved fashion. Herein, we introduce the first photochemical action plot for a heterodimer [2 + 2] cycloaddition, providing key insights into the wavelength-dependent quantum yields of heterodimer formation between 7-hydroxycoumarin (7HCou) and styrene. After identifying the wavelength dependent quantum yield for the heterodimer formation in solution – and thus its most effective wavelength – we extend our investigation to reversible surface grafting of a fluorinated moiety through heterodimerization on surfaces. Our approach thus integrates the careful measurement of wavelength-dependent quantum yields as a guiding tool for subsequent surface functionalization to impart hydrophobic functionality onto surfaces (Scheme 1).

## Results and discussion

### Heterodimerization [2 + 2] photochemical action plot of 7HCou and styrene

We initially investigate the heterodimer formation between 7HCou and styrene in acetonitrile (ACN) under *λ*_max_ = 325 nm irradiation (refer to SI Section 1.2.8 for the emission spectrum of the employed LED). Characterization of the irradiated sample by proton nuclear magnetic resonance (^1^H NMR) spectroscopy as well as liquid chromatography-mass spectrometry (LC-MS) confirmed the successful heterodimer formation. Interestingly, we did not observe any homodimer formation under the employed reaction conditions (1 : 142 7HCou : styrene ratio with an overall concentration of 30 mmol L^−1^). We selected styrene as reaction partner as we observed significantly higher conversion of styrene and 7HCou compared to acrylates and methacrylates in our preliminary experiments. This can likely be attributed to the structural similarity between styrene and 7HCou, which promotes π–π stacking and favourable alignment of the molecules.^43^

**Fig. 1:**
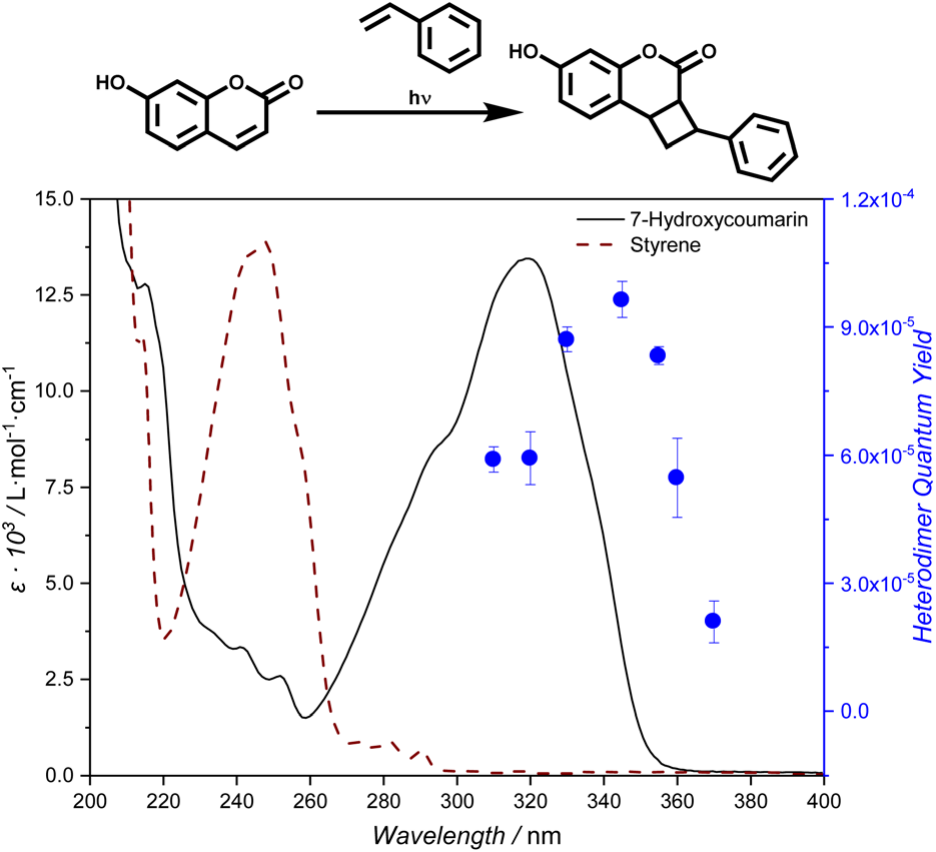
Top: Reaction scheme of the heterodimerization of 7HCou and styrene. Bottom: Heterodimerization action plot with reaction quantum yields at each wavelength overlaid with the molar extinction spectra of 7HCou and styrene.

Having confirmed the formation of heterodimer in solution, we subsequently examined the wavelength-dependent formation of the 7HCou-styrene heterodimer through [2 + 2] cycloaddition by recording a photochemical action plot in the wavelength range from 310 to 370 nm. The experimental methods for recording photochemical action plots are described in detail in Sections 3.1 and 3.2 of the SI. Each sample is irradiated with an identical number of photons of monochromatic light across the wavelength region of interest. We chose a 7HCou to styrene ratio of 1 : 10, while maintaining an overall concentration of 10 mmol L^−1^. Heterodimer formation was monitored by tracking the appearance of the cyclobutane resonances *via*^1^H-NMR spectroscopy. In particular, the resonances emerging in the 2.9 to 2.6 ppm range, corresponding to the cyclobutane protons derived from styrene, were used to determine the heterodimer yield (refer to the SI for details). We further confirmed heterodimer formation at each wavelength *via* LC-MS. Interestingly – as clearly demonstrated in the LC trace (SI Section 2.3) – upon irradiation, not only was the heterodimer (at RT 8.22–8.83 min) formed, but a small percentage (<10%) homodimer was also observed under the employed reaction conditions. We could not identify a wavelength dependency of the homo- to heterodimer ratio. However, we are currently conducting a detailed study comprising a range of heterodimers. The wavelength resolved conversion was converted into the heterodimerization quantum yield using the extinction coefficient at each wavelength (refer to SI Section 3.2.2 for a detailed description) and overlayed with the molar absorbance spectrum of 7HCou. The resulting photochemical action plot (Fig. 1) shows the maximum quantum yield at 345 nm, red shifted close to 25 nm from the absorbance maximum, *λ*_max_ = 320 nm. The recorded quantum yields are based on the conversion to all (head-to-head, head-to-tail, syn, anti) heterodimer isomers. The observed red-shift of the quantum yields compared to molar absorptivity is likely caused by the same REE microenvironment effect discussed above.^37^ We note that absorption occurs almost exclusively *via* 7HCou, while styrene displays negligible absorbance in this region (refer to Fig. 1). We subsequently investigated the heterodimerization of a coumarin functionalized surface with a fluorinated styrene derivative.

### Coumarin-StyPFA heterodimer formation on Si substrates

To highlight the versatility and applicability of the photochemical heterodimer functionalization system, we regulated the surface hydrophobicity of Si-wafers *via* the attachment of a perfluorinated alkyl moiety. Specifically, *para*-styrene perfluoroalkyl ether (StyPFA) was synthesized (refer to the SI Section 2.1.6 for the detailed procedure). We probed the 7HCou-StyPFA heterodimer formation first in solution *via* irradiation of a solution of 7HCou and StyPFA (1 eq. 7HCou to 20 eq. StyPFA, combined concentration 100 mmol L^−1^) in acetonitrile (refer to SI Section 2.2.2). The formation of the heterodimer was confirmed *via*^1^H-NMR spectroscopy and LC-MS.

**Fig. 2:**
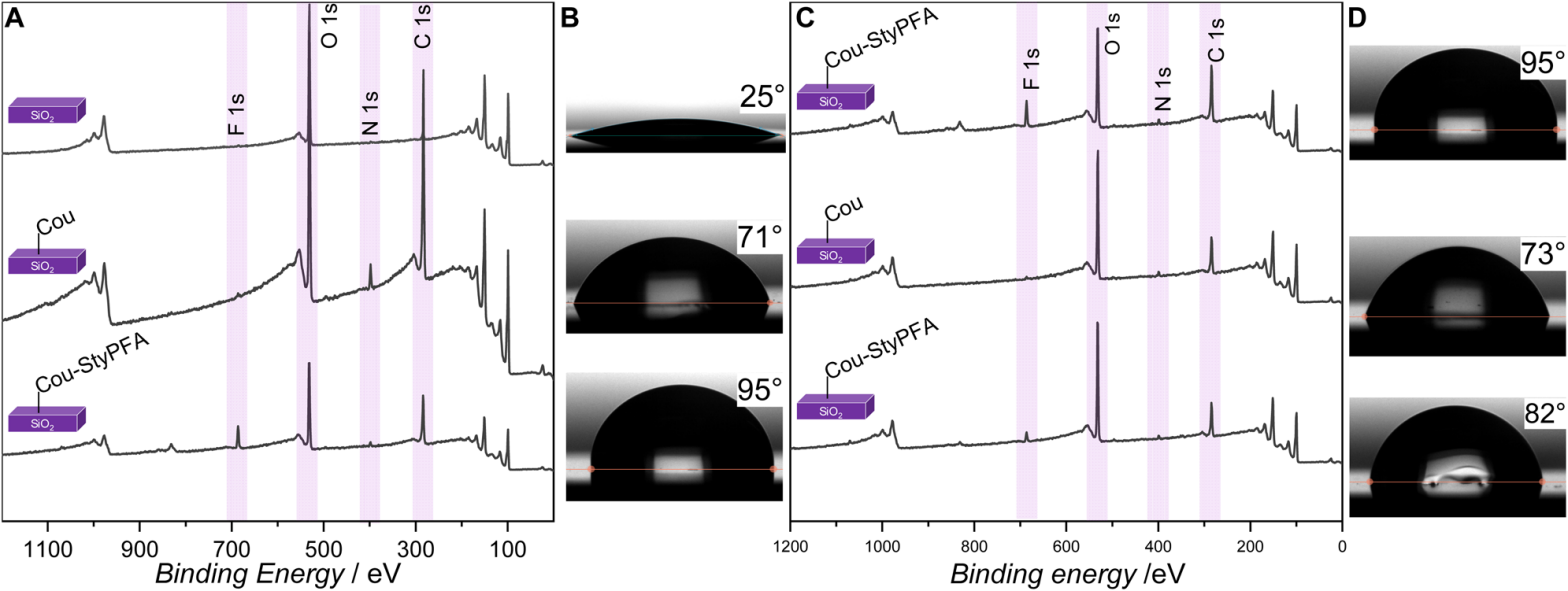
(A) XPS wide scan spectra of coumarin-StyPFA heterodimer formation on SiO_2_ substrate. Initial surface, coumarin-silane functionalized surface and StyPFA functionalized surface respectively from top to bottom. (B) Contact angle measurements for the initial surface, coumarin silane functionalized surface and StyPFA functionalized surface respectively from top to bottom. (C) XPS wide scan spectra after StyPFA functionalization on the surface, cycloreversion on same surface and re-functionalization on same surface, respectively from top to bottom. (D) Contact angle measurements on the surface after first StyPFA functionalization, after cycloreversion on the surface and after re-functionalization on the same surface respectively from top to bottom.

We subsequently functionalized 7HCou with aminopropyl triethoxysilane to obtain a silane functionalized coumarin, enabling us to tether the coumarin moiety onto a Si substrate and trace the heterodimer formation on the surface *via* X-ray photoelectron spectroscopy (XPS). The silane-functionalized coumarin was synthesized in four-steps. Initially, ethyl 5-bromovalerate was obtained by esterification of 5-bromovaleryl chloride with ethanol, followed by esterification with 7HCou and subsequent hydrolysis of the resulting coumarin ethyl valerate to yield coumarin ethyl valeric acid. Finally, EDC coupling with aminopropyl triethoxysilane afforded the silane-functionalized coumarin. The synthesized compounds were characterized *via*^1^H-NMR spectroscopy and LC-MS (for the detailed procedure and experimental data refer to SI Section 2.1). Subsequently, we functionalized SiO_2_ surfaces with coumarin *via* hydrolysis of the silyl ether in dichloromethane (DCM) (detailed procedure in SI Section 2.4.1). Successful surface functionalization was confirmed by XPS and contact angle measurements (Fig. 2A and B).

The XPS spectra (Fig. 2A) confirm the presence of the coumarin-silane compound on Si surfaces, indicating 66% carbon, 29% oxygen and 4% nitrogen – a C : O : N ratio of approximately 16 : 7 : 1 – in close alignment with the expected values (65% C, 27% O, 4% N). In addition, water contact angle measurements show an increase of 46° from 25° for bare SiO_2_ to 71° for coumarin functionalized wafers (Fig. 2B), further confirming the successful surface functionalization.

We subsequently performed the heterodimerization of the surface bound coumarin and StyPFA *via* LED irradiation at 345 nm, where we observed the maximum quantum yield. While we acknowledge that surface-bound coumarin may exhibit subtle differences in photochemical properties compared to the solution phase – due to altered local polarity, molecular arrangement or restricted mobility – the selection of 345 nm ensures excitation in the high-efficiency region, maximizing the probability of heterodimer formation. This choice is further supported by previous studies showing that excitation at the red-edge of absorption bands can enhance selectivity and quantum yield in surface-immobilized photoreactions.^44^

Efficiency of the dimerization on the surface is potentially further enhanced due to a high local concentration of StyPFA on the surface. Highly fluorinated molecules can show interesting solution behaviour compared to their non-fluorinated analogues. A formed Cou-StyPFA dimer on the surface possibly facilitates functionalization in its surroundings due to fluorophilic interactions.

The formation of the coumarin-StyPFA heterodimer was confirmed by XPS (Fig. 2A, spectrum Cou-StyPFA). The XPS spectrum shows an F 1s peak at 685 eV with approx. 7% fluorine relative to other elements, compared to the theoretical maximum of 22% that corresponds to approximately 30% functionalization efficiency. Considering the relatively low heterodimerization quantum yields observed in solution, the surface functionalization is surprisingly efficient. Additionally, an increase in water contact angle by 24°, resulting in 95° (Fig. 2B), indicates an enhancement in surface hydrophobicity, underpinning the successful [2 + 2] heterodimer formation on the surface.

### Photoinduced cycloreversion

To investigate the reversibility of the surface-bound heterodimer, functionalized Si wafers were exposed to UVC irradiation for two hours while immersed in acetonitrile (refer to SI Section 1.2.7 for the emission spectra and Section 2.4.3 for the detailed experimental procedure). Following the irradiation, the wafers were thoroughly rinsed with acetonitrile and acetone, dried under a nitrogen flow, and subsequently analysed by XPS. As shown in Fig. 2C SiO_2_-Cou, the decrease in the F 1s peak intensity confirms the cleavage of the heterodimer and removal of the fluorinated moiety from the surface under UVC irradiation. Approximately 1% fluorine remained on the surface, corresponding to 85% removal of fluorine from the surface.

The above result was further supported by contact angle measurements (Fig. 2D), which showed a decrease from 95° to 73°, consistent with the reduction in surface hydrophobicity following the loss of fluorination. To examine the recyclability of the system, the same wafer was subjected to another functionalization cycle. The corresponding XPS data (Fig. 2C) clearly show the modulation of the surface fluorine content, with alternating decreases and increases of the fluorine signal during the cleavage and re-functionalization steps.

These observations were further validated by contact angle measurements (Fig. 2D), where values decreased from 95° to 73° after cleavage, and increased to 82° (Fig. 2D), upon re-functionalization. A slight loss of efficiency was observed over repeated cycles, most likely due to photodamage.

Nevertheless, these results demonstrate that surface functionalization *via* hetero [2 + 2] cycloaddition is reversible and repeatable, highlighting the potential of such systems for practical applications in surface coatings.

### Cou-StyPFA heterodimerization within a poly(urethane)-acrylate resin

To further highlight the potential applications of the surface-chemistry tunability by heterodimerization, we explored the incorporation of functionalized coumarin into a dual-cure resin. Dual-cure systems represent one of the versatile routes to generate interpenetrating networks (IPNs). IPNs are polymeric materials composed of two or more crosslinked networks that are physically entangled but not covalently bonded to each other. The merged architecture of the two polymer networks often results into enhanced mechanical strength, toughness, and chemical resistance compared to single-network systems. Dual-cure resins based on a photo-thermal curing process such as acrylate-polyurethane are widely used across various industries due to their processing efficiency and mechanical versatility.^45,46^ The rapidly photocured acrylate crosslinked network provides immediate structural integrity, whereas the secondary thermal cure, significantly improves the mechanical properties of the material. Since the primary industrial use of acrylate-polyurethane dual-cure systems lie in coatings, adhesives and 3D-printing photoresins, surface-specific functionalization through heterodimerization present an especially intriguing avenue to investigate.^47–49^

**Fig. 3:**
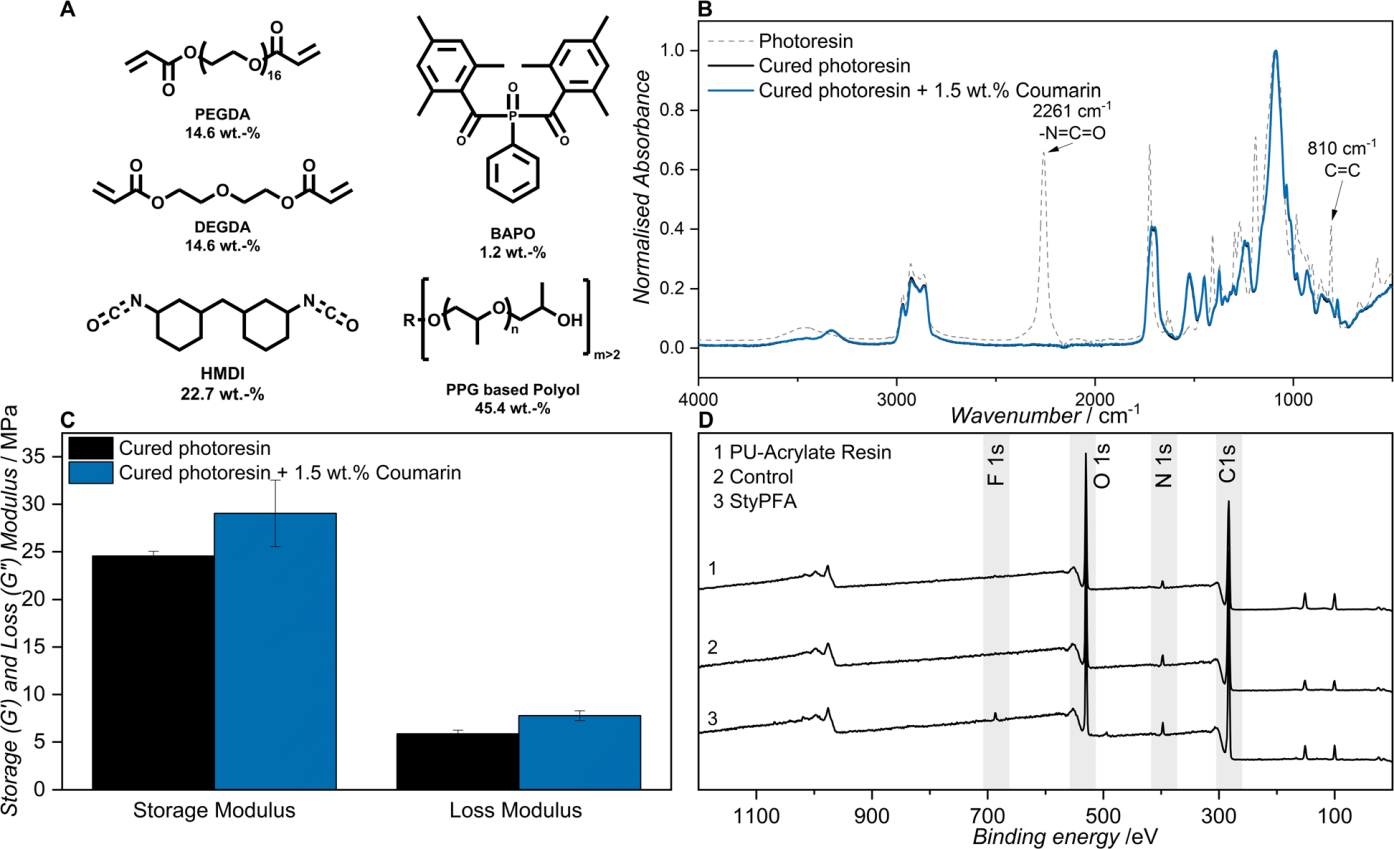
(A) Components of the polyurethane–acrylate dual cure resin (B) FT-IR spectra overlayed of uncured resin (dashed grey) and cured 30 wt% resin (black) and 30 wt% resin with 1.5 wt% coumarin (blue) in the range from 400 to 4000 cm^−1^. (C) Storage modulus (*G*′) and Loss modulus (*G*″) measured at a frequency of 1 Hz of cured 30 wt% resin (black) and 30 wt% resin with 1.5 wt% coumarin (blue). (D) Wide scan XPS spectra of 1 freshly cured resin, 2 after the StyPFA treatment (2 mmol L^−1^ in ACN) in the dark and 3 after the StyPFA treatment under light (2 mmol L^−1^ in ACN under UVA and the emission spectrum of UVA lamps can be found in SI Section 1.2.7).

Inspired by the work of DeSimone,^50^ we decided to assess the incorporation of our surface functionalization platform using a resin formulation for additive manufacturing. The resin consists of a commercial polyurethane, 4,4′-diisocyanato dicyclohexylmethane (HMDI) and a multifunctional polypropylene glycol, with an acrylate blend (polyethylene glycol diacrylate (PEGDA, M_n_ 250) with diethylene glycol diacrylate (DEGDA)), and phenylbis(2,4,6-trimethylbenzoyl) phosphineoxide (BAPO) as photoinitiator (refer to Fig. 3A).

We functionalized 7HCou with an acrylate–triethylene glycol (TEGA) chain to improve solubility and enable integration of the coumarin moiety into the acrylate network of the dual-cure resin. The acrylate-triethylene glycol functionalized coumarin (CouTEGA) was synthesized in two steps. Initially, TEGylated-coumarin (CouTEG) was obtained *via* alkylation of 7HCou with 2-[2-(2-chloroethoxy) ethoxy] ethanol, followed by esterification with acryloyl chloride resulting in the CouTEGA (for the detailed procedures and spectroscopic data refer to SI Section 2.1). Next, CouTEGA was incorporated into the resin at a 1.5 wt% concentration (relative) and cured *via* exposure to UVA light and elevated temperatures (refer to SI Section 2.6). A low concentration was deliberately employed to minimize any potential interference with the photocuring process, while preserving the inherent mechanical properties of the network.

Successful curing of the resin was confirmed by FT-IR spectroscopy, evaluating the disappearance of both the isocyanate functionality (2261 cm^−1^) and the double bond of the acrylate (810 cm^−1^) as depicted in Fig. 3B. The disappearance of both peaks indicates the formation of polyurethane and the conversion of the double bonds into a cross-linked acrylate polymer network. Given that the steric bulk of coumarin may affect network formation and consequently alter the mechanical properties of the cured material, dynamic mechanical analysis (DMA) was carried out. We found both the storage (*G′*) and the loss (*G′′*) moduli increased slightly with the addition of the coumarin functional moiety from 24.5 MPa to 29 MPa with regards to *G*′ and 5.9 MPa to 7.8 MPa for *G*′′ (refer to Fig. 3C).

We compared the extinction spectra of 7HCou to CouTEG and found them to almost identical (refer to Fig. S32) as the electronic structure of the molecule is virtually unchanged by the functionalization. We therefore postulate that the wavelength resolved reactivity of CouTEG and CouTEGA follows the same behavior as 7HCou. We subsequently investigated the functionalization of the cured coumarin containing resin with StyPFA. Prior to introducing the fluorine functionality, XPS analysis was performed on a freshly cured resin sample. As shown in Fig. 3D (PU-acrylate resin), peaks were observed at 285 eV, 569 eV, and 436 eV, corresponding to C 1s, O 1s, and N 1s, respectively. The fluorine functionality was subsequently introduced by immersing the resin in a StyPFA solution (2 mmol L^−1^ in ACN) and the sample was kept in the dark for two hours. Subsequent XPS analysis (Fig. 3D, (control)) revealed no detectable F signal, confirming that no heterodimerization occurred under dark conditions.

Finally, the same sample was irradiated with UVA light (experimental details in SI Section 2.7). After irradiation, XPS analysis (Fig. 3D, (StyPFA)) displayed a new peak at 685 eV corresponding to the F 1s signal, thereby confirming the successful formation of the heterodimer on the resin surface.

We also functionalized an identical resin without coumarin with StyPFA to investigate if residual initiator enables grafting *via* radical polymerization, which resulted 0.5% fluorine content on the surface. The coumarin functionalized resin resulted in 10 times more fluorine (5.2%) (refer to SI Section 2.8), highlighting that the functionalization proceeds *via* [2 + 2] cycloaddition between coumarin and StyPFA.

## Conclusions

We investigate – for the first time – the wavelength-dependent quantum yields of a [2 + 2] photocycloaddition generating a heterodimer of 7-hydroxycoumarin (7HCou) and styrene using a photochemical action plot. The highest heterodimer yield was observed at 345 nm, red-shifted by close to 25 nm compared to the absorption maximum of 7HCou at 320 nm, likely caused by specific microenvironments leading to extended excited state lifetimes at the red-edge of the absorption spectrum.^37^

We subsequently translate these findings to photochemical surface functionalization by exploiting heterodimer formation between a surface-bound coumarin derivative and StyPFA under 345 nm irradiation – the most efficient wavelength identified from the wavelength dependent quantum yield analysis. Water contact angle measurements increased from 71° to 95° upon fluorine functionalization evidenced *via* XPS. Thus, we confirmed the successful formation of coumarin–StyPFA heterodimers on the surface *via* [2 + 2] photocycloaddition. The reversibility of surface heterodimerization was demonstrated by removing StyPFA under UVC irradiation, after which re-functionalization was successfully repeated for up to two cycles on the same surface.

Critically, we explored functionalized heterodimer formation within a dual-cure photoresin based on a polyurethane–acrylate interpenetrating network. We used an acrylate functionalized coumarin derivative to facilitate incorporation into the resin system. We found the functionalized coumarin to show a near identical absorption spectrum compared to 7HCou. We subsequently incorporated CouTEGA into a polyurethane–acrylate interpenetrating network, with FTIR analysis confirming successful incorporation. Dynamic mechanical analysis (DMA) showed no adverse effects on the mechanical properties. Indeed, slight increases were observed in both storage and loss moduli. We finally successfully functionalized the surface of the cured photoresin with a perfluorinated styrene derivative, increasing the hydrophobicity of the surface.

## Author contributions

D. K. and L. G. performed the experiments and data analysis. A. M. contributed to the NMR spectroscopic data analysis. B. T. T., K. M. and C. B.-K. conceived and designed the study and supervised the experimental work. The manuscript was written through contributions of all authors. All authors have given approval to the final version of the manuscript.

## Conflicts of interest

There are no conflicts to declare.

## Supplementary Material

SC-OLF-D5SC07978K-s001

## Data Availability

The data supporting this article have been included as part of the supplementary information (SI). Supplementary information: detailed synthetic, experimental procedures, instrumentation, materials, supporting data. See DOI: https://doi.org/10.1039/d5sc07978k.
