# Supplementary material for: Wavelength-resolved heterodimer [2 + 2] photocycloadditions for reversible surface grafting
Source: Chem Sci. 2026 Jan 27;17(13):6402–10. doi: 10.1039/d5sc07978k (PMC12879342; doi:10.1039/d5sc07978k)
Supplement: SC-017-D5SC07978K-s001 [file SC-017-D5SC07978K-s001.pdf]

Supplementary Information (SI) for Chemical Science.
This journal is © The Royal Society of Chemistry 2026

Supplementary Information

Wavelength-Resolved Heterodimer [2+2] Photocycloadditions for Reversible Surface Grafting

*Dushani Kanchana,[1] Lauren Geurds,[1] Aaron Micallef,[2] Bryan Tuten,[1,3] Kai Mundsinger,[1]**

*Christopher Barner-Kowollik[1 4]**

[1] School of Chemistry and Physics and Centre for Materials Science, Queensland University of Technology (QUT), 2 George Street, Brisbane QLD 4000, Australia. E-mail: k.mundsinger@qut.edu.au, christopher.barnerkowollik@qut.edu.au

[2] Central Analytical Research Facility, Queensland University of Technology (QUT), 2 George Street, Brisbane, QLD 4000, Australia.

[3] Department of Chemistry and Biochemistry, The University of Texas at Tyler, 3900 University Blvd., Tyler, TX 75799, United States of America

[4] Institute of Functional Interfaces (IFG), Karlsruhe Institute of Technology (KIT), Herrmann-von-Helmholtz-Platz 1, 76344 Eggenstein-Leopoldshafen, Germany. E-mail: christopher.barner-kowollik@kit.edu

Table of Contents

1 Materials and Instrumentations 4

1.1 Materials 4

1.2 Instrumentation 4

1.2.1 Nuclear Magnetic Resonance (NMR) Spectroscopy 4

1.2.2 Liquid Chromatography-Mass Spectrometry (LC-MS) 5

1.2.3 X-ray photoelectron spectroscopy (XPS) 5

1.2.4 Ultraviolet-Visible (UV-Vis) Spectroscopy 5

1.2.5 Fourier Transform Infrared (FT-IR) 6

1.2.6 Flash Column Chromatography 6

1.2.7 Photoreactor 6

1.2.8 Light Emitting Diode (LED) 6

1.2.9 Drop Shape Analyzer 7

1.2.10 Dynamic Mechanical Analysis (DMA) 7

2 Experimental Details 8

2.1 Synthesis 8

2.1.1 Synthesis of Silane Functionalized Coumarin 8

2.1.2 Synthesis of Ethyl 5-bromovalerate (C1) 8

2.1.3 Synthesis of Coumarin-ethylvalerate (C2) 9

2.1.4 Synthesis of Coumarin-ethylvaleric acid (C3) 10

2.1.5 Synthesis of Coumarin-silane (C4) 11

2.1.6 Synthesis of *para*-Styrene perfluoroalkyl ether (StyPFA) 12

2.1.7 Synthesis of TEGylated Coumarin (CouTEG) 13

2.1.8 Synthesis of Acrylate-CouTEG (CouTEGA) 14

2.2 Heterodimer formation in solution 15

2.2.1 7HCou-Styrene Heterodimer in Solution 15

2.2.2 7HCou-StyPFA Heterodimer in Solution 15

2.3 Product Distribution of Heterodimers via LC-MS 15

2.4 Heterodimer Formation on SiO_2 Surfaces **25**

2.4.1 Deposition of the Silane Functionalized coumarin (C4) on SiO_2 wafers 25

2.4.2 Cou-StyPFA Heterodimer Formation on SiO_2 Wafers 25

2.4.3 Photo-induced Cycloreversion on SiO_2 Wafers 26

2.5 Effect of UVC Irradiation on Surface Recyclability **26**

2.6 Photoresin Preparation and Curing Procedure **27**

2.7 Cou-StyPFA Heterodimer Formation on Resin Surface **28**

2.8 Photochemical Control Experiment for the Resin without CouTEGA **28**

3 Action Plot Procedures **29**

3.1 General Photochemical Action Plot Procedure[1] **29**

3.2 [2+2] Dimerization Action Plot of 7-Hydroxycoumarin-Styrene **31**

3.2.1 Heterodimer Kinetics and Action Plot 31

3.2.2 Quantum Yield (Φ) Calculations[2] 33

3.2.3 Functionalized 7-Hydroxycoumarin Extinction Spectrum 34

3.3 NMR Analysis for the Photochemical Action Plot Measurements **34**

4 Supplementary Data **40**

4.1 NMR Spectra for the Photochemical Action Plot Measurements **40**

4.2 Contact Angle Data for the Resin Samples **56**

4.3 FT-IR Spectra **57**

4.4 Dynamic Mechanical Analysis of the Resin Samples **59**

5 References **60**

1 Materials and Instrumentations

1.1 Materials

The following chemicals were used as received without further purification: Acrylic acid (99%, SIG), Bromovaleryl chloride (Combi blocks), Thionyl chloride (97%, SIG), 1-Ethyl-3-(3-dimethylaminopropyl)carbodiimide hydrochloride (98%, MERCK), 4-(Dimethylamino)pyridine (DMAP) (99%, MERCK), 1*H*,1*H*,2*H*,2*H*-Perfluoro-1-octanol (97%, MERCK), Tetrabutylammonium hydrogensulfate (TBAH) (97%, Merck), 4-Vinylbenzyl chloride (90%, ACROS Organics), 3-Aminopropyl triethoxysilane (APTES) (98%, MERCK), Poly(ethylene glycol) diacrylate (PEGDA, Mn 250, MERCK), Di(ethylene glycol) diacrylate (DEGDA, 75%, MERCK), Phenylbis(2,4,6-trimethylbenzoyl)phosphine oxide (BAPO, 97%, MERCK), Clear Flex™ 50 (Smooth-On inc.), Sodium hydroxide (AR Grade, AJAX), Potassium carbonate (Chem Supply), Potassium iodide (Chem Supply), Magnesium sulphate (98%, MERCK), Hydrochloric acid (32%, AJAX), Triethylamine (TEA, 99.5%, Merck), 2-[2-(2-Chloroethoxy)ethoxy]ethanol (96%, Combi blocks), 7-Hydroxycoumarin (98%, Combi blocks), Dichloromethane (DCM, Thermo Fisher Scientific, analytical grade), Dimethylformamide (DMF, Thermo Fisher Scientific), Ethyl acetate (EA) (Thermo Fisher Scientific), Methanol (Thermo Fisher Scientific), Ethanol (AJAX), Chloroform (Thermo Fisher Scientific), Acetone (AJAX, analytical grade), Acetonitrile (ACN, HPLC grade, VWR), ACN-d_3 (99.8%, Merck), $CDCl_3$ (99.8%, NOVACHEM).

Acryloyl chloride was synthesized from acrylic acid and thionyl chloride using the following procedure. Thionyl chloride (0.9 eq) was added under argon atmosphere to a round bottom flask in an ice bath containing acrylic acid (1 eq) and a catalytic amount of DMF. The addition rate was controlled to keep the gas evolution (bubbling) constant and not too vigorous. Once no more bubbles were observed, the flask was heated to approximately 100 °C and the mixture was distilled at a rapid rate. The distillate was collected in a receiver flask containing half a gram of hydroquinone, immersed in ice. When the temperature at the top of the column which remained between 70 °C and 90 °C for most of the distillation had reached 100 °C, the distillation was discontinued. The crude product was then redistilled through the same column and the fraction boiling at 85 °C was collected and stored under N_2 at ambient temperature.

1.2 Instrumentations

1.2.1 Nuclear Magnetic Resonance (NMR) Spectroscopy

^1H NMR spectra were recorded at 298 K on either a 600 MHz Bruker Avance IIIHD spectrometer, equipped with a BBO-Probe (5 mm) with z-gradient (^1H: 600.13 MHz, ^{13}C 150.90 MHz), or a 400 MHz Bruker Neo spectrometer, equipped with a TBO-Probe (5 mm) with a dedicated ^{19}F channel and z-gradient (^1H: 400.14 MHz, ^{13}C 100.62 MHz) . Resonances are reported in parts per million (ppm)

relative to tetramethylsilane (TMS). The δ-scale was calibrated to the respective solvent signal of residual $CHCl_3$ or CH_3CN for ^1H spectra.

COSY spectra were recorded at 600 MHz to assess the formation of hetero- and homodimers obtained during irradiation of mixtures of 7-hydroxycoumarin and styrene. Due to the low yield of dimers in the action plot experiments, the COSY experiment was optimised to obtain a reasonable signal to noise ratio for the cyclobutane resonances, using the Bruker COSYGPSW parameter set with ns=100, 2k data points in F2 and 128 slices in F1, giving a 7.3 h total measurement time. F2 and F1 were zero filled to 4k and 1k respectively and linear prediction was applied to F1.

1.2.2 Liquid Chromatography-Mass Spectrometry (LC-MS)

LC-MS measurements were performed on a Dionex UltiMate 3000 UHPLC system consisting of a quaternary pump (LPG 3400RS), autosampler (WPS 3000TRS) and column oven (TCC 3000). A 10 µL aliquot of sample was injected onto a C18 HPLC column (Phenomenex Luna 5 µm, 100 Å, 250 × 2.0 mm) maintained at 40 °C. Mobile phase A was 5 mM ammonium acetate in water and mobile phase B was acetonitrile. The combined flow rate was maintained at 0.4 $mL \cdot min^{-1}$. After an initial 0.6 min isocratic period at 20% B, the gradient was increased to 95% B over 7 min then held at 95% B for a further 3 min before returning to 20% B. The eluate was directed to a UV diode array detector (DAD 3000, Dionex) and subsequently into the heated electrospray ionisation source of a Q Exactive Plus Biopharma high-resolution Orbitrap mass spectrometer (Thermo Fisher Scientific, Bremen, Germany). The spray voltage was set to 3.0 kV, and the sheath and auxiliary gas (N_2) flow rates were set to 30 and 10 (dimensionless arbitrary units), respectively. The capillary temperature was set to 320 °C, the S-lens RF level was set to 60, and the auxiliary gas heater temperature was set to 150 °C. Spectra were acquired at a nominal mass resolving power of 70,000 (defined at *m/z* 200).

1.2.3 X-ray photoelectron spectroscopy (XPS)

XPS spectra were collected using a Kratos Axis Supra system operating with a monochromatic Al Kα source (1486.7 eV). Survey spectra and high-resolution core-level spectra were collected with pass energies of 160 and 20 eV respectively and a step size of 0.1 eV. All XPS data were processed with CasaXPS. Each sample was measured at three different locations on the sample to ensure representative sampling.

1.2.4 Ultraviolet-Visible (UV-Vis) Spectroscopy

UV-Vis spectra were recorded on a Shimadzu UV-2700 spectrophotometer equipped with a CPS-100 electronic temperature control cell positioner. Samples were prepared in acetonitrile and measured in a Hellma Analytics quartz high precision cell (108-F-10-40) with a path length of 10 mm at ambient temperature.

1.2.5 Fourier Transform Infrared (FT-IR)

All IR measurements were performed on a *Bruker* Alpha-P ATR-IR from 500-4000 cm^{-1} at ambient temperature.

1.2.6 Flash Column Chromatography

Flash column chromatography was performed on a CombiFlash Rf+ (*Teledyne ISCO*). Fractions were collected based on a UV detector (254, 320 nm and detection of the full UV-Vis spectrum). *Interchim* Puriflash 15 and 30 µm Silica-HP columns were used for the separations. The raw products were unless otherwise noted deposited on Celite® 565 by dissolving, mixing and evaporation off the volatiles under reduced pressure. Subsequently, the Celite® was transferred into a Teledyne plunger pre-column filled to 1/3 with aspherical silica gel 35-60 µm.

1.2.7 Photoreactor

A *Luzchem* LZC-4V photoreactor using LZC-UVA or LZC-UVC lamps was used for the curing of the resin or photo-induced cycloreversion. The emission spectrum is depicted in **Figure S1** (emission maximum close to 350 nm and 254 nm). The internal chamber was ventilated to maintain ambient temperature during the entire experiment.

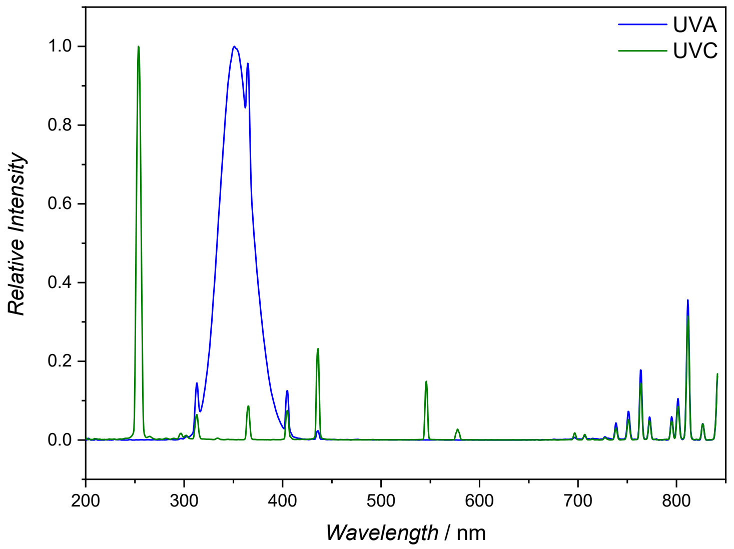

Figure S1 Emission spectrum of the UVA lamps (350 nm, blue) and UVC (254 nm, green) lamp used in photoreactor.

1.2.8 Light Emitting Diode (LED)

For the photoreactions a λ_{max} = 323 nm LED and a λ_{max} = 342 nm LED were employed. The LED emission spectra can be found in **Figure S2**. For the LED characterization (power/irradiance), a Thorlabs PM400 with an SC401C sensor head and Ocean Optics FLAME-T-UV-VIS spectrometers were used. LEDs were cooled during measurement to minimize any thermal effects on the emission power or sensor performance.

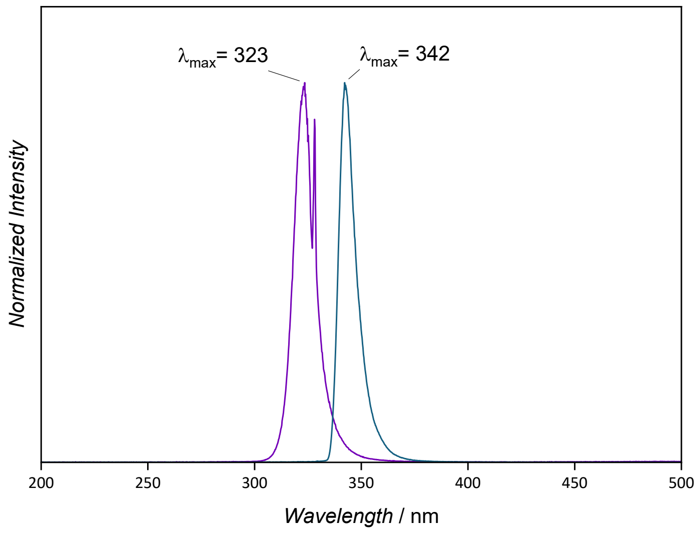

Figure S2 Emission spectra of the two LEDs (325 nm and 345 nm) employed for irradiation.

1.2.9 Drop Shape Analyzer

Contact angles were measured using a Drop Shape Analyzer (Theta Flex optical tensiometer). The instrument was equipped with a USB 3 camera, and image method was set at 331 frames per second with a resolution of 1216 × 800 pixels. A side-view optical arrangement was employed, and illumination was provided by a monochromatic LED backlight. Test liquid (deionized water) was dispensed via a 200 µL plastic dispenser tip. Drop volume was controlled to be 5 µL and the rate of dispensing was 20 µL/s. After deposition, the drop was allowed to equilibrate for 10 seconds before acquiring the image. Measurements were conducted at ambient temperature (close to 25 °C) with a horizontally mounted surface. Static contact angles (sessile drop) were determined by fitting the drop profile using the Young-Laplace model via the software OneAttension.

1.2.10 Dynamic Mechanical Analysis (DMA)

DMA was performed using TA instruments discovery DMA 850 to evaluate the viscoelastic properties of the cured photoresins. Rectangular specimens with dimensions between 12 × 4.5 × 1.5 mm were prepared and mounted in the tensile geometry. Prior to frequency sweeps, an amplitude sweep was performed at ambient temperature and 1 Hz to identify the linear viscoelastic region (LVR). Subsequent frequency sweeps were carried out within the LVR over the range of 0.1–10 Hz at a fixed strain amplitude of 0.0016. Storage modules (*G*′) and loss modules (*G*″) were recorded throughout all experiments.

2 Experimental Details

2.1 Synthesis

2.1.1 Synthesis of Silane Functionalized Coumarin

Silane functionalized coumarin was synthesized through a 4-step synthesis procedure, which includes one esterification reaction, one etherification reaction, one amide coupling reaction and simple hydrolysis reaction as shown in the below scheme.

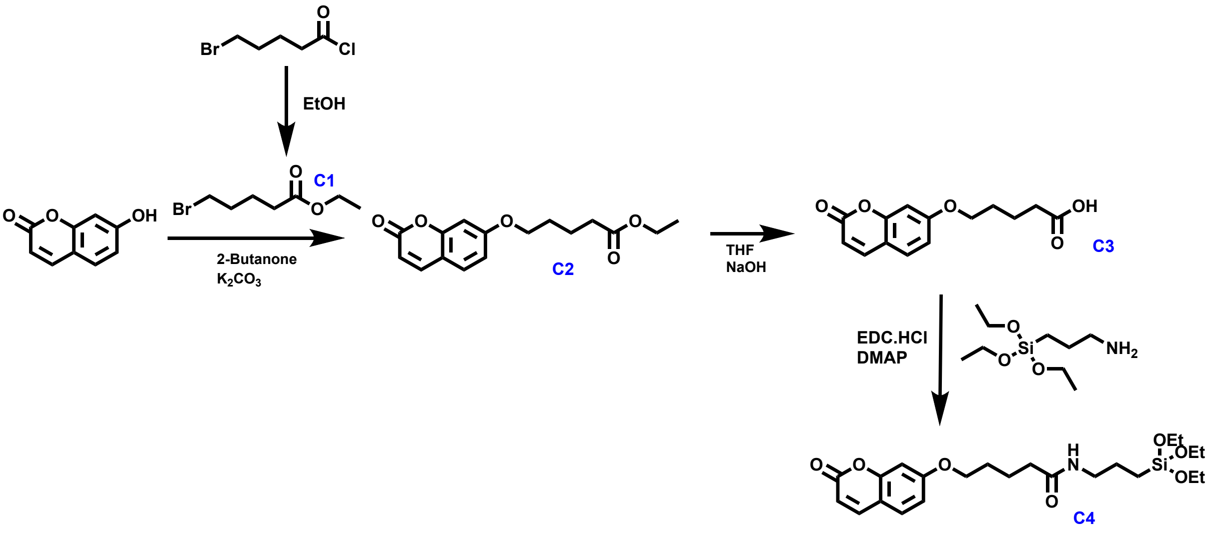

Figure S3 Synthesis route for the silane functionalized coumarin (C4).

2.1.2 Synthesis of Ethyl 5-bromovalerate (C1)

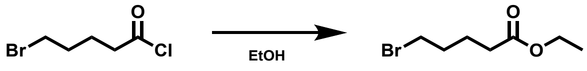

Bromovaleryl chloride (5 mL,37.5 mmoL, 1 eq) was added to 100 mL of ethanol (100 mL, 1693 mmoL, 45 eq) in dry conditions with a small amount of silica beads in the reaction mixture. The solution was stirred overnight at 35 °C temperature. After the completion of reaction, solvent was removed under reduced pressure and pale green (humidity indicator leached from the silica beads) oily liquid was obtained. Yield: 78% (^{1}H NMR spectrum in **Figure S4**)

^{1}H NMR (600 MHz, $CDCl_3$): δ = 4.07 (q, 2H, CH_2), 3.35 (t, 2H, CH_2), 2.27 (t,2H, CH_2), 1.88 – 1.80 (m, 2H, CH_2), 1.72 (tt, 2H, CH_2), 1.19 (t, 3H, CH_3) ppm.

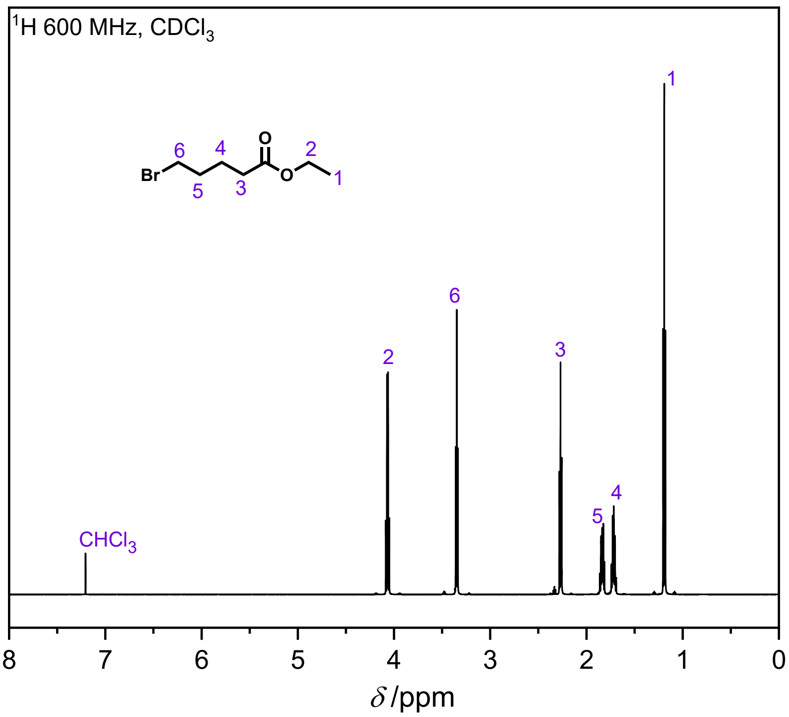

Figure S4 ^{1}H NMR (600 MHz) of ethyl 5-bromovalerate (C1) in $CDCl_3$.

2.1.3 Synthesis of Coumarin-ethylvalerate (C2)

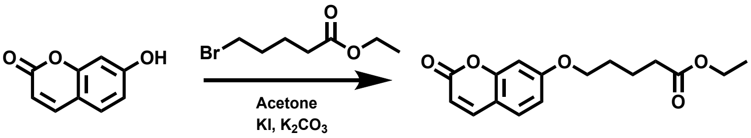

7-Hydroxycoumarin (4.62 g, 28.41 mmol, 1.0 eq), ethyl 5- bromovalerate (5.94 g, 28.41 mmol, 1.0 eq), K_2CO_3 (12 g, 87 mmol, 3.1 eq), KI (50 g, 360 mmol, 12 eq) and 150 mL acetone were added to a round-bottom flask. The reaction mixture was refluxed at 61 °C for 48 h. After the reaction, the solid was filtered off and the solvent was removed under reduced pressure to afford a yellow solid. The solid was dissolved in ethyl acetate (200 mL) and then washed with HCl 3N (200 mL ×3), brine (200 mL), and dried over Mg_2SO_4. Ethyl acetate was removed under reduced pressure to afford a yellow solid. Yield: 69% (^{1}H NMR spectrum in **Figure S5**).

^{1}H NMR (600 MHz, $CDCl_3$): δ = 7.56 (dd, 1H, H-aromatic), 7.29 (d, 1H, H-aromatic), 6.76 (dd, 2H, H-aromatic), 6.18 (d, 1H, H-aromatic), 4.07 (q, 2H,), 3.97 (t, 2H, CH_2), 2.33 (t, 2H CH_2), 1.84 – 1.72 (m, 4H,), 1.20 (t, 3H, CH_3)

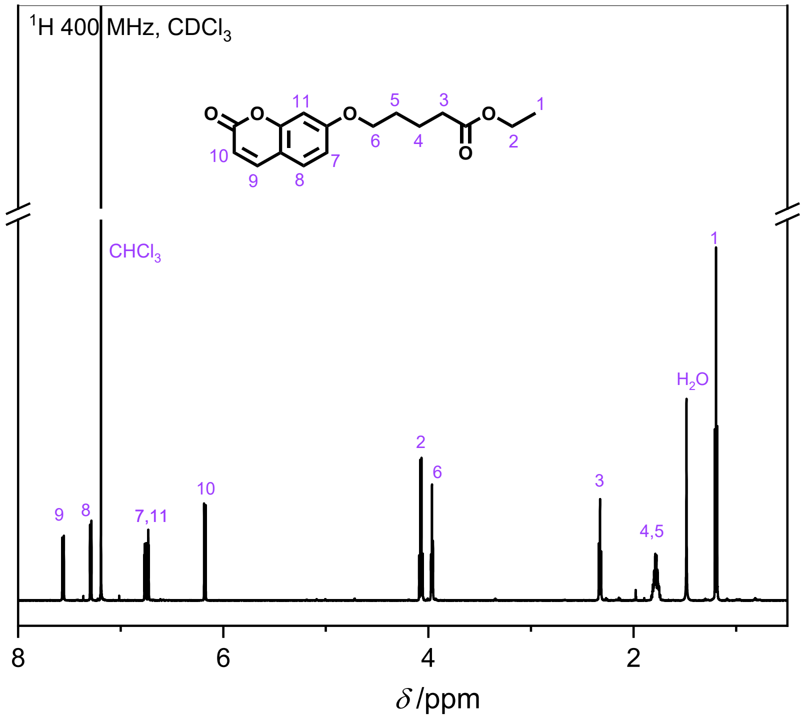

Figure S5 ^{1}H NMR (600 MHz) of coumarin-ethyl valerate (C2) in $CDCl_3$.

2.1.4 Synthesis of Coumarin-ethylvaleric acid (C3)

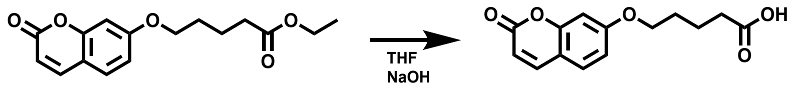

A solution of ester (3.34 g, 11.5 mmol) in dry THF (7 mL), water (12 mL), and 1M NaOH (12 mL) was heated to reflux overnight. Then the solvent was removed under reduced pressure, and the aqueous layer was washed with $CHC1_3$ (40 mL, twice). The aqueous layer was acidified with 2M HCl and extracted with $CHCl_3$. The organic layer was washed with brine and subsequently dried with $MgSO_4$. Yield: 73% (^{1}H NMR spectrum in **Figure S6**).

^{1}H NMR (600 MHz, DMSO-d_6): δ = 7.59 – 7.54 (m, 1H, H-aromatic), 7.30 (d, 1H, H-aromatic), 6.79 – 6.72 (m, 2 H, H-aromatic), 6.18 (d, 1H, H-aromatic), 3.98 (t, 2 H CH_2), 2.40 (t, 2H, CH_2), 1.87 – 1.74 (m, 4H, CH_2).

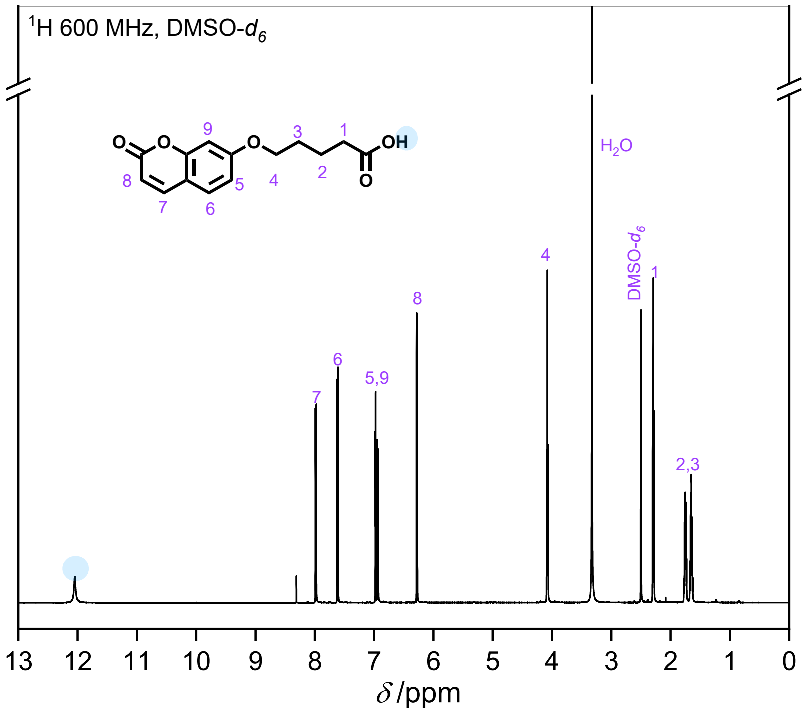

Figure S6 ^{1}H NMR (600 MHz) of coumarin-ethyl valeric acid (C3) in DMSO-d_6.

2.1.5 Synthesis of Coumarin-silane (C4)

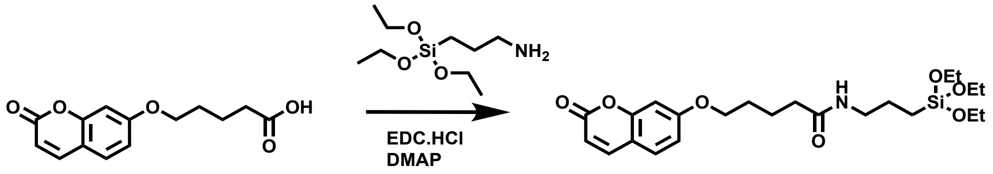

Coumarin-ethylvaleric acid (2.15 g, 8.19 mmol), EDC*HCl (2.49 g, 12.98 mmol) and DMAP (4.31 mL,31.97 mmol) were dissolved in dry DCM 220 mL in Schlenk flask under dry conditions. 3-Aminopropyl triethoxysilane (APTES) (2.10 mL,9.01 mmol) was added drop wise to the flask while stirring. The mixture was stirred overnight at ambient temperature. The generated solid was filtered off, and the solvent was washed twice with bicarb, twice with water and once with brine and dried with $MgSO_4$. DCM was removed under reduced pressure, and a yellow solid was obtained. Yield:65% (^{1}H NMR spectrum in **Figure S7**).

^{1}H NMR (600 MHz, $CDCl_3$): δ = 7.57 (d, 1 H, H-aromatic), 7.29 (d, 1H, H-aromatic), 6.78 – 6.70 (m, 2H, H-aromatic), 6.18 (d, 2H, H-aromatic), 3.99 (t, 2H, CH_2), 3.75 (q, 6H, -CH_2CH_3), 3.25 – 3.14 (m, 2H, -CH2), 2.22 – 2.15 (t, 2H, -CH_2), 1.78 (dd, 4H, -CH_2), 1.57 (ddt, 2H -CH_2) 1.23 – 1.11 (q, 9H,-CH_3) 0.60 – 0.54 (m, 2H, CH_2).

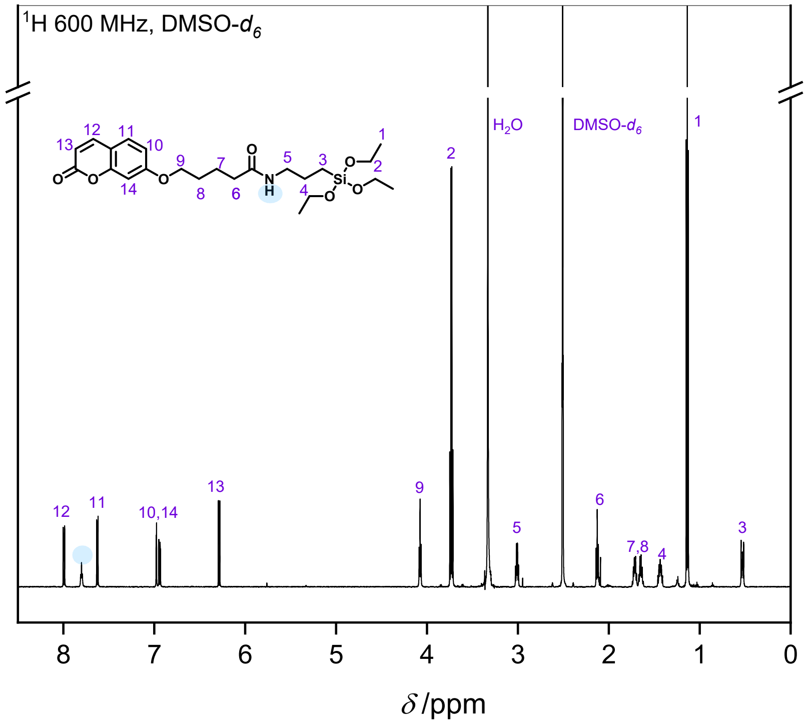

Figure S7 ¹H NMR (600 MHz) of coumarin-silane (C4) in DMSO-d_6.

2.1.6 Synthesis of *para*-Styrene perfluoroalkyl ether (StyPFA)

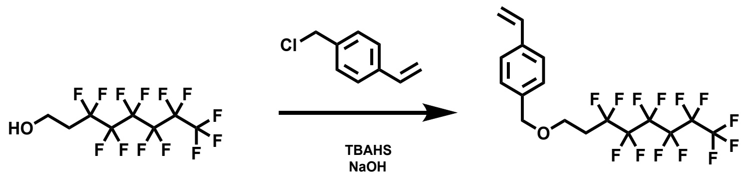

A solution of 0.5 g (1.38 mmol) of 1*H*,1*H*,2*H*,2*H*-perfluoro-1-octanol, 0.48 g (1.38 mmol) of tetrabutylammonium hydrogensulfate (TBAH) and 20 mL of 6M NaOH solution was vigorously stirred at 50 °C temperature for one hour. Subsequently, 4-vinyl benzylchloride 0.210 g (1.38 mmol) was added dropwise to the solution and stirred for an additional 12 hours. After the completion of reaction, 20 mL of CH_2Cl_2 and 20 mL of water were added to the solution, and the organic layer washed four times with an aqueous solution of 1M HCl. The organic layer was dried over $MgSO_4$ and filtered. The solvent was removed by a rotary evaporator to obtain a yellow oily product. The crude compound was purified by reverse phase C18-silica gel chromatography using a water to acetonitrile solvent gradient (starting from 50:50 acetonitrile: water changing to 100% acetonitrile) Yield: 63% (¹H NMR spectrum shown in **Figure S8**).

¹H NMR (600 MHz, $CDCl_3$): δ = δ 7.37 – 7.29 (m, 1H), 7.23 (dd, *J* = 20.5, 8.1 Hz, 1H), 6.64 (dd, *J* = 17.6, 10.9, 2.4 Hz, 1H), 5.71 – 5.64 (m, 0H), 5.22 – 5.14 (m, 1H), 4.46 (d, *J* = 7.6 Hz, 1H), 3.68 (t, *J* = 6.8 Hz, 1H), 2.42 – 2.30 (m, 1H).

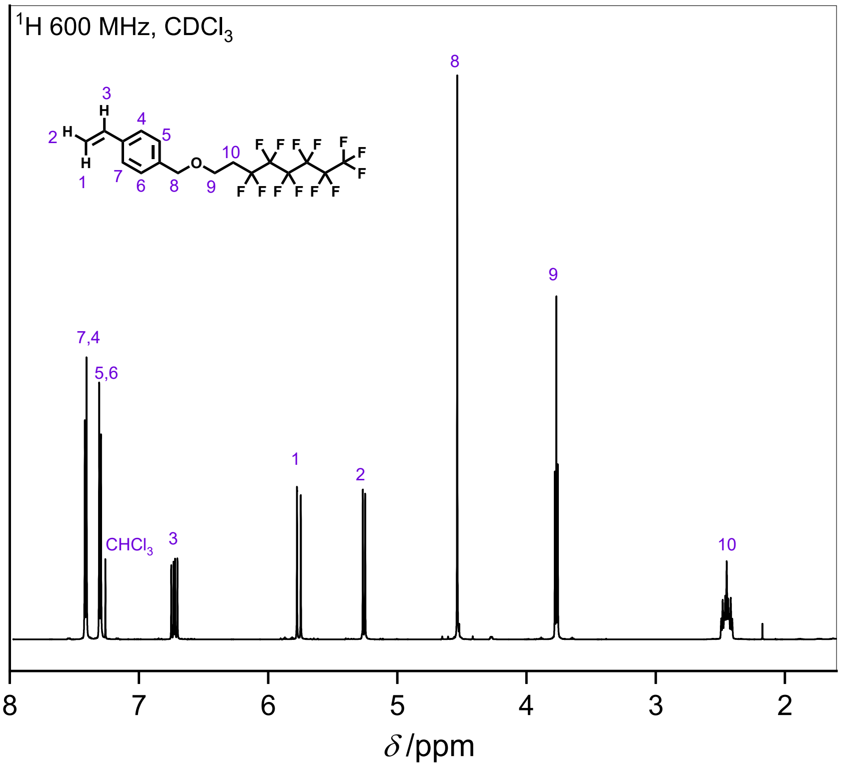

Figure S8 ^{1}H NMR (600 MHz) of StyPFA in CDCl$_3$.

2.1.7 Synthesis of TEGylated Coumarin (CouTEG)

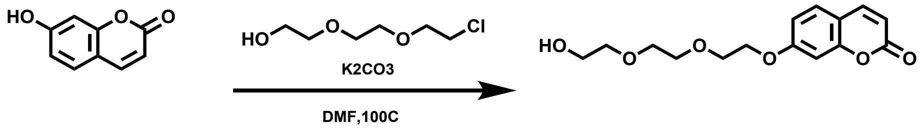

A round bottom flask was charged with 2-[2-(2-chloroethoxy) ethoxy] ethanol (5.38 g, 37 mmol, 1.2 eq.), 7-hydroxycoumarin (5 g, 30 mmol, 1 eq.) and potassium carbonate (2 eq.) and 20 mL DMF. The reaction was heated to 100 °C and refluxed overnight. Upon reaction completion, the mixture was cooled to room temperature, filtered and concentrated under reduced pressure. The pure product was obtained by flash chromatography using a gradient of DCM: EA 70:30 to 0:100, *v/v*. The product was obtained as a white powder. Yield:80% (^{1}H NMR spectrum shown in **Figure S9**).

^{1}H NMR (400 MHz, CDCl$_3$): δ (ppm) = δ 7.62 (s, 1H), 7.36 (s, 1H), 6.86 (d, J = 23.2 Hz, 2H), 6.26 (s, 1H), 4.20 (s, 2H), 3.90 (s, 2H), 3.79 – 3.56 (m, 9H), 1.85 (s, 2H).

.

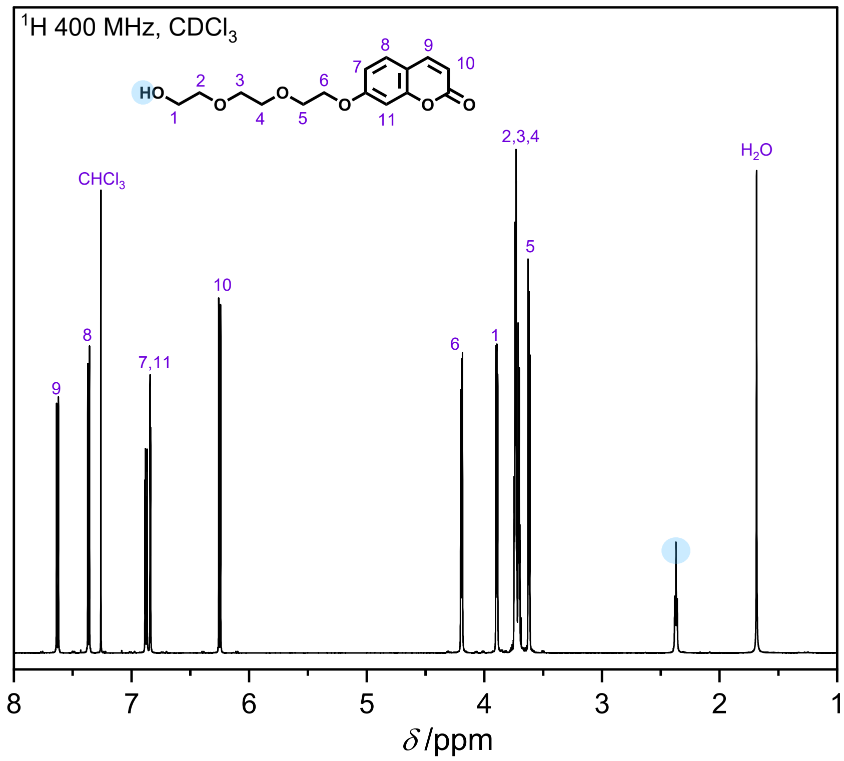

Figure S9 ^{1}H NMR (400 MHz) of CouTEG in $CDCl_3$.

2.1.8 Synthesis of Acrylate-CouTEG (CouTEGA)

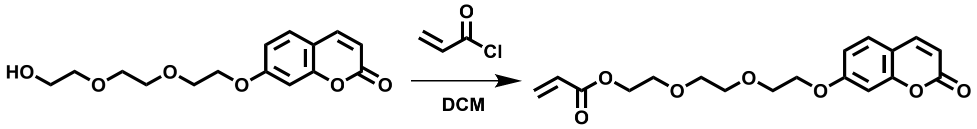

7-(2-(2-(2-Hydroxyethoxy)ethoxy)ethoxy)-2H-chromen-2-one (1.92 g, 6.43 mmol, 1.00 eq.), was dissolved in DCM (30 mL) under argon. The reaction mixture was cooled and kept at close to 0 °C for the addition of TEA (7.18 mmol, 1.1 eq) and the dropwise addition of acryloyl chloride (7.18 mmol, 1.1 eq). The solution was left overnight at ambient temperature. The pure product was obtained by flash chromatography using a gradient of dichloromethane: ethyl acetate 80:20 to 0:100, *v/v*. The product was obtained as an off-white powder Yield: 68% (^{1}H NMR spectrum shown in **Figure S10**).

^{1}H NMR (400 MHz, $CDCl_3$) δ 7.63 (dd, *J* = 9.5, 0.6 Hz, 1H), 7.36 (d, *J* = 8.6 Hz, 1H), 6.90 – 6.80 (m, 2H), 6.42 (dd, *J* = 17.3, 1.5 Hz, 1H), 6.25 (d, *J* = 9.5 Hz, 1H), 6.14 (dd, *J* = 17.3, 10.4 Hz, 1H), 5.83 (dd, *J* = 10.5, 1.4 Hz, 1H), 4.35 – 4.27 (m, 2H), 4.21 – 4.15 (m, 2H), 3.92 – 3.85 (m, 2H), 3.78 – 3.65 (m, 6H).

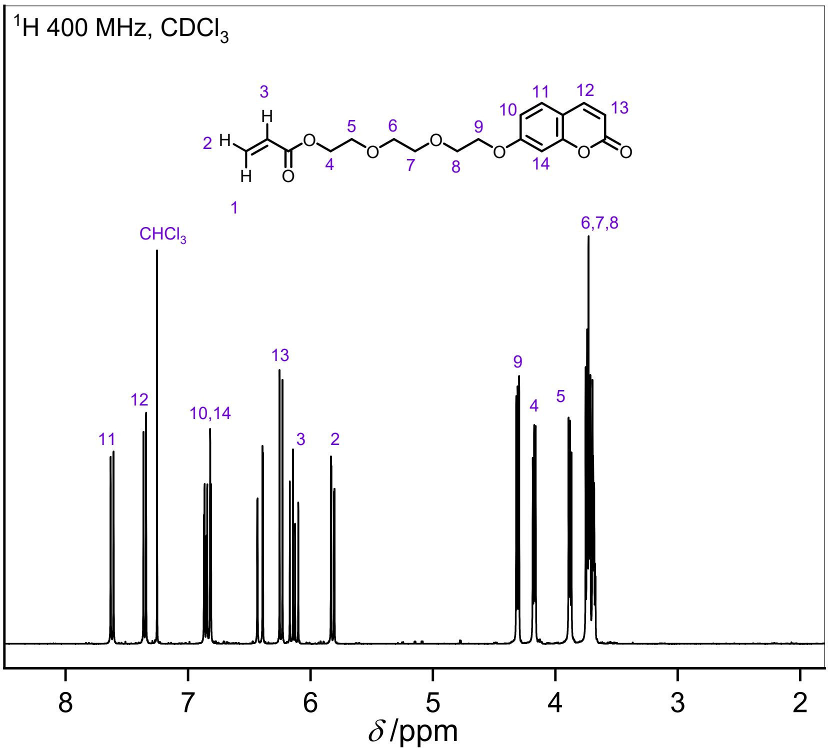

Figure S10 ^{1}H NMR (400 MHz) of CouTEGA in $CDCl_3$.

2.2 Heterodimer formation in solution

2.2.1 7HCou-Styrene Heterodimer in Solution

For the preliminary reaction assessment, 10 mg of 7HCou and 1 mL of styrene were dissolved in 1 mL of acetonitrile in a crimped vial. The solution was degassed for 10 min and irradiated for 1 hour under a 325 nm LED (emission spectrum shown in Section 1.2.8). The irradiated sample was characterized by LC-MS and ^{1}H NMR spectroscopy.

2.2.2 7HCou-StyPFA Heterodimer in Solution

0.7 mg of 7HCou and 46 mg of StyPFA were dissolved in 1 mL of acetonitrile. The solution was degassed for 10 min and irradiated under a 345 nm LED for 1 hour (emission spectrum shown in Section 1.2.8) The irradiated sample was characterized by LC-MS and ^{1}H NMR spectroscopy.

2.3 Product Distribution of Heterodimers via LC-MS

While heterodimer formation was confirmed by ^{1}H NMR spectroscopy, the presence of homodimer and regioisomers could not be evaluated due to the low overall conversion and significant signal overlap. Specifically, the limited concentration of the product and overlapping resonances prevented

reliable quantification and definite assignment of individual species. To overcome these limitations, LC–MS was employed. LC–MS provided corresponding *m/z* values, indicating the presence of both homo- and heterodimer species formed during the irradiation at various wavelengths, as demonstrated in **Figure S11** and **Figure S12** (irradiated at 310 nm). However, since the extinction coefficient of the homodimer could not be determined, the ratio between homo- and heterodimer formation could not be quantified. However, the LC trace clearly demonstrates peaks of both homo- and heterodimer, further supporting the hypothesized formation of regioisomers. Similarly, the inability to isolate these regioisomer due to the low reaction efficiency, prevents identification and quantitative analysis of the specific regioisomers and their distribution.

Further, the formation of homo- and heterodimer, as well as regioisomers was likewise observed during the [2+2] cycloaddition between CouTEG and styrene.

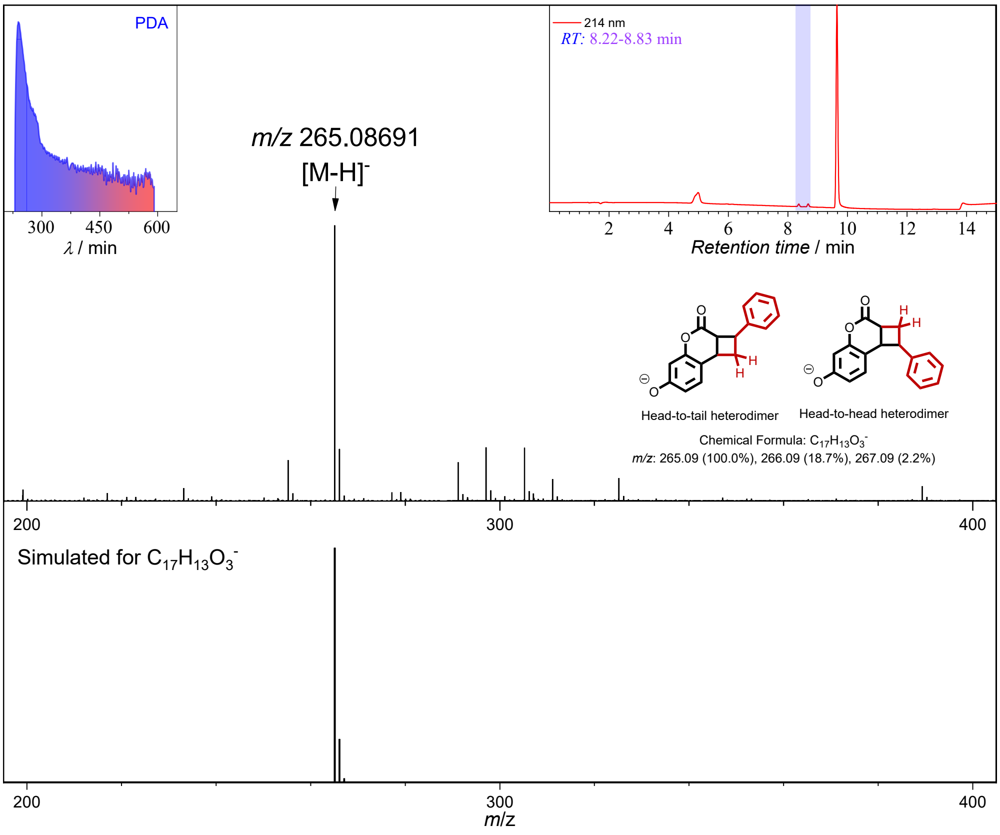

Figure S11 LC trace (214 nm detector wavelength) of 7-hydroxycoumarin with styrene in ACN irradiated at 310 nm for 1 h, and corresponding mass spectrum for the retention time 8.22-8.83 min and the simulated mass spectrum (bottom) for the heterodimer.

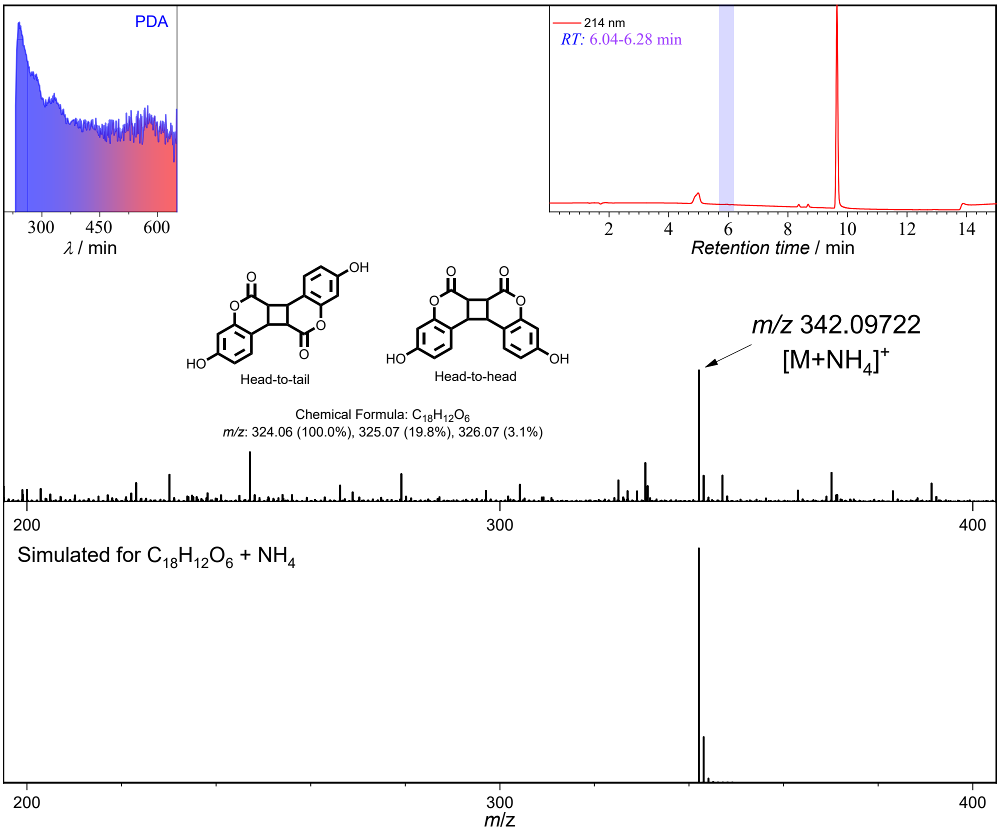

Figure S12 LC trace (214 nm detector wavelength) of 7-hydroxycoumarin with styrene in ACN irradiated at 310 nm for 1 h, and corresponding mass spectrum for the compound at retention time 6.04-6.28 min (top) and the simulated mass spectrum (bottom) for the homodimer.

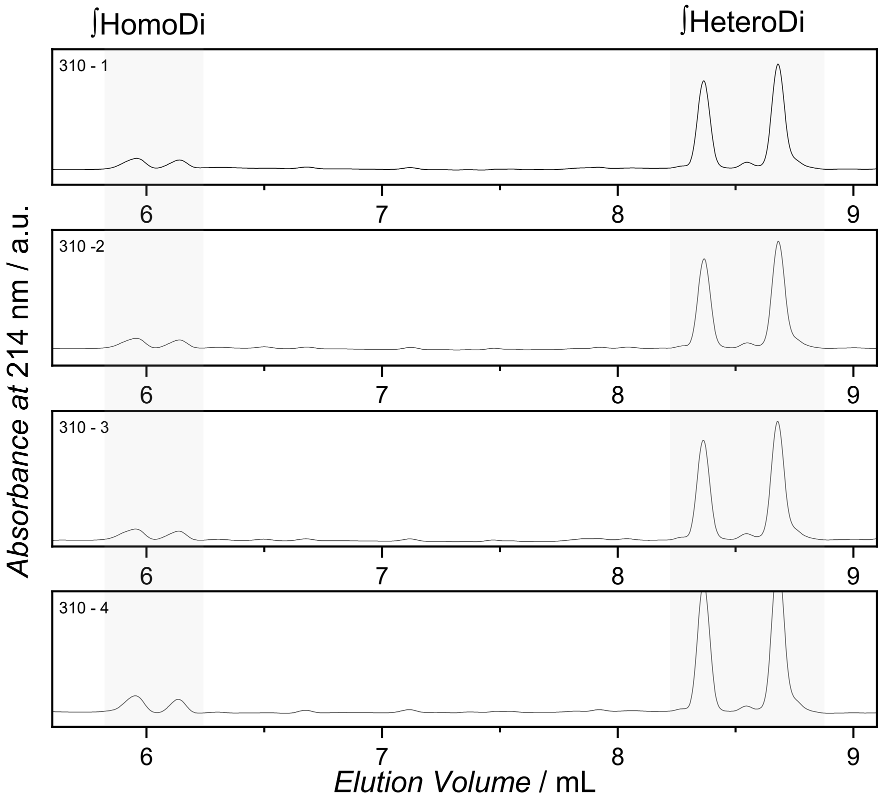

Figure S13 Representative LC traces (214 nm detector wavelength) of the [2+2] dimerization of 7-hydroxycoumarin with styrene in ACN, irradiated at 310 nm for 1 h. Four replicates are shown to demonstrate reproducibility. Elution volumes corresponding to the homodimer (6.04–6.28 min) and heterodimer (8.22–8.83 min) are highlighted.

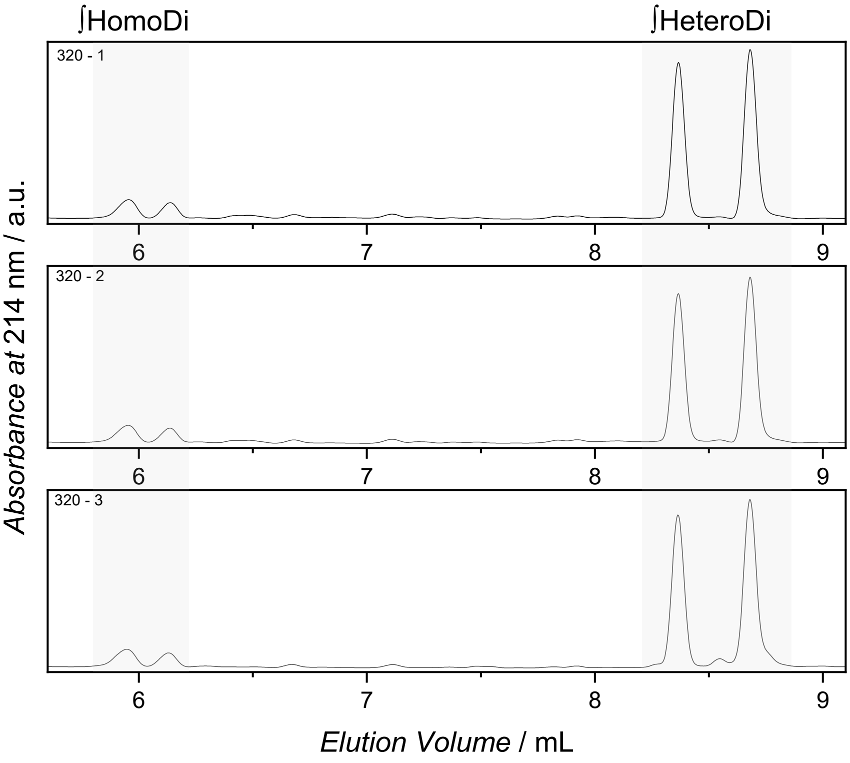

Figure S14 Representative LC traces (214 nm detector wavelength) of the [2+2] dimerization of 7-hydroxycoumarin with styrene in ACN, irradiated at 320 nm for 1 h. Three replicates are shown to demonstrate reproducibility. Elution volumes corresponding to the homodimer (6.04–6.28 min) and heterodimer (8.22–8.83 min) are highlighted.

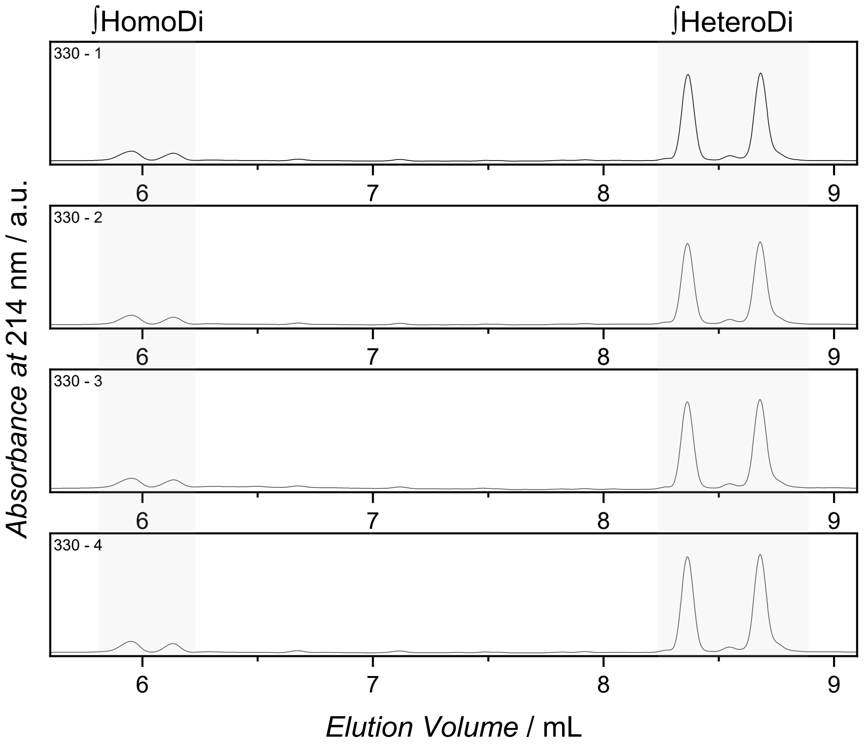

Figure S15 Representative LC traces (214 nm detector wavelength) of the [2+2] dimerization of 7-hydroxycoumarin with styrene in ACN, irradiated at 330 nm for 1 h. Four replicates are shown to demonstrate reproducibility. Elution volumes corresponding to the homodimer (6.04–6.28 min) and heterodimer (8.22–8.83 min) are highlighted.

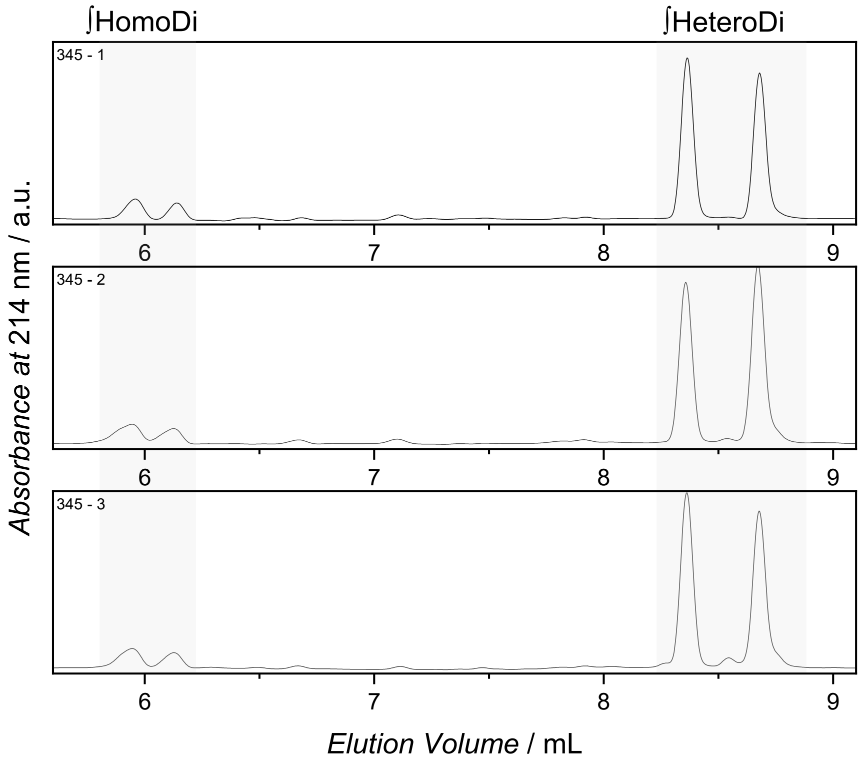

Figure S16 Representative LC traces (214 nm detector wavelength) of the [2+2] dimerization of 7-hydroxycoumarin with styrene in ACN, irradiated at 345 nm for 1 h. Three replicates are shown to demonstrate reproducibility. Elution volumes corresponding to the homodimer (6.04–6.28 min) and heterodimer (8.22–8.83 min) are highlighted.

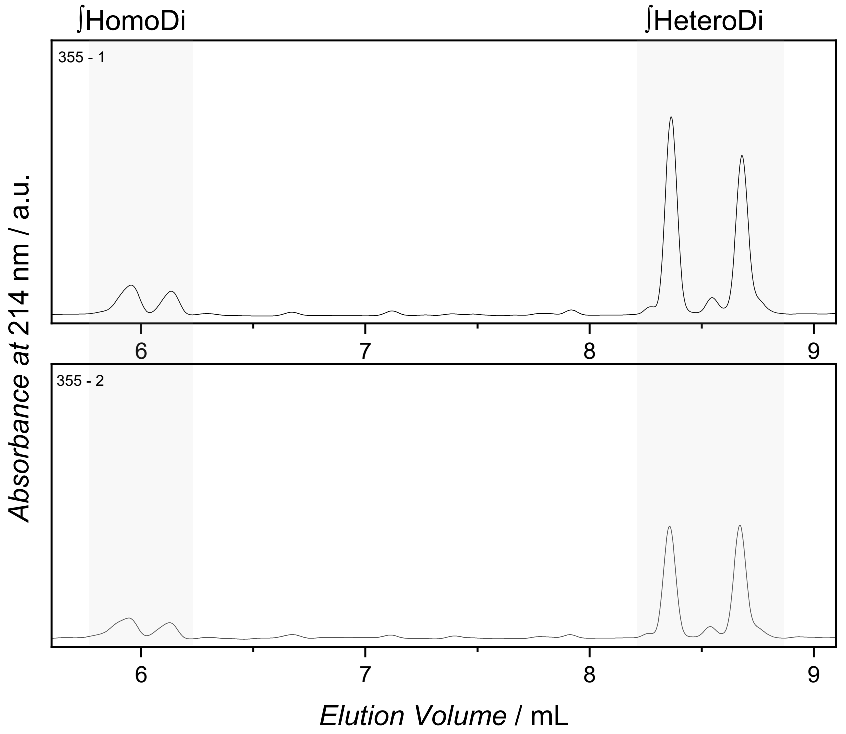

Figure S17 Representative LC traces (214 nm detector wavelength) of the [2+2] dimerization of 7-hydroxycoumarin with styrene in ACN, irradiated at 355 nm for 1 h. Two replicates are shown to demonstrate reproducibility. Elution volumes corresponding to the homodimer (6.04–6.28 min) and heterodimer (8.22–8.83 min) are highlighted.

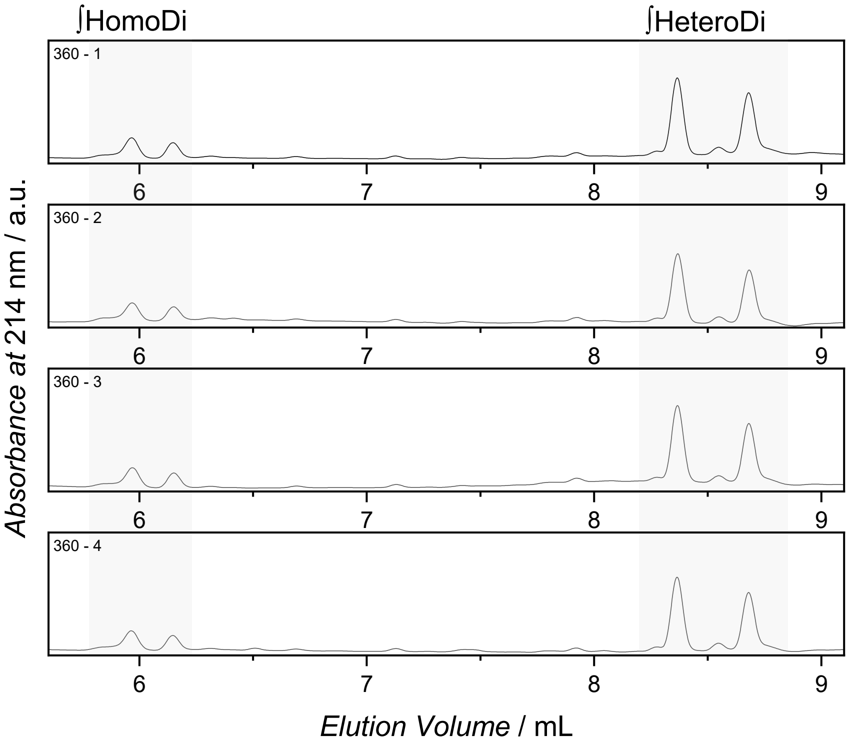

Figure S18 Representative LC traces (214 nm detector wavelength) of the [2+2] dimerization of 7-hydroxycoumarin with styrene in ACN, irradiated at 360 nm for 1 h. Four replicates are shown to demonstrate reproducibility. Elution volumes corresponding to the homodimer (6.04–6.28 min) and heterodimer (8.22–8.83 min) are highlighted.

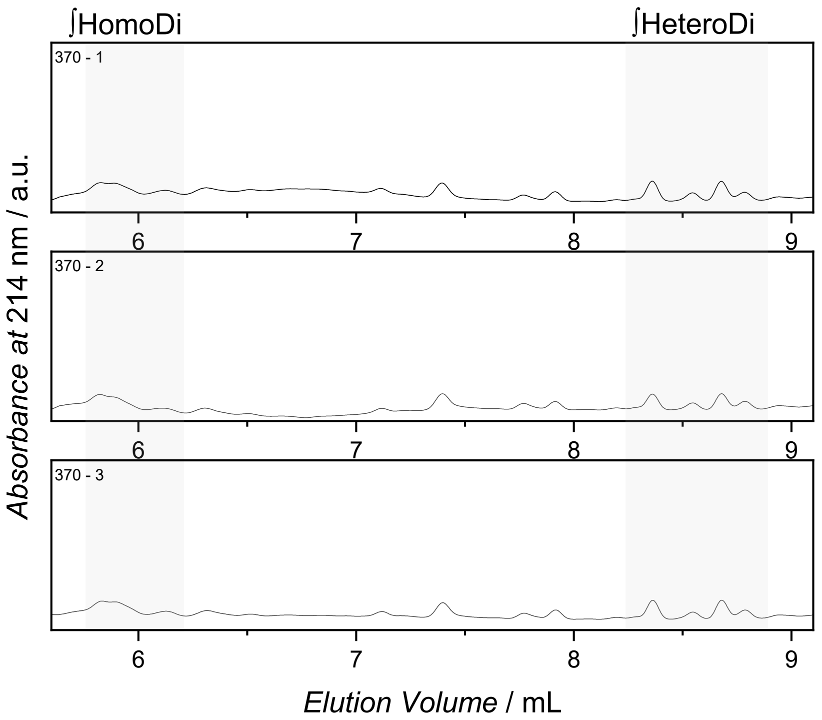

Figure S19 Representative LC traces (214 nm detector wavelength) of the [2+2] dimerization of 7-hydroxycoumarin with styrene in ACN, irradiated at 370 nm for 1 h. Three replicates are shown to demonstrate reproducibility. Elution volumes corresponding to the homodimer (6.04–6.28 min) and heterodimer (8.22–8.83 min) are highlighted.

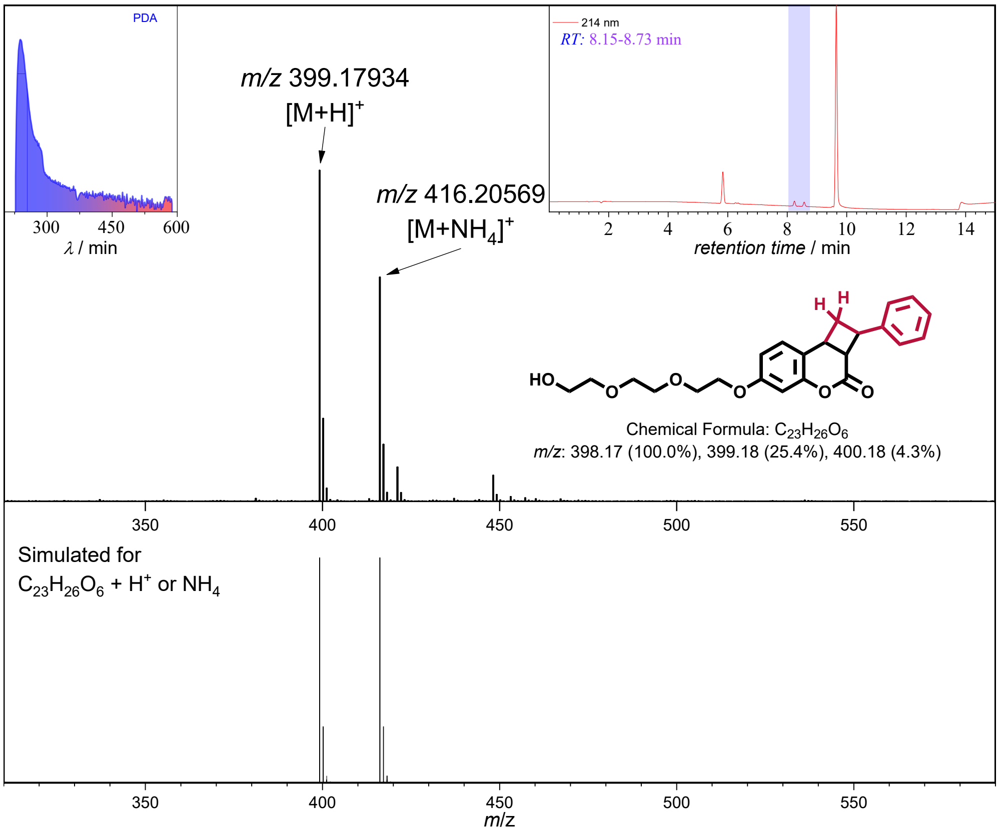

Figure S20 LC trace (214 nm detector wavelength) of CouTEG with styrene in ACN irradiated at 320 nm for 1 h, and corresponding mass spectrum for the heterodimer at retention time 8.15-8.73 min(top) and simulated mass spectrum (bottom) for the CouTEG-styrene heterodimer.

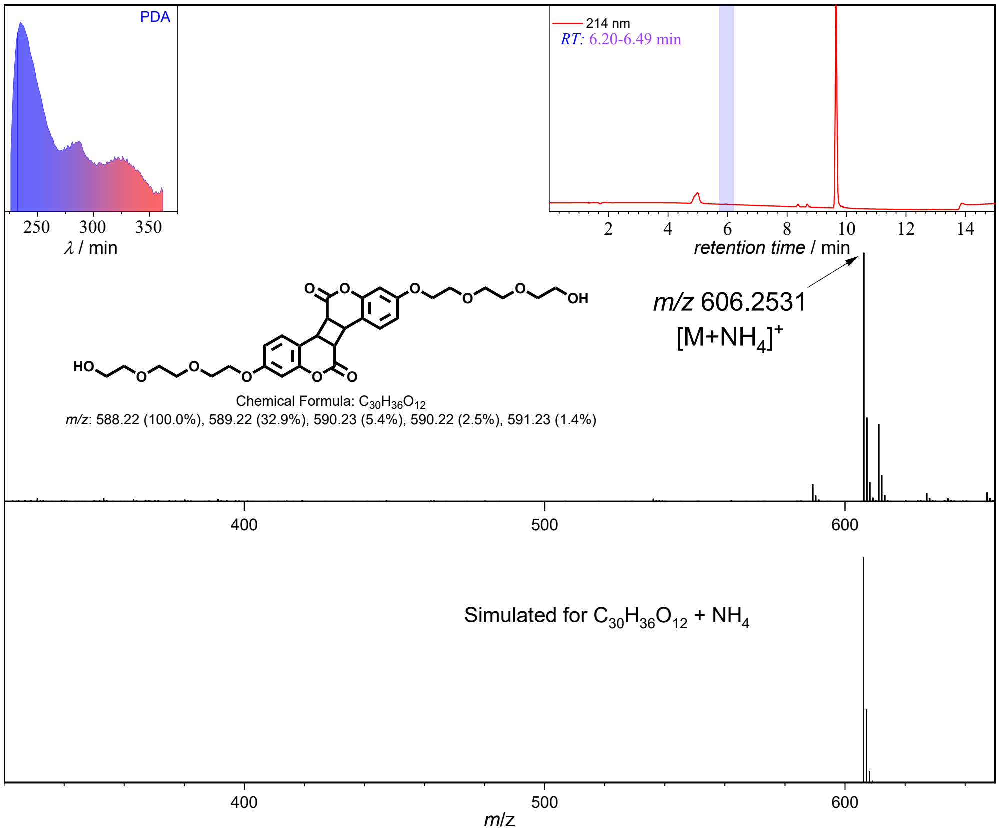

Figure S21 LC trace (214 nm detector wavelength) of CouTEG with styrene in ACN irradiated at 320 nm for 1 h, and corresponding mass spectrum for the homodimer at retention time 6.20-6.49 min and simulated mass spectrum (bottom) for the CouTEG homodimer.

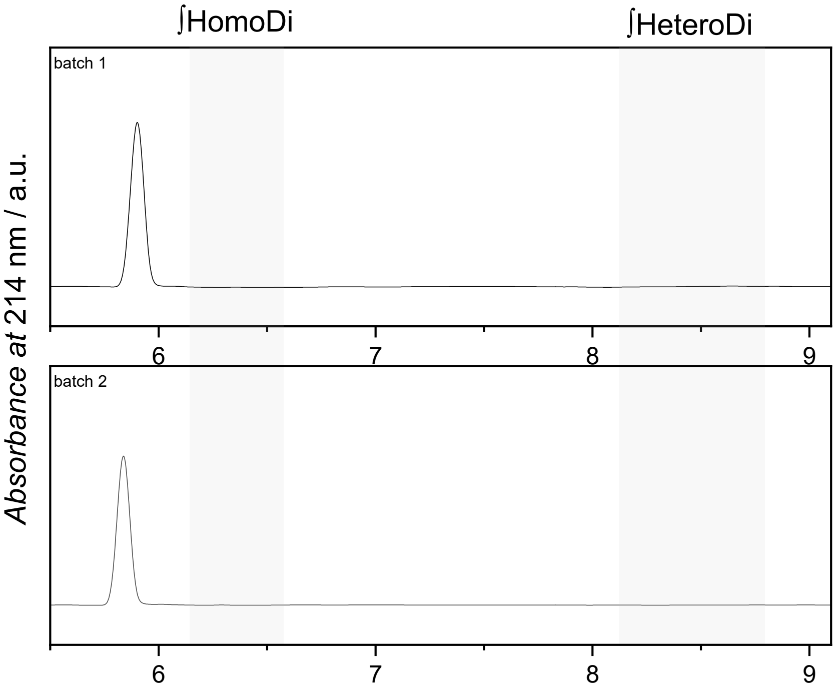

Figure S22 Representative LC traces (214 nm detector wavelength) of the [2+2] dimerization of CouTEG with styrene in ACN, before irradiation for batch 1 and batch 2. Elution volumes corresponding to the homodimer (6.20–6.49 min) and heterodimer (8.15–8.73 min) are highlighted.

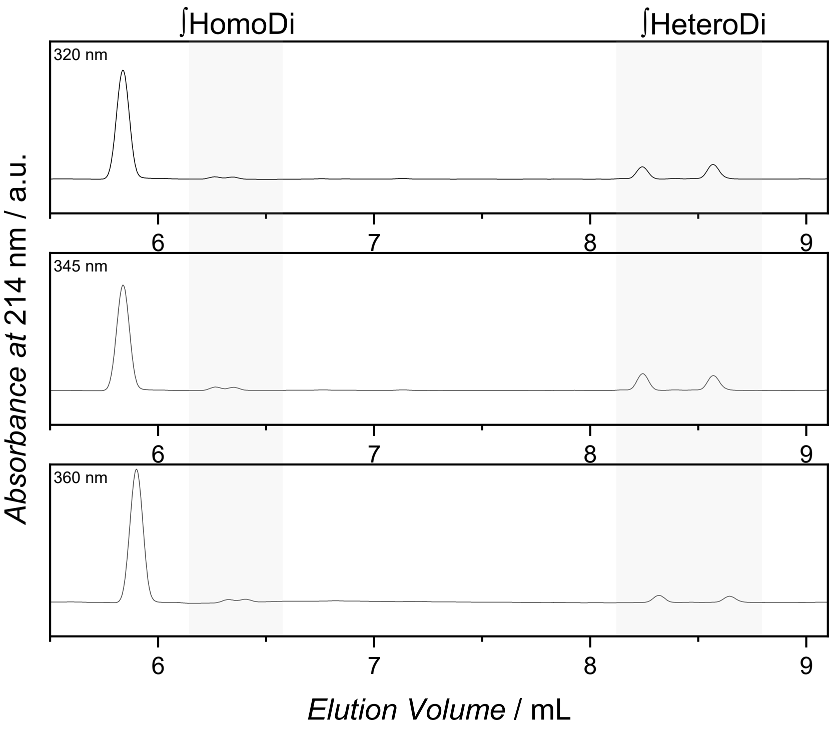

Figure S23 Representative LC traces (214 nm detector wavelength) of the [2+2] dimerization of CouTEG with styrene in ACN, irradiated at 320 nm, 345 nm or 360 nm for 1h. Elution volumes corresponding to the homodimer (6.20–6.49 min) and heterodimer (8.15–8.73 min) are highlighted.

2.4 Heterodimer Formation on SiO_2 Surfaces

2.4.1 Deposition of the Silane Functionalized coumarin (C4) on SiO_2 wafers

Before the surface functionalization, the SiO_2 substrates were cut into 1 × 1 cm^2 wafers and cleaned with a mixture of sulfuric acid and hydrogen peroxide (3:1) solution for 2 hours to remove organic contaminants. Subsequently, the wafers were thoroughly rinsed with distilled water and dried under a stream of nitrogen. The cleaned silicon wafers were then exposed to plasma treatment for 10 minutes to further activate the surface with hydroxyl groups. Next, the SiO_2 surfaces were covalently functionalized via hydrolysis of the silyl ether in dichloromethane (DCM) using a 2 mg·mL^{-1} solution overnight at 50 °C. After the functionalization wafers were rinsed in DCM and dried over N_2 flow and baked at 80 °C for 1 hour. Successful surface functionalization was confirmed by XPS and contact angle measurements.

2.4.2 Cou-StyPFA Heterodimer Formation on SiO_2 Wafers

Coumarin-functionalized SiO_2 wafers were immersed in a 2 mmol·L^{-1} *para*-styrene perfluoroalkyl ether (StyPFA) solution in acetonitrile. The wafers were placed vertically in a sealable vial using metal clips (**Figure S24 A**) to facilitate side LED irradiation. The vial was then sealed, degassed for 5 min, and irradiated with a 345 nm LED for 2 hours. The LED was positioned approximately 1 cm from the vial wall, with the beam directed onto the wafer surface. After irradiation, the wafers were rinsed with acetonitrile, followed by acetone, and dried under a nitrogen flow. Successful heterodimer formation was confirmed by XPS and contact angle measurements.

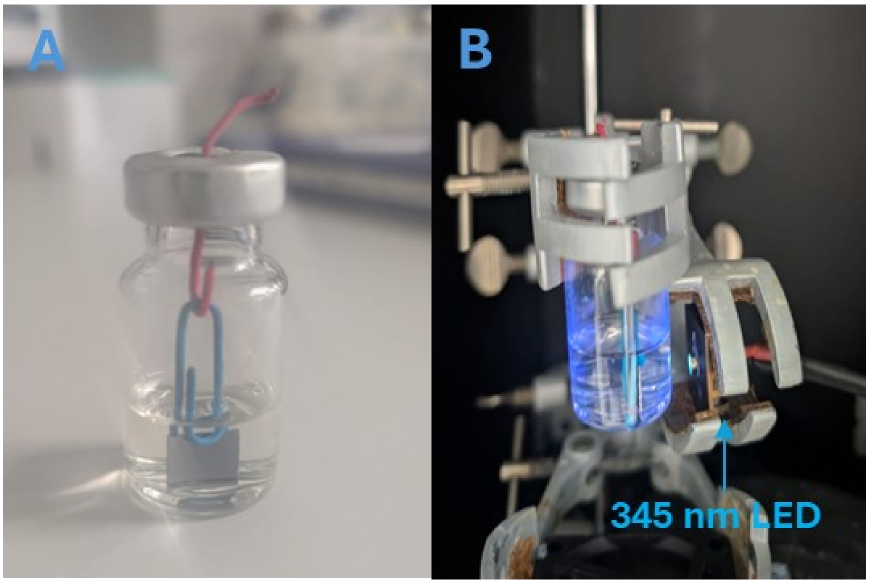

Figure S24 General setup for the photoreactions A sealed vial with SiO_2 wafers immersed in StyPFA solution (2 mmol·L^{-1}). B Irradiation setup with a λ_{max} = 325 nm as LED light source (emission spectrum of the employed LED can be found in section 1.2.8).

2.4.3 Photo-induced Cycloreversion on SiO_2 Wafers

Cycloreversion of the Cou-StyPFA heterodimer on the surface was carried out under UVC irradiation in a photoreactor described in section 1.2.7 (**Figure S25**). Eight lamps were installed for side irradiation. The internal chamber was ventilated to maintain ambient temperature during the entire experiment. After irradiation, the wafers were rinsed with acetonitrile, followed by acetone, and dried under a nitrogen flow. The samples were subsequently analysed by XPS and contact angle measurements.

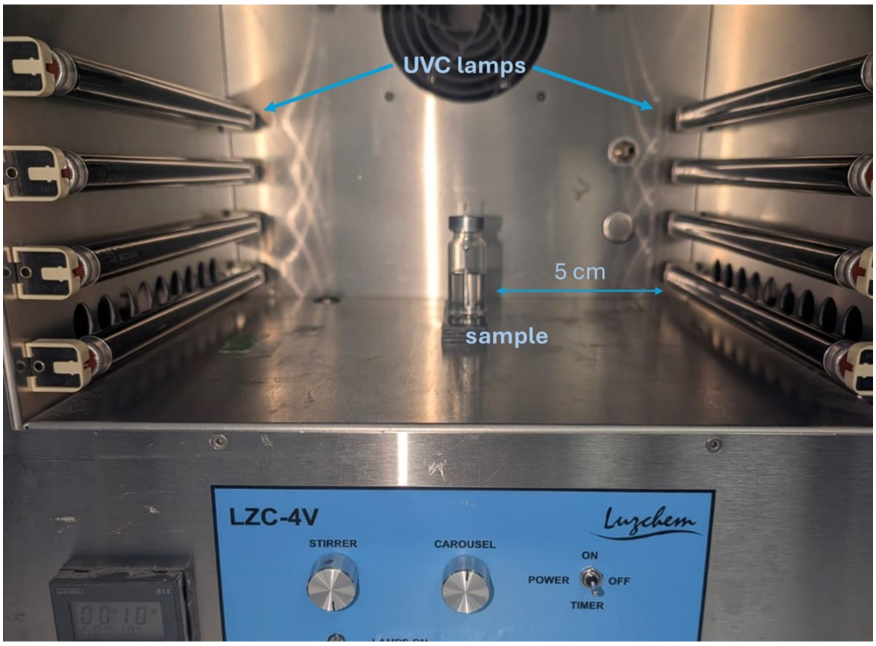

Figure S25 General setup for the photoinduced cycloreversion. The StyPFA functionalized wafer was in a sealed vial with ACN irradiated in a *Luzchem* LZC-4V photoreactor using LZC-UVC lamps, emitting at λ_{max} = 254 nm. Eight lamps were used, and the emission spectrum of the lamp can be found in section 1.2.7.

2.5 Effect of UVC Irradiation on Surface Recyclability

To evaluate whether the reduced recyclability originates from irradiation-induced photodamage, a control experiment was conducted on silicon wafers. The wafers were initially functionalized with a coumarin silane and characterized by XPS. The coumarin-modified surface was subsequently irradiated with UVC light (254 nm) for three times for 2 h in the absence of styrene, followed by XPS analysis. Subsequently, the same wafers were exposed to fluorinated styrene (StyPFA) under the same conditions as in section 2.4.2. The combined XPS results are shown in **Figure S26**. XPS revealed 0.4 % F on the surface. This result confirms that high-energy UVC irradiation induces irreversible photodamage of surface bound coumarin groups, contributing to the efficiency loss observed during recycling experiments.

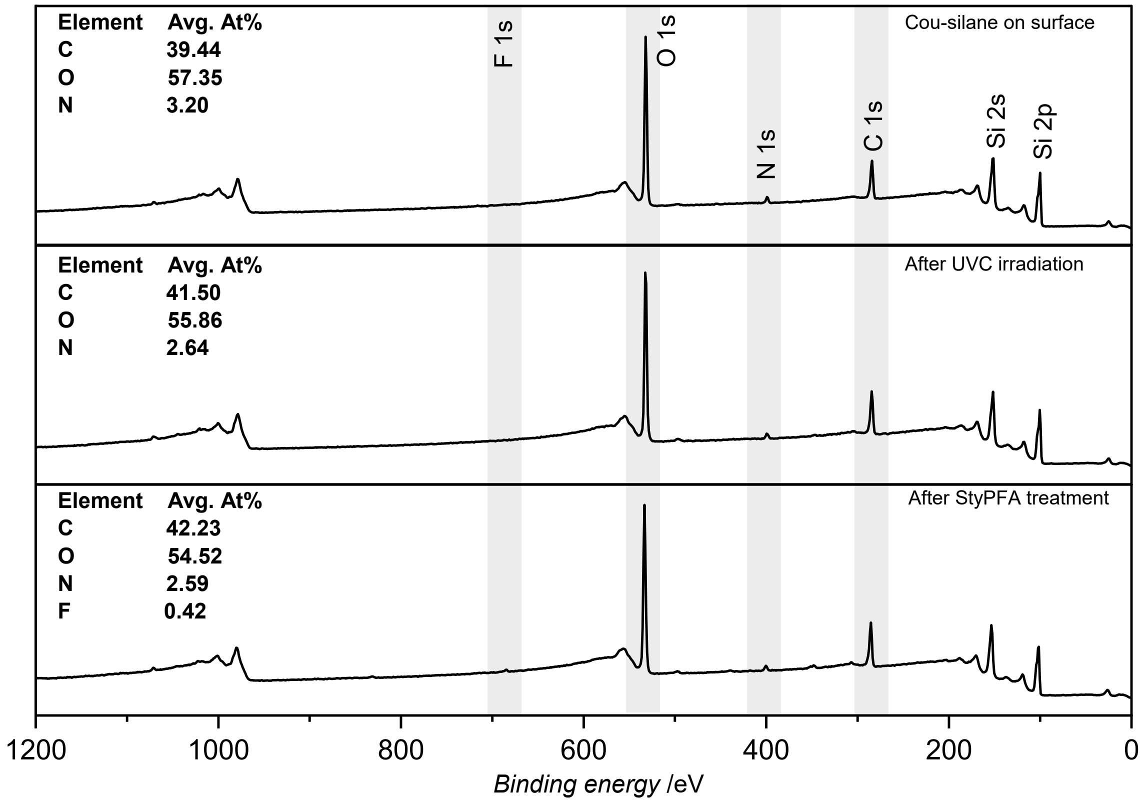

Figure S26 Top: XPS wide scan spectra of coumarin-silane functionalized SiO_2 substrate. Middle: The same SiO_2 substrate after UVC irradiation for 6 h. Bottom: Coumarin-StyPFA heterodimer formation on the same substrate after UVC irradiation.

2.6 Photoresin Preparation and Curing Procedure

The photoresin was formulated by mixing Clear Flex™ 50 part B with a 1:1 mixture of PEGDA and DEGDA containing 4 wt.% BAPO, and rotary mixing overnight. Subsequently, Clear Flex™ 50 Part A was added at a 1:2 ratio (Part A: Part B), resulting in an overall acrylate content of 30 wt.%. In the case of the addition of the acrylate-triethylene glycol functionalized coumarin (CouTEGA) to the photoresin, this was incorporated at the same stage as the BAPO at a 5 wt.% concentration based on the acrylate resin, resulting in a 1.5 wt.% overall concentration within the photoresin.

Prior to casting, the mixtures were stirred under light-protected conditions until homogeneous and subsequently degassed under vacuum to remove entrapped air bubbles.

After the samples were cast, the samples were initially subjected to photopolymerization by irradiation in the photoreactor as described in section 1.2.7 for 2 hours to initiate acrylate crosslinking, affording dimensional stability. 14 UVA lamps were installed for side and top irradiation. The secondary thermal curing step was then carried out with a gradient from RT to 100 °C, increasing the temperature with 0.1 °C min^{-1} and leaving the sample at 100 °C for 12 hours to activate the curing of the polyurethane

and achieve full network formation. The resulting cured photoresins were allowed to cool to room temperature before further characterization.

2.7 Cou-StyPFA Heterodimer Formation on Resin Surface

Initially, XPS analysis and contact angle measurements were performed on the freshly cured resin sample. For the control experiment, the resin sample was immersed in a StyPFA solution (2 mmol·L^{-1}) and kept in the dark for 2 hours. The sample was then rinsed thoroughly with fresh acetonitrile, dried under a nitrogen flow, and characterized by XPS. Subsequently, the same sample was immersed again in StyPFA solution (2 mmol·L^{-1}) and irradiated with UVA lamps inside a photoreactor (refer to **Figure S1** for the emission spectrum of the UVA lamp). Four lamps were used for side irradiation, and the irradiation was carried out for 2 hours. After the irradiation, the sample was rinsed thoroughly with fresh acetonitrile, dried under a nitrogen flow, and characterized by XPS and contact angle measurements.

2.8 Photochemical Control Experiment for the Resin without CouTEGA

Control resin samples without coumarin units (CouTEGA) were prepared and cured under identical conditions to those described in section 2.6. XPS analysis was performed on the cured resin samples. Subsequently, the resin was subjected to post-functionalization with the fluorinated styrene derivative (StyPFA) under the same conditions described in Section 2.7. After thorough washing, XPS analysis was conducted. As shown in **Figure S27**, XPS wide scan spectra (top) for the resin cured without CouTEGA before the StyPFA functionalization, the resin cured without CouTEGA after the StyPFA treatment (middle) and resin cured with CouTEGA after the StyPFA functionalization (bottom), only a very low fluorine content (0.56 at.%) was detected, indicating negligible incorporation of StyPFA in the absence of coumarin units. This result confirms that efficient heterodimer formation occurs only in the presence of coumarin moieties within the resin.

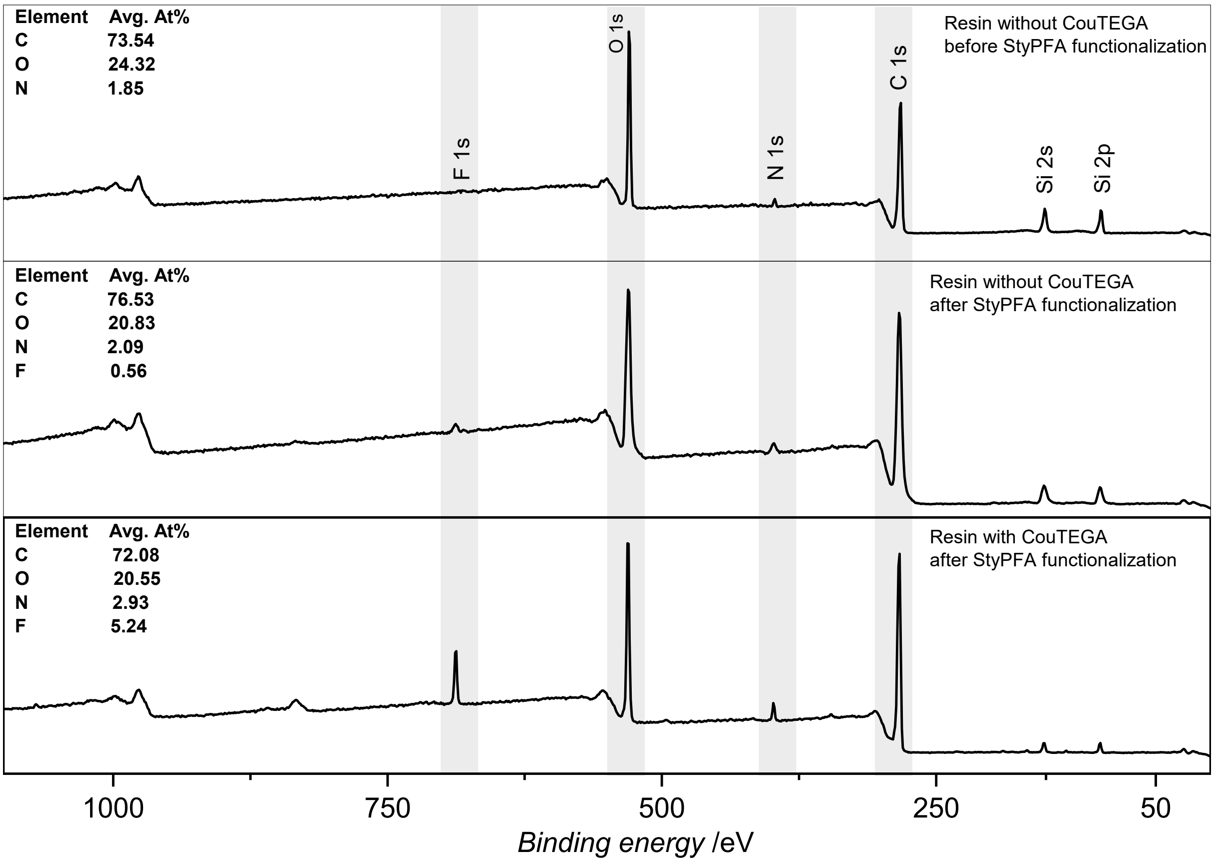

Figure S27 Top:XPS wide scan spectra for the resin cured without CouTEGA before the StyPFA functionalization. Middle: The resin cured without CouTEGA after the StyPFA treatment. Bottom: Resin cured with CouTEGA after the StyPFA functionalization

3 Action Plot Procedures

3.1 General Photochemical Action Plot Procedure[1]

All laser experiments were conducted using the apparatus shown in **Figure S28.** The light source was an Opotek Opolette HE 355 LD OPO, producing approx. 5 ns duration pulses at a repetition rate of 20 Hz. The output beam was initially passed through a beam expander (50 mm and 100 mm lens combination) to ensure it is sufficiently large to uniformly irradiate the entire sample volume. The beam is subsequently directed upwards using a UV Fused silica right angle prism. Finally, the beam enters the sample from below, suspended in an aluminium block. The laser energy deposited into the sample was measured before and after experiments using a Coherent EnergyMax thermopile sensor (J-25MB-LE) to determine if any significant power fluctuations occurred during irradiation.

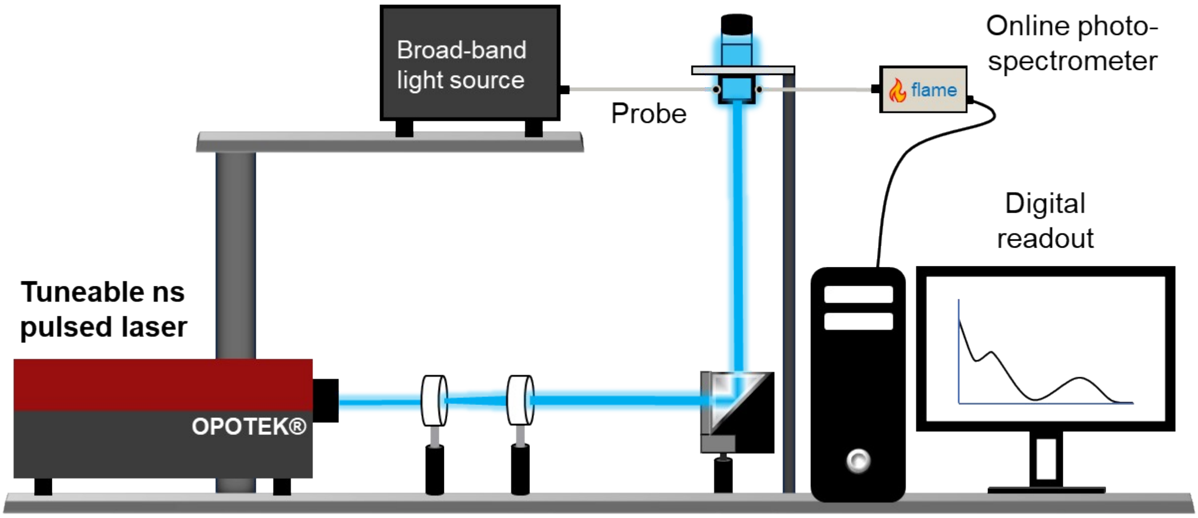

Figure S28 Schematic diagram of the photochemical action plot experiment used in the current study. Note that the shown on-line UV/Vis monitoring component was not employed.

For laser measurements, all samples were prepared in a 0.8 mL glass crimp vials (internal diameter 6.2 mm) capped with a crimp seal. The wavelength dependent glass transmittance, essential for quantitative measurements, is presented in **Figure S29**. Transmittance of the bottom of the glass vials used in the current study.

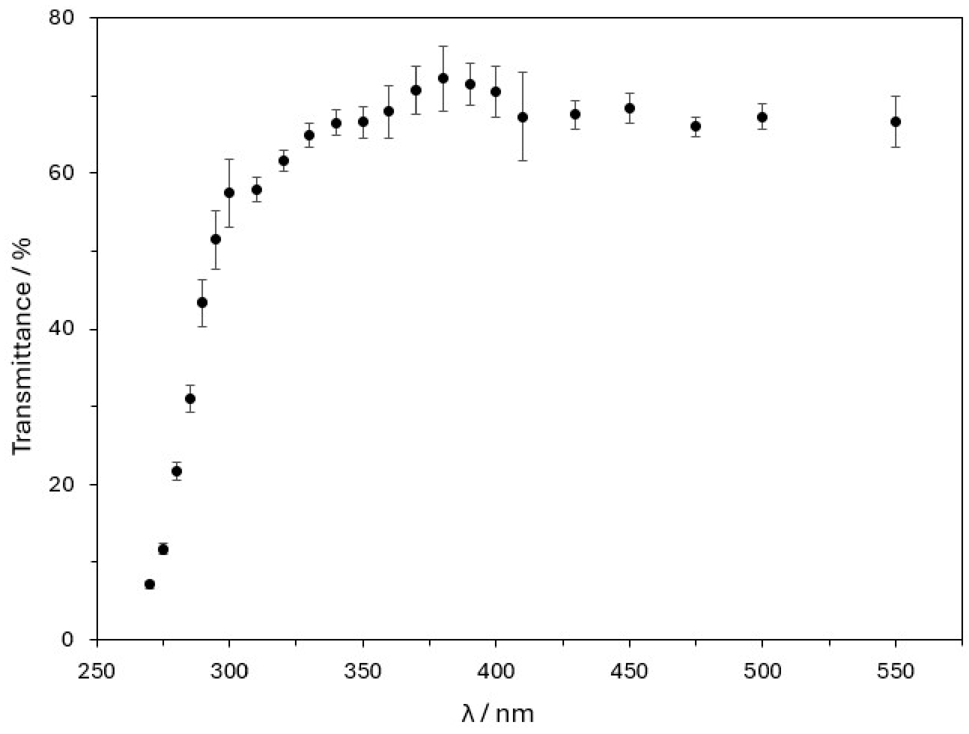

Figure S29 Transmittance of the bottom of the glass vials used in the current study.

Precise photons numbers were determined from the laser pulse energy using the following relation:

$$N_p = \frac{E_{pulse}\lambda f_{rep} t}{hc\,[T_\lambda/100]} \quad (1)$$

Where E_{pulse} is the measured pulse energy on the top of the laser vial, λ is the wavelength of the incident radiation, f_{rep} is the laser repetition rate, t is the irradiation time, h is Planck's constant, c is the speed of light and T_λ is the wavelength dependent glass transmission presented in Equation (1). Once an initial measurement is completed and the photon number is known, the required energies at other wavelengths can be found by rearranging the above equation to give E_{pulse}, Equation (2).

$$E_{pulse} = \frac{N_p hc[T_\lambda/100]}{\lambda f_{rep} t} \quad (2)$$

3.2 [2+2] Dimerization Action Plot of 7-Hydroxycoumarin-Styrene

3.2.1 Heterodimer Kinetics and Action Plot

To monitor the [2+2] heterodimer formation via photochemical action plots, all samples were prepared at the concentration of 0.9 mmol·L^{-1} 7HCou and 9.1 mmol·L^{-1} of styrene in ACN-d_3 (sample volume close to 0.8 mL). Subsequently, the samples were individually placed into the sample holder and irradiated at ambient temperature. First, the formation of heterodimer was monitored as a function of irradiation time (at the maximum absorbance of 7HCou (320 nm), thereby varying the number of photons delivered (**Table S1** and **Figure S30**). The kinetic plot allowed us to determine the linear regime of the heterodimerization to ensure all further experiments are conducted where the number of delivered photons correlates linearly with the conversion.

For the action plot, the irradiation time (3600 s) was kept constant, but the laser power was varied to deposit an identical number of photons at each wavelength (approx. 4.0×10^{19} photons). The irradiation was carried out by the tuneable laser system described in section 3.1 (Opotek Opolette HE 355 LD OPO laser with a 20 Hz pulse repetition rate). After the irradiation, ^{1}H NMR spectroscopic analysis was conducted to obtain the heterodimer yield and LC-MS analysis for further confirmation of heterodimer formation (**Table S2** and sections 3.3 and 2.3).

The molar extinction coefficients of 7HCou and styrene were determined by dividing their respective absorbance spectra by their concentration. Molar extinction coefficients and heterodimerization quantum yields were overlayed to obtain the heterodimerization action plot, as shown in Figure 1 in the main paper.

Table S1 Heterodimerization kinetics of the reaction of 7-hydroxycoumarin with styrene. LSE_{start} and LSE_{end} (*LSE* = Laser Energy) are the energies of the laser before and after the irradiation, irradiation time in minutes and heterodimer conversion determined by ^{1}H NMR spectroscopy (spectra and analysis provided in section 4.1 and 4.2).

Wavelength / nm	LSE_{start} / µJ	LSE_{end} / µJ	$t_{irridation}$ / min	*Conversion* / %
320	606 ± 15	607 ± 14	10	-
320	617 ± 14	606 ± 15	20	0.37
320	613 ± 15	617 ± 14	30	0.47
320	711 ± 21	694 ± 20	60	0.81
320	663 ± 14	615 ± 13	90	1.22
320	615 ± 13	613 ± 15	120	1.55

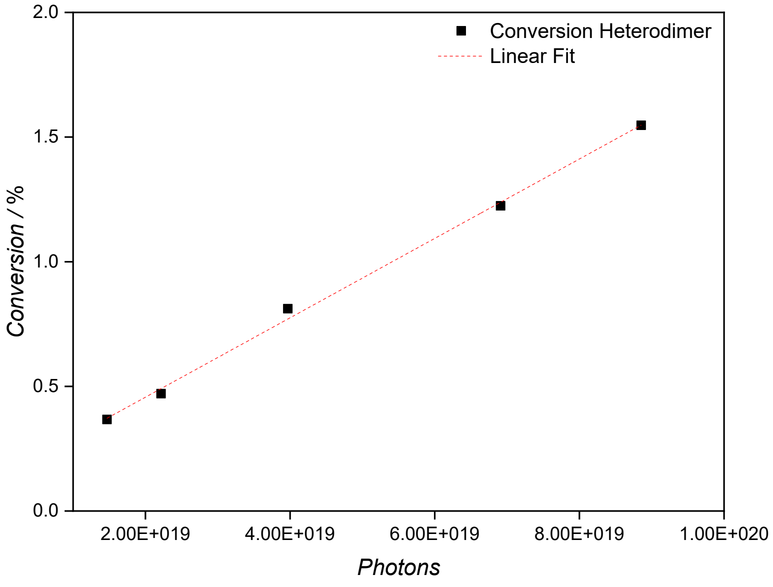

Figure S30 Heterodimerization photon kinetic plot of 7-hydroxy coumarin with styrene, demonstrating that within the delivered number of photons the conversion to heterodimer is linear.

Table S2 Heterodimerization overview of the wavelength dependence of 7HCou with styrene. LSE_{start} and LSE_{end} (LSE = Laser Energy) are the energies of the laser before and after the irradiation, heterodimer conversion determined by ^{1}H NMR spectroscopy (spectra and analysis provided in section 3.3) and calculated quantum yields (ϕ) (as explained in below) with the standard deviations (σ) for the conversion and the ± error for the quantum yields (ϕ_{Error}).

Wavelength/nm	LSE_{Avg}/µJ	*# photons*	*Conversion/%*	$Conv_{avg}$/%	σ / %	ϕ	ϕ_{Error}
310	714 ± 20	4.67 ×10^{19}	0.63	0.63	0.03	0.59×10^{-4}	0.03×10^{-4}
310	713 ± 17	4.66×10^{-4}	0.61				
310	715 ± 16	4.68 ×10^{-4}	0.68				
310	703 ± 21	4.60×10^{-4}	0.62				
320	695 ± 11	5.00×10^{-4}	0.64	0.66	0.07	0.59×10^{-4}	0.06×10^{-4}
320	676 ± 14	4.87×10^{-4}	0.61				
320	645 ± 14	4.64×10^{-4}	0.74				
330	606 ± 14	4.67×10^{-4}	0.96	0.94	0.03	0.87×10^{-4}	0.03×10^{-4}
330	604 ± 15	4.65×10^{-4}	0.95				
330	606 ± 15	4.67×10^{-4}	0.89				

330	608 ± 16	4.68×10^{-4}	0.96				
345	519 ± 13	4.31×10^{-4}	1.02	1.07	0.05	0.97×10^{-4}	0.04×10^{-4}
345	572 ± 17	4.75×10^{-4}	1.10				
345	566 ± 18	4.70×10^{-4}	1.10				
355	538 ± 18	4.65×10^{-4}	0.80	0.82	0.02	0.83×10^{-4}	0.02×10^{-4}
355	534 ± 19	4.61×10^{-4}	0.83				
360	520 ± 12	4.57×10^{-4}	0.33	0.35	0.06	0.55×10^{-4}	0.06×10^{-4}
360	516 ± 15	4.54×10^{-4}	0.40				
360	542 ± 17	4.77×10^{-4}	0.40				
360	535 ± 17	4.70×10^{-4}	0.28				
370	511 ± 18	4.64×10^{-4}	0.07	0.09	0.02	0.21×10^{-4}	0.02×10^{-4}
370	497 ± 15	4.51×10^{-4}	0.11				
370	517 ± 18	4.70×10^{-4}	0.09				

3.2.2 Quantum Yield (*Φ*) Calculations[2]

The quantum yields Φ for the dimerization was calculated for each averaged wavelength data point of the action plot measurements according to the following formula (3):

$$\Phi = \frac{\rho\, c\, V\, N_A}{N_p(1 - 10^{-A(\lambda)})} \tag{3}$$

where ρ is the conversion of the heterodimer, N_p is the number of photons delivered, $A(\lambda)$ is the absorbance at a given wavelength, c is the initial concentration of 7-hydroxy coumarin molecules (0.9 mM total), V is the volume of sample (800 µL), and N_A is Avogadro's number. As the absorbance changes over time, due to changes in chromophore concentration, one should typically calculate the absorbance change over time. Since we only reached a maximum conversion of 1.07%, we decided to simplify the calculations by using the initial absorbance only. The quantum yield calculations are only accurate when the absorbance of the formed dimer product is negligible, which is the case (refer to **Figure S31**).

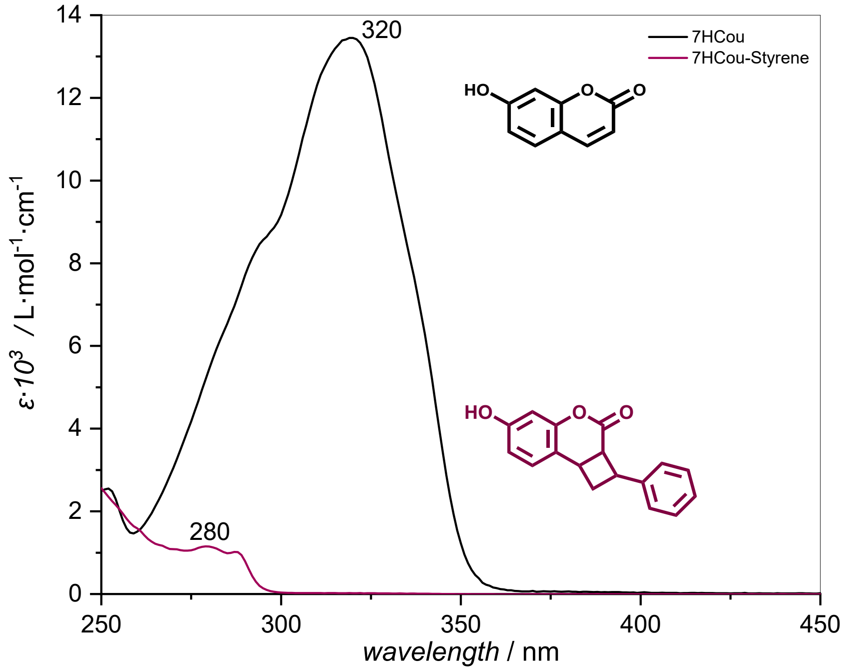

Figure S31. Overlay of the extinction spectra of 7HCou and the 7HCou-Styrne heterodimer.

3.2.3 Functionalized 7-Hydroxycoumarin Extinction Spectrum

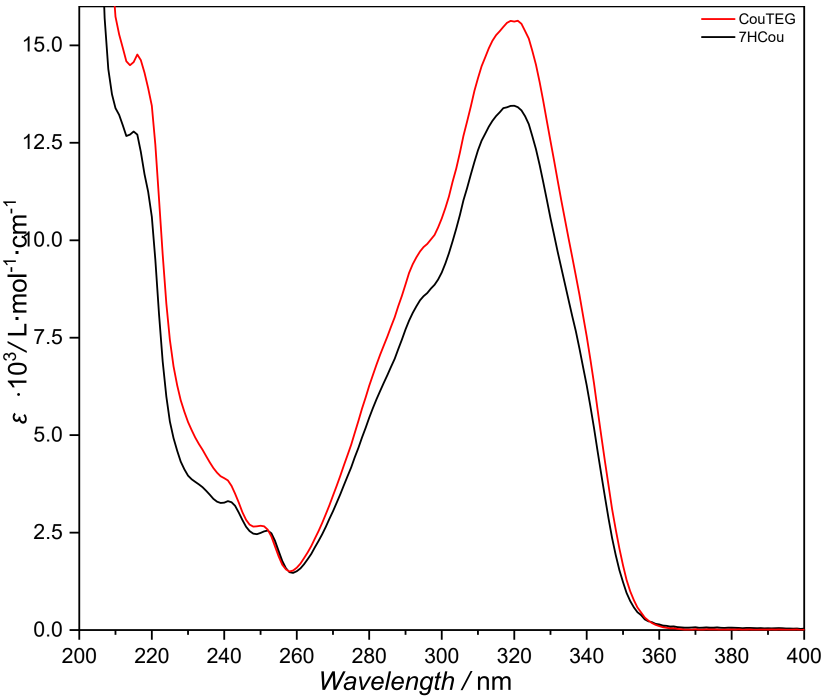

Figure S32 Overlay of the extinction spectra of 7HCou and CouTEG.

3.3 NMR Analysis for the Photochemical Action Plot Measurements

The formation of the coumarin-based homo- and heterodimers was assessed by ^{1}H NMR spectroscopy, according to their characteristic NMR resonances. The formation of mixtures of head-to-head (HH) and

head-to-tail (HT) regioisomeric products[3, 4] complicates the spectra. **Figure S33A** shows the calculated (ChemDraw 25.0.2.14) ^{1}H NMR spectra of all dimer species hypothesized to be formed during the [2+2] cycloaddition reaction between 7-hydroxycoumarin and styrene. As expected, the cyclobutene region demonstrates multiple resonances linked to each specific hypothesized regioisomer (**Figure S33B-E**).

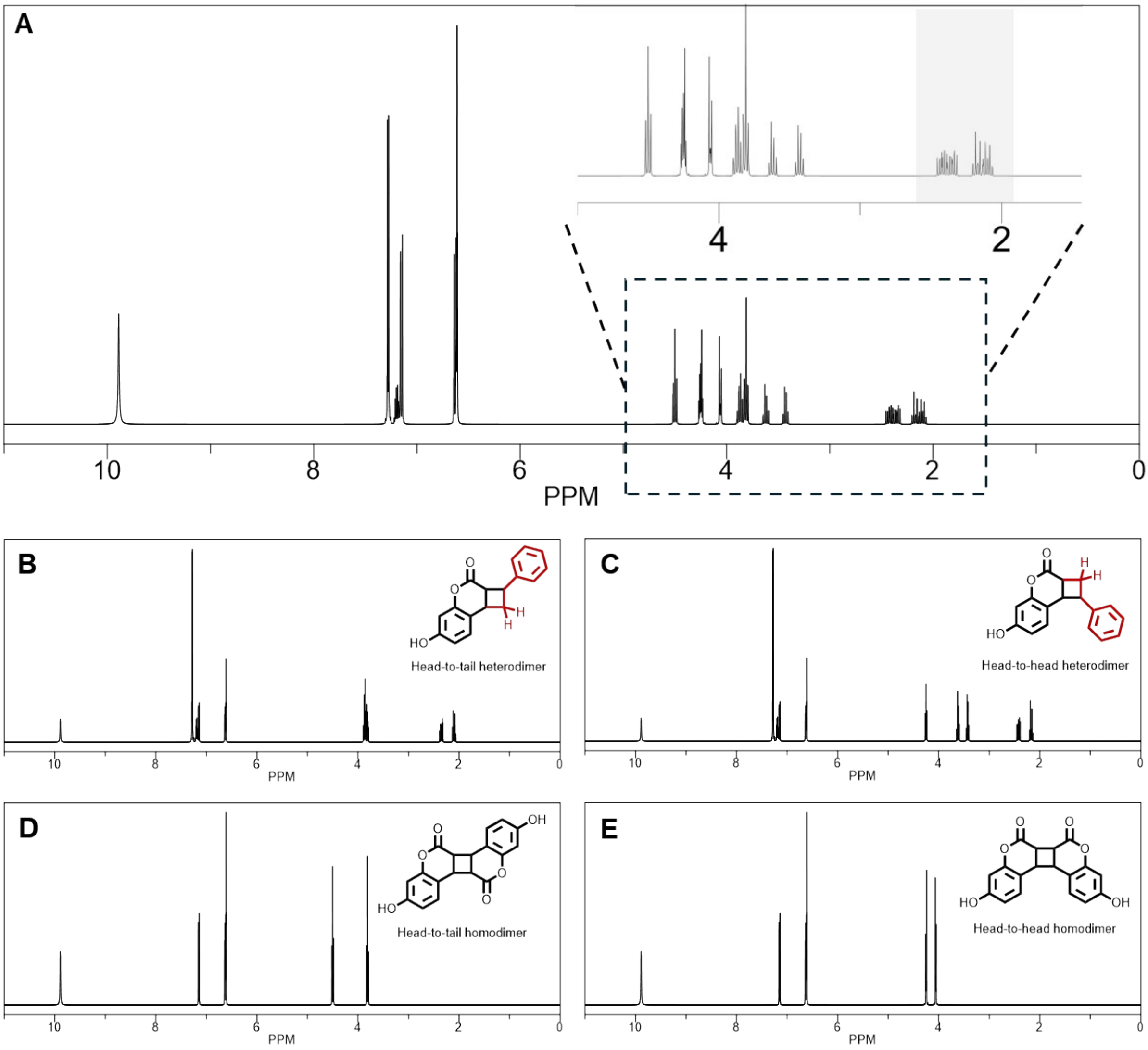

Figure S33 Estimated ChemDraw ^{1}H-NMR spectrum of hetero- and homodimer regioisomers in DMSO. A Mixture of all regioisomers. B Head-to-tail (ht) C Head-to-head (hh) heterodimer spectra and D Head-to-tail (ht) E head-to-head (hh) homodimer spectra.

To confirm the formation of the hypothesized regioisomers of the homo- and heterodimer and to support quantification of the 1D ^{1}H spectra, a COSY (**Figure S34**) NMR spectrum was recorded on a dimerization sample after 2 hours of laser irradiation at 320 nm (8.86×10^{19} photons delivered). The analysis was complicated by the very low concentration of dimers with respect to unreacted 7-hydroxycoumarin and styrene.

The homo- and heterodimers were distinguished according to differences in the cyclobutane region (δ 1.5–5.0 ppm). In CD_3CN, the cyclobutane proton resonances occur in three distinct regions: 2.6 – 2.9 ppm, 3.4 – 3.8 ppm and 4.0 – 4.4 ppm. The resonances in the 2.6 – 2.9 ppm region are exclusively assigned to the diastereotopic CH_2 protons of the cyclobutane ring (derived from styrene) in the heterodimer isomers, according to spectrum prediction and structure elucidation of isolated coumarin-styrene heterodimers (**Figure S35**). In the COSY spectrum, geminal *J* coupling correlations are observed between resonances in this region as expected for the CH_2. Resonances in the 3.4 – 3.8 ppm region can all be correlated to the geminal CH_2 signals, confirming that these can be assigned to the heterodimers.

In the 4.0 – 4.4 ppm region, the stronger resonances (4.03 and 4.14 ppm) correlate with heterodimer resonances in the lower ppm regions, although there is overlap with weaker resonances. These less-intense signals only show correlation with other signals (e.g. 4.09, 4.23 and 4.29 ppm) in this region, and not with the previously mentioned heterodimer resonances. Consequently, we assign these to the homodimer isomers. Again, these conclusions are consistent with spectral predictions, previous reports[5] of coumarin homodimers and our own observations of the homodimer.

As resonances in the 2.6 - 2.9 ppm range can be exclusively assigned to the cyclobutene protons derived from styrene in the heterodimer – and not the homodimer – they can be used to calculate the heterodimer yield for the photochemical action plot. Theoretically, the yield to the homodimer can then be determined based on the remaining integral of the cyclobutane region. In practice, however, the overall conversion of both hetero and specifically homodimer is low, resulting in a poor signal-to-noise for the ^1H NMR spectra even after several hours of data acquisition. This makes the reliable determination of the homodimer yield for the purpose of a photochemical action plots highly challenging, and we refrain from using these data.

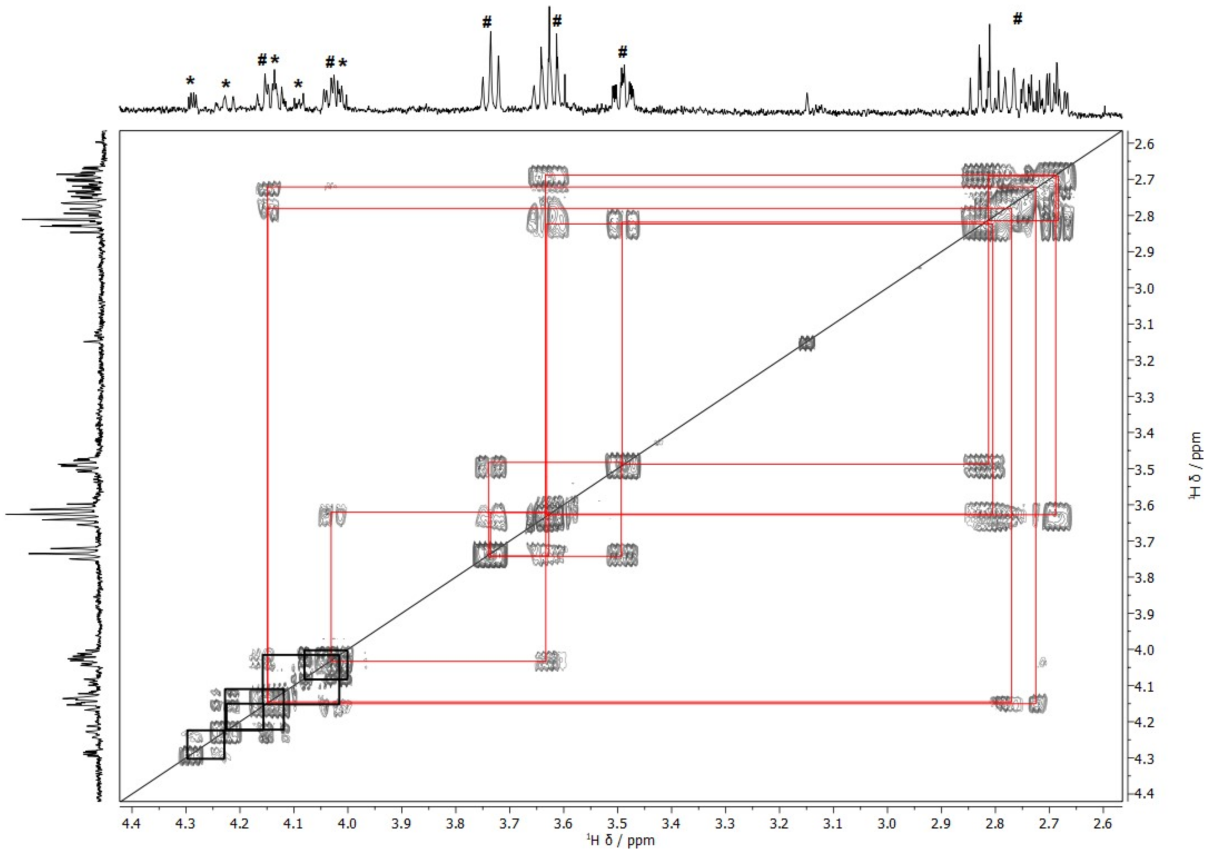

Figure S34 Cyclobutane region of the COSY spectrum of a 7-HCou/styrene dimerization after 2 h of laser irradiation at 320 nm (8.86 × 10^{19} photons). Resonances marked with # and correlations in red correspond to the heterodimer isomers. Conversely, resonances marked with * and correlations in black correspond to the 7-hydroxycoumarin homodimer isomers. The spectrum was recorded at 600 MHz in CD_3CN at 298 K.

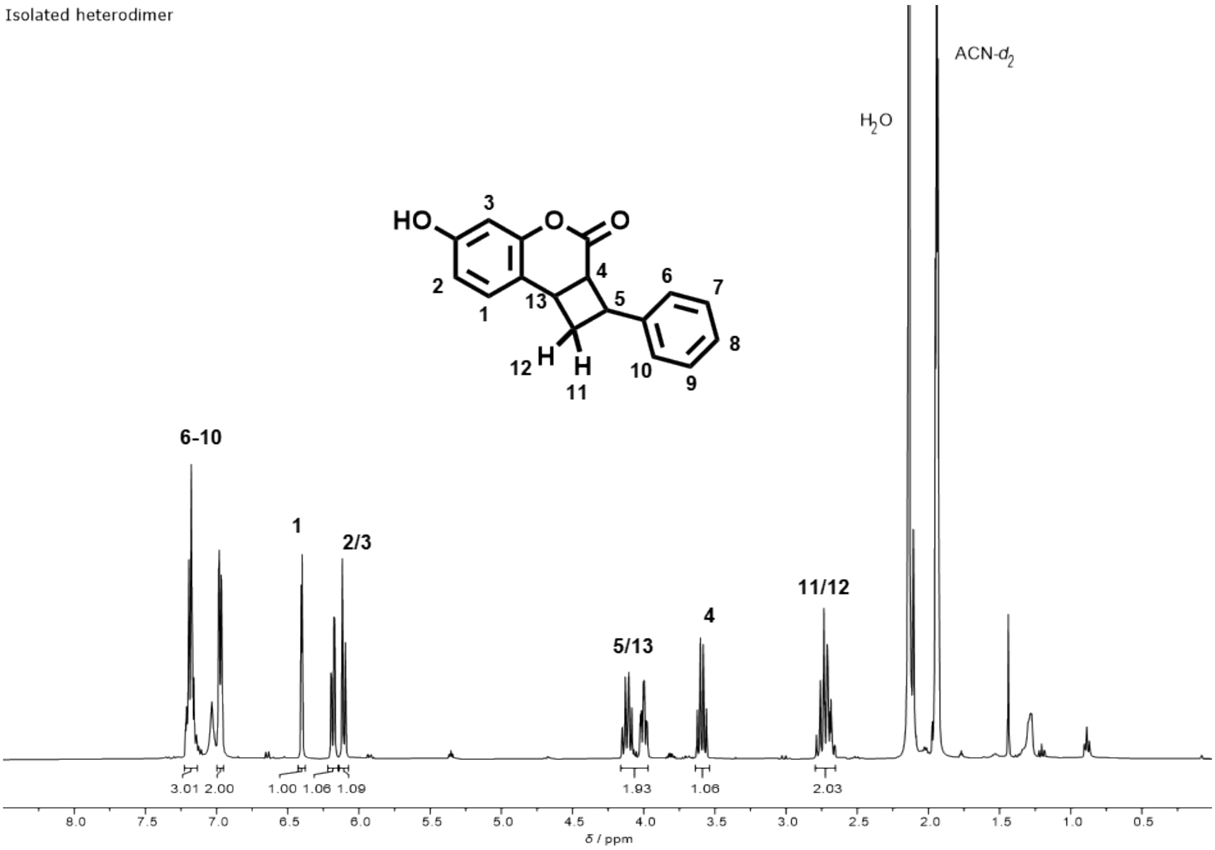

Figure S35 ^{1}H NMR spectrum of the isolated 7HCou-Styrene heterodimer.

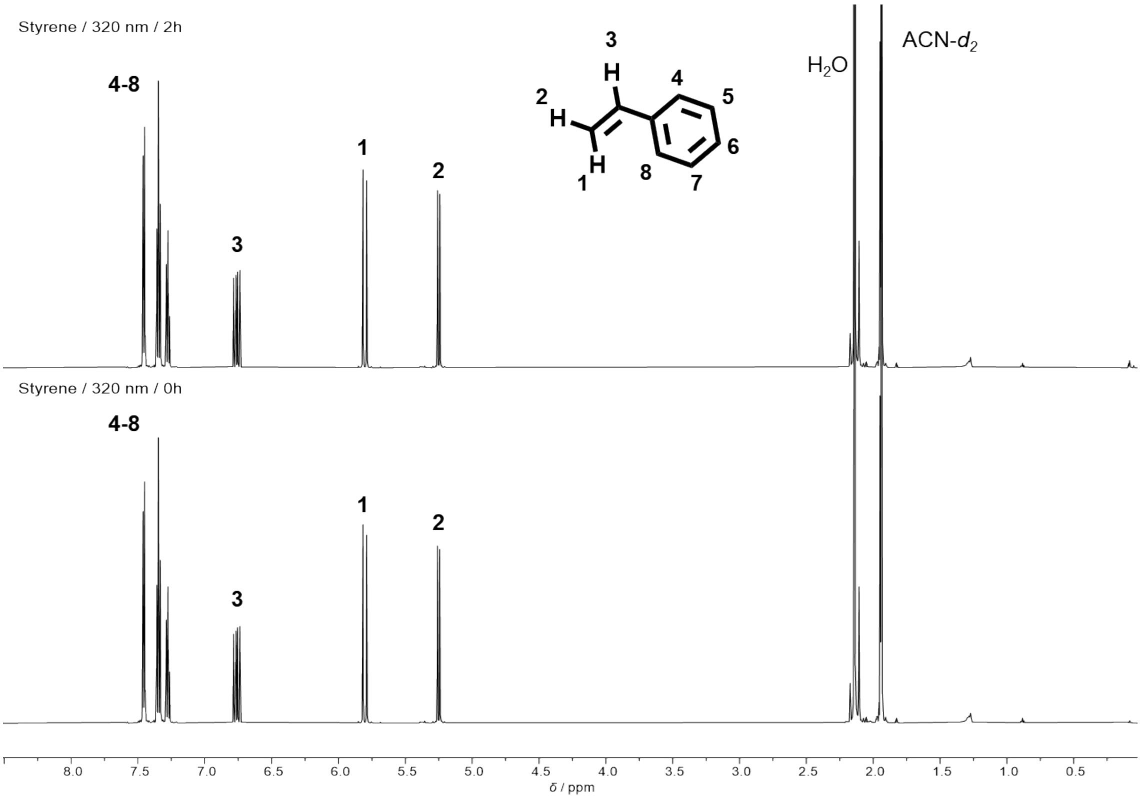

Figure S36 ^{1}H NMR spectrum of styrene in ACN-d_3 before and after irradiation for 2 h at 320 nm.

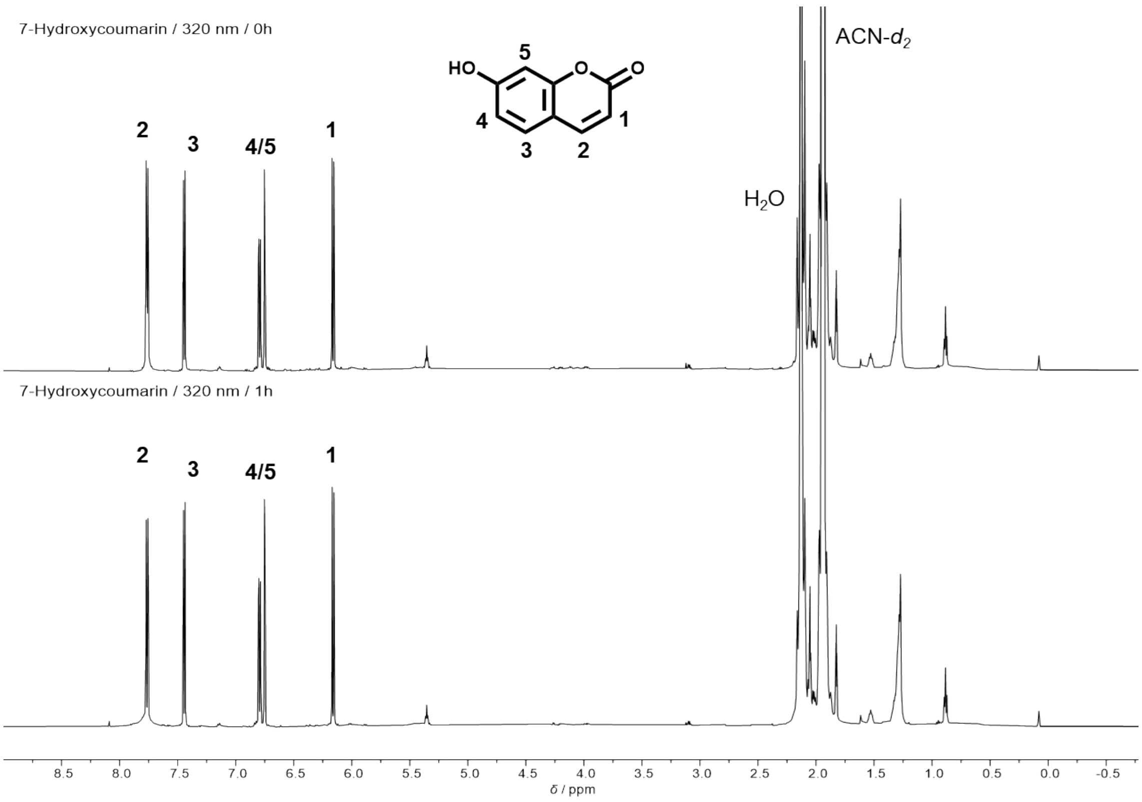

Figure S37 ^{1}H NMR spectrum of 7-hydroxycoumarin in ACN-d_3 before and after irradiation for 2 h at 320 nm.

4 Supplementary Data

4.1 NMR Spectra for the Photochemical Action Plot Measurements

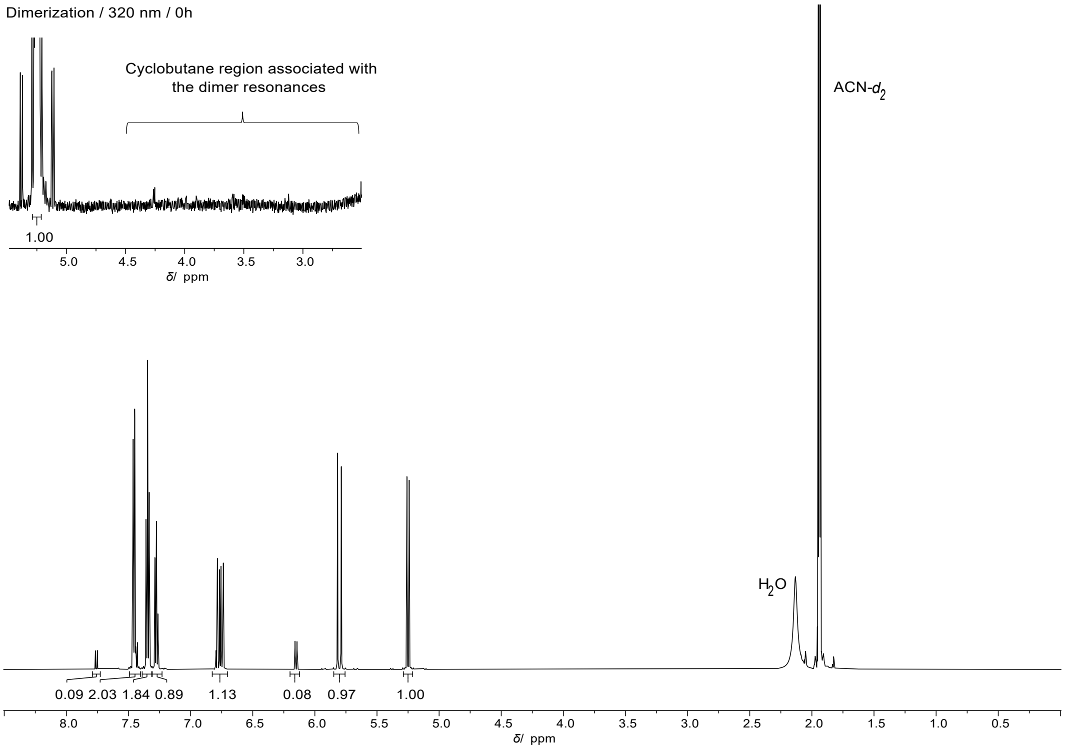

Figure S38 ^{1}H NMR spectrum of 7-hydroxycoumarin with styrene in ACN-d_3 before irradiation. The zoomed insert visualizes the cyclobutane region.

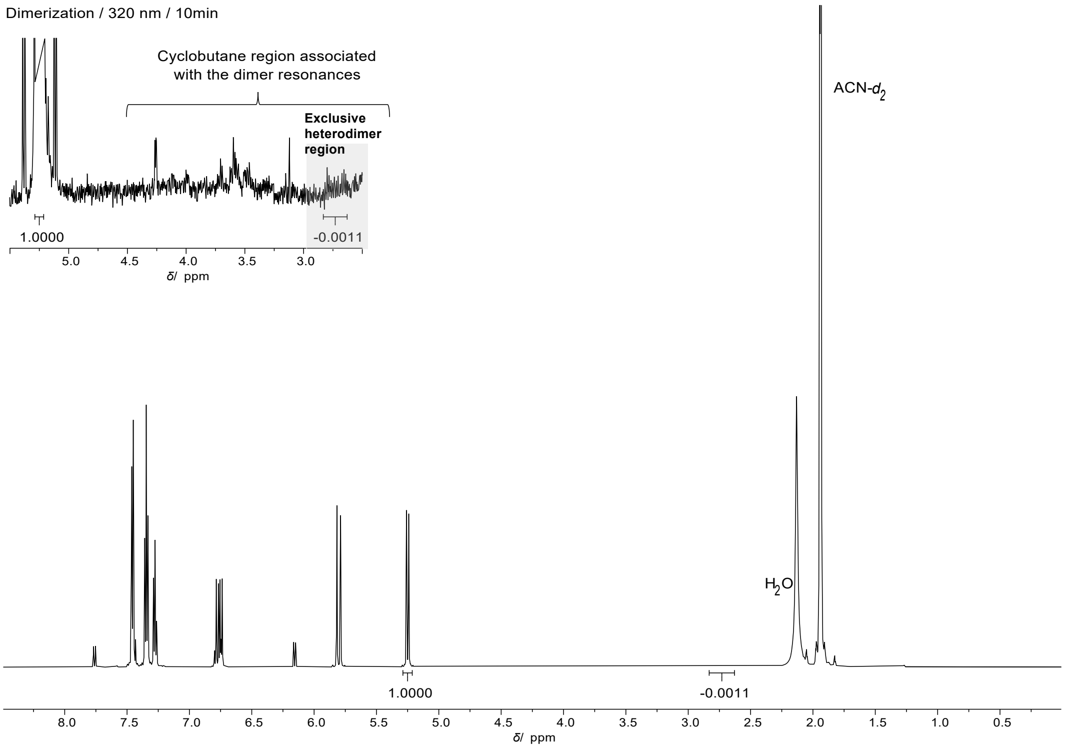

Figure S39 ^{1}H NMR spectrum of 7-hydroxycoumarin with styrene in ACN-d_3 irradiated at 320 nm for 10 min. The zoomed insert visualizes the cyclobutane region and highlights the integrated area used to determine the heterodimer conversion.

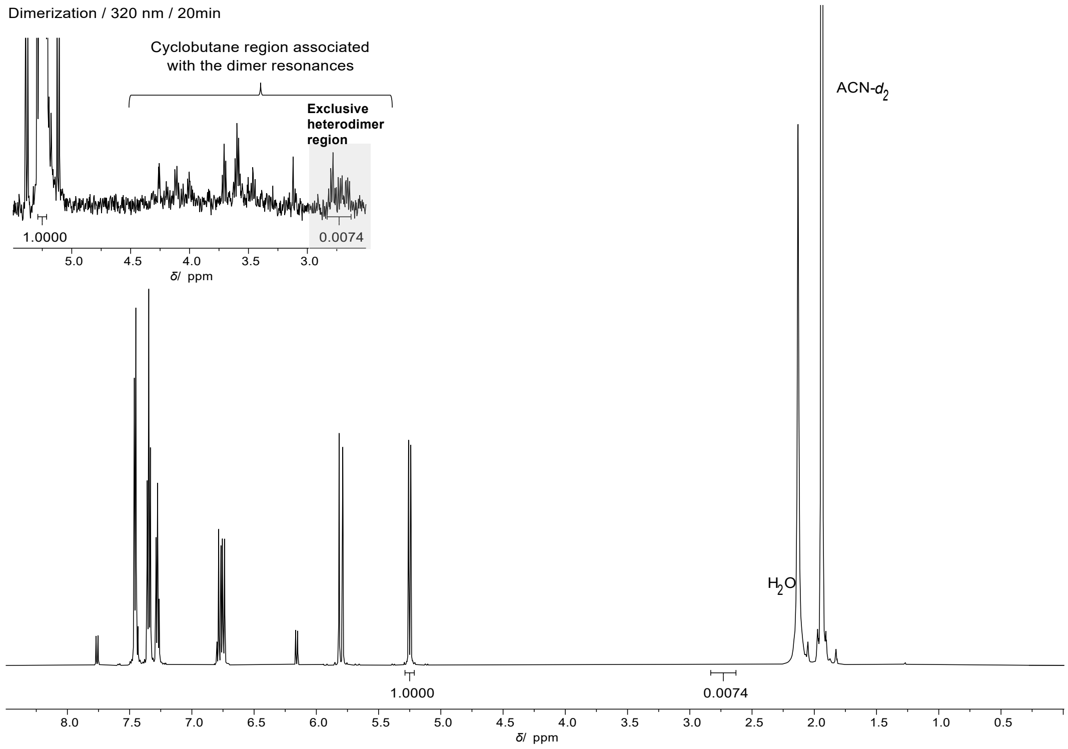

Figure S40 ^{1}H NMR spectrum of 7-hydroxycoumarin with styrene in ACN-d_3 irradiated at 320 nm for 20 min. The zoomed insert visualizes the cyclobutane region and highlights the integrated area used to determine the heterodimer conversion.

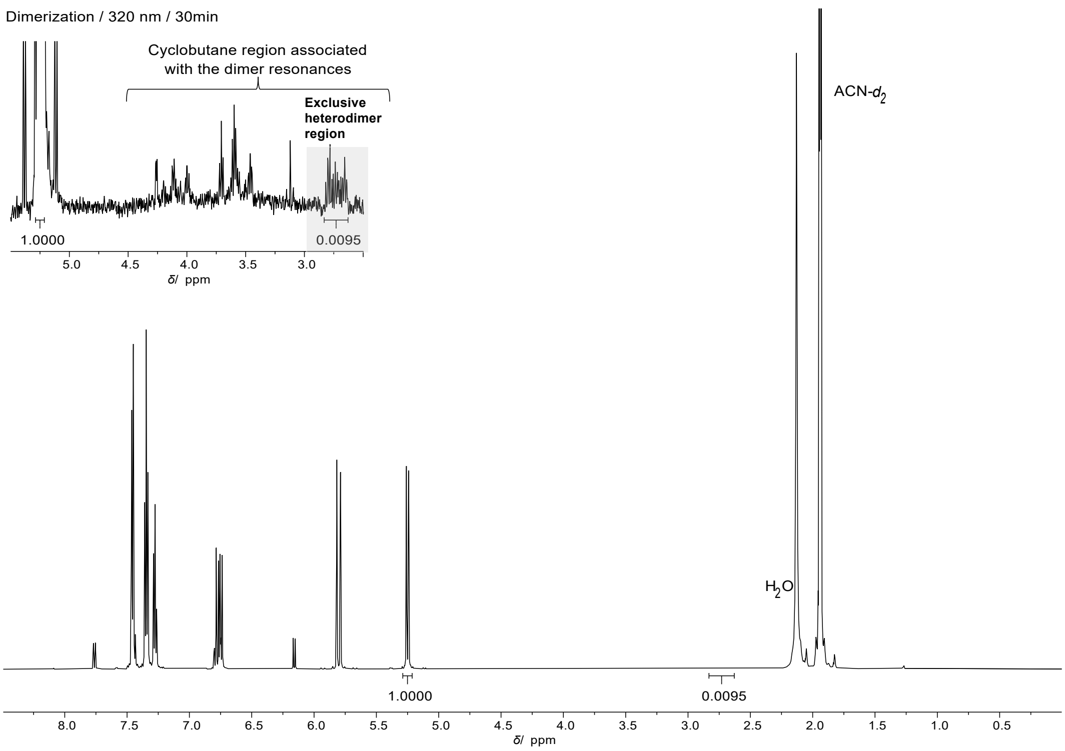

Figure S41 ^{1}H NMR spectrum of 7-hydroxycoumarin with styrene in ACN-d_3 irradiated at 320 nm for 30 min. The zoomed insert visualizes the cyclobutane region and highlights the integrated area used to determine the heterodimer conversion.

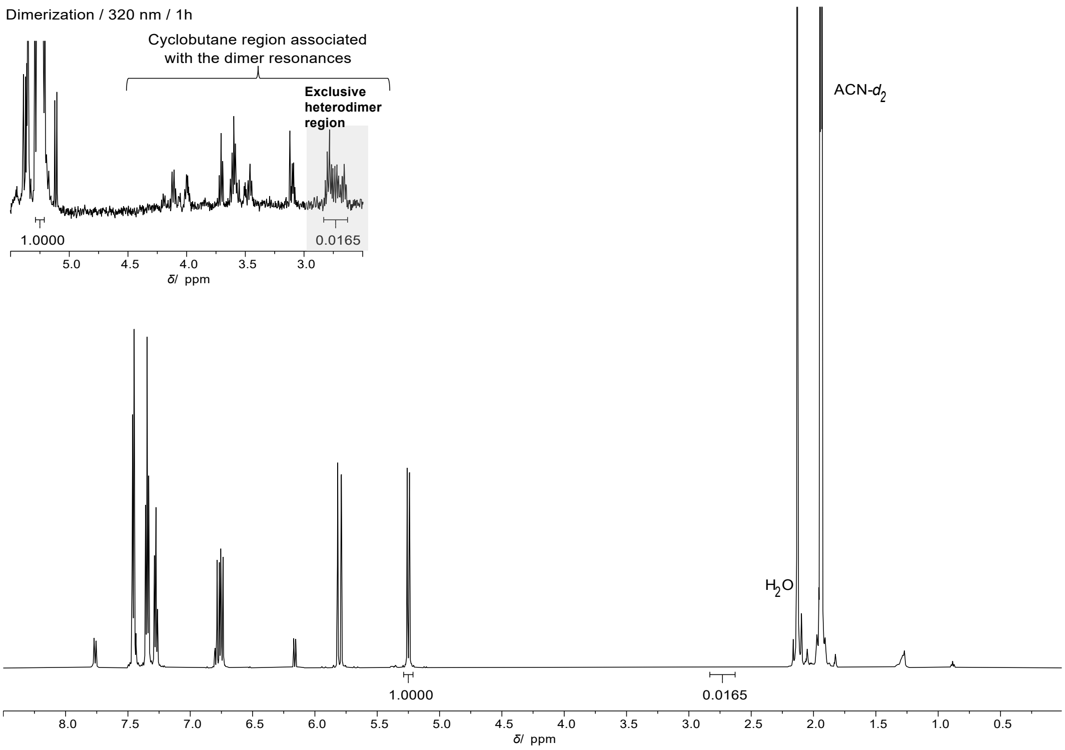

Figure S42 ^{1}H-NMR spectrum of 7-hydroxy coumarin with styrene in ACN-d_3 irradiated at 320 nm for 1 h. The zoomed insert visualizes the cyclobutane region and highlights the integrated area used to determine the heterodimer conversion.

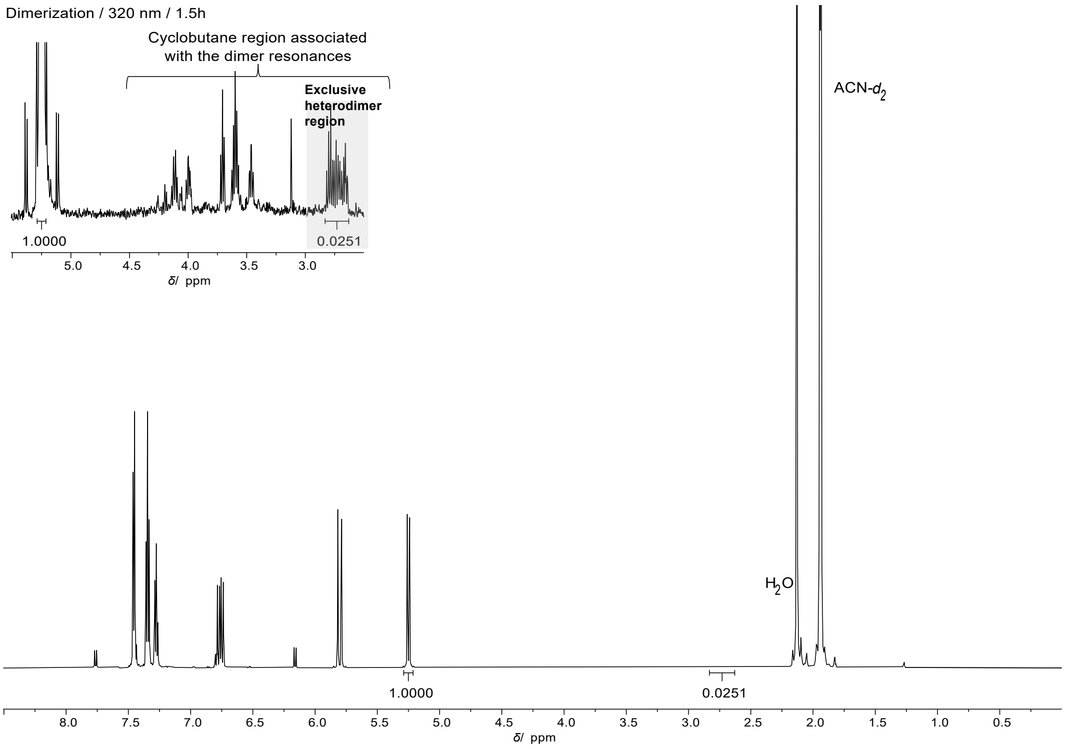

Figure S43 ^{1}H-NMR spectrum of 7-hydroxy coumarin with styrene in ACN-d_3 irradiated at 320 nm for 1.5 h. The zoomed insert visualizes the cyclobutane region and highlights the integrated area used to determine the heterodimer conversion.

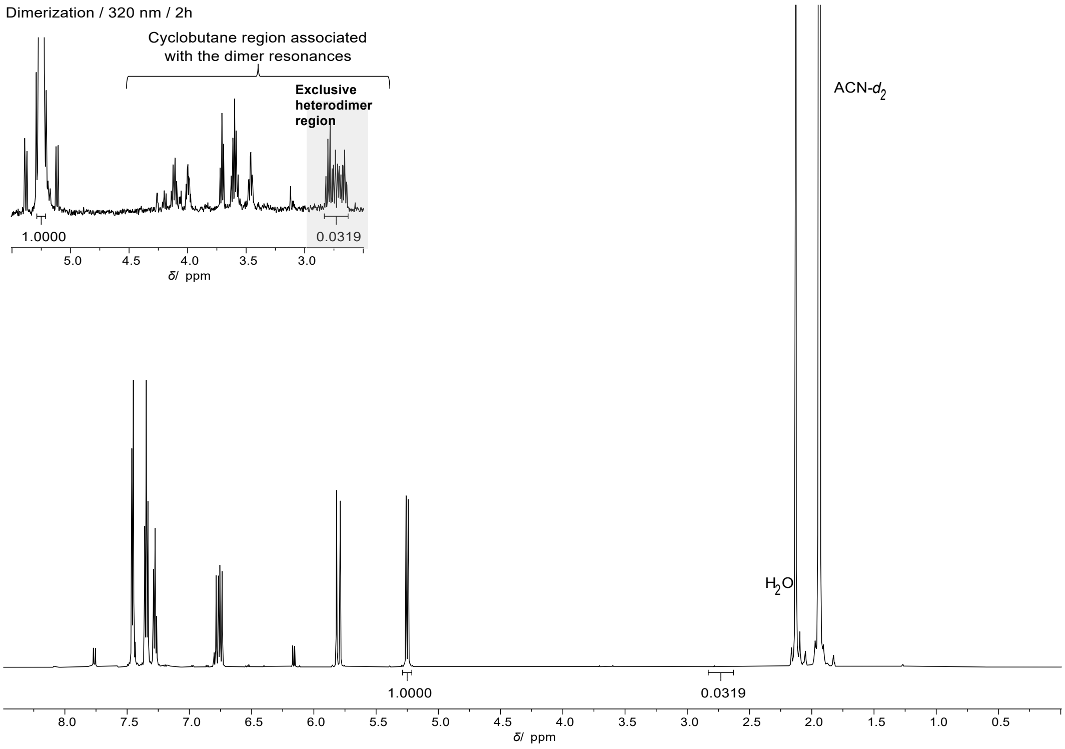

Figure S44 ^{1}H-NMR spectrum of 7-hydroxy coumarin with styrene in ACN-d_3 irradiated at 320 nm for 2 h. The zoomed insert visualizes the cyclobutane region and highlights the integrated area used to determine the heterodimer conversion.

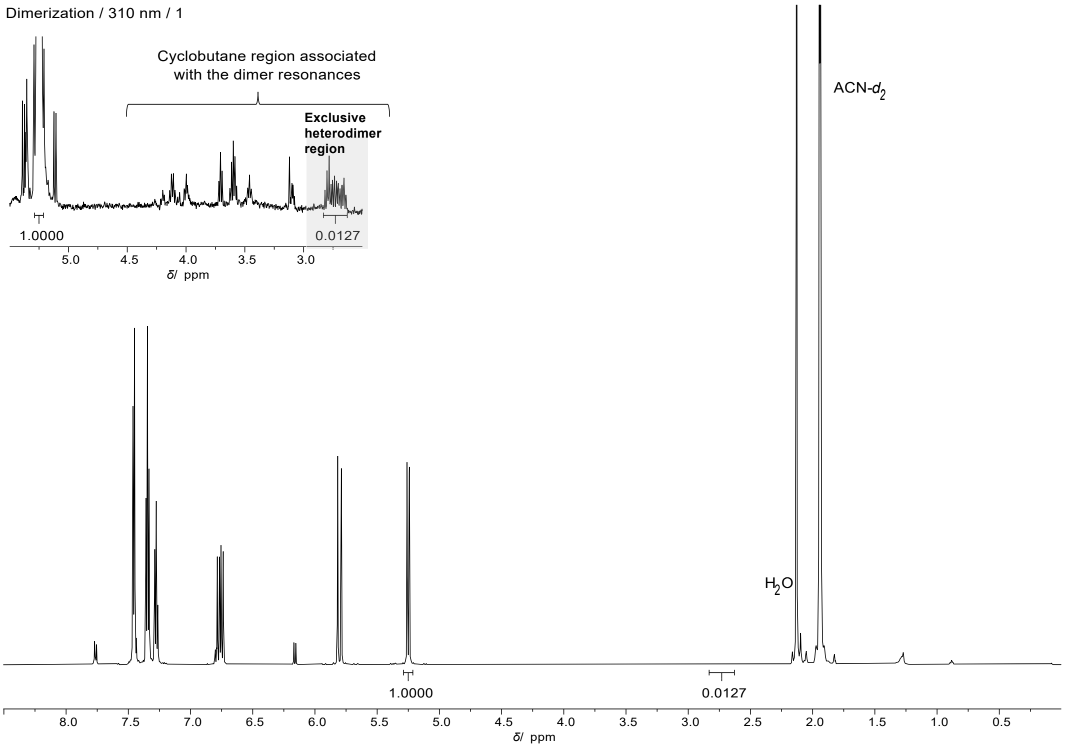

Figure S45 ^{1}H-NMR spectrum of 7-hydroxycoumarin with styrene in ACN-d_3 irradiated at 310 nm for 1 h, replicate 1. The zoomed insert visualizes the cyclobutane region and highlights the integrated area used to determine the heterodimer conversion.

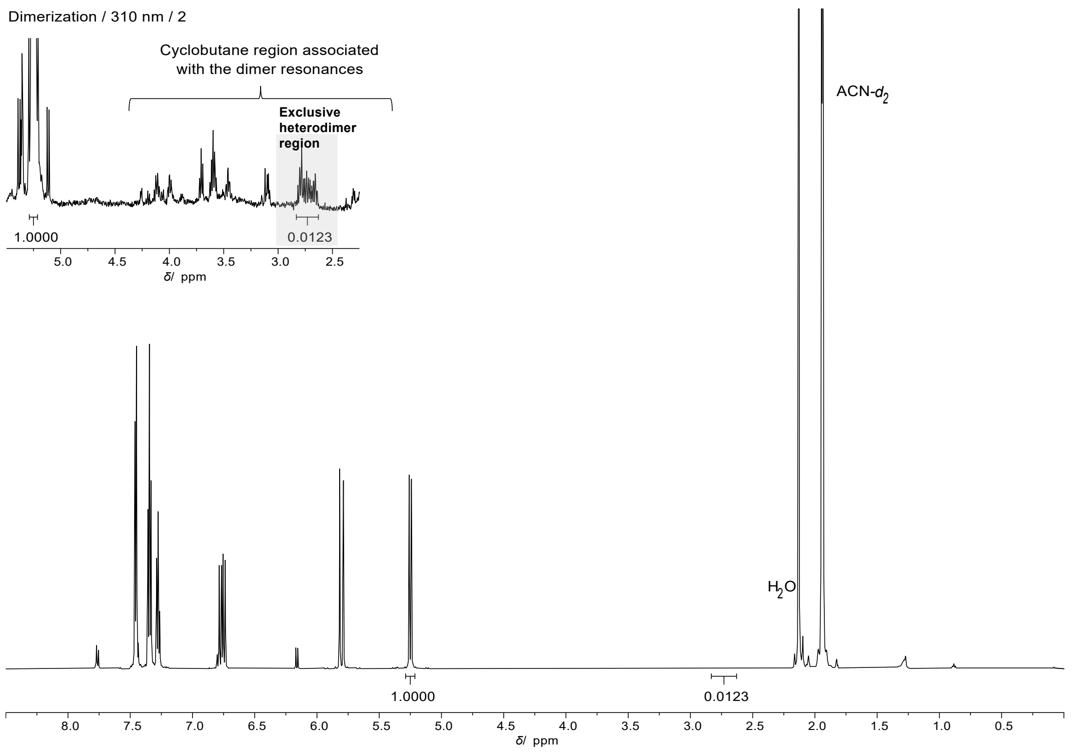

Figure S46 ^{1}H-NMR spectrum of 7-hydroxy coumarin with styrene in ACN-d_3 irradiated at 310 nm for 1 h, replicate 2. The zoomed insert visualizes the cyclobutane region and highlights the integrated area used to determine the heterodimer conversion.

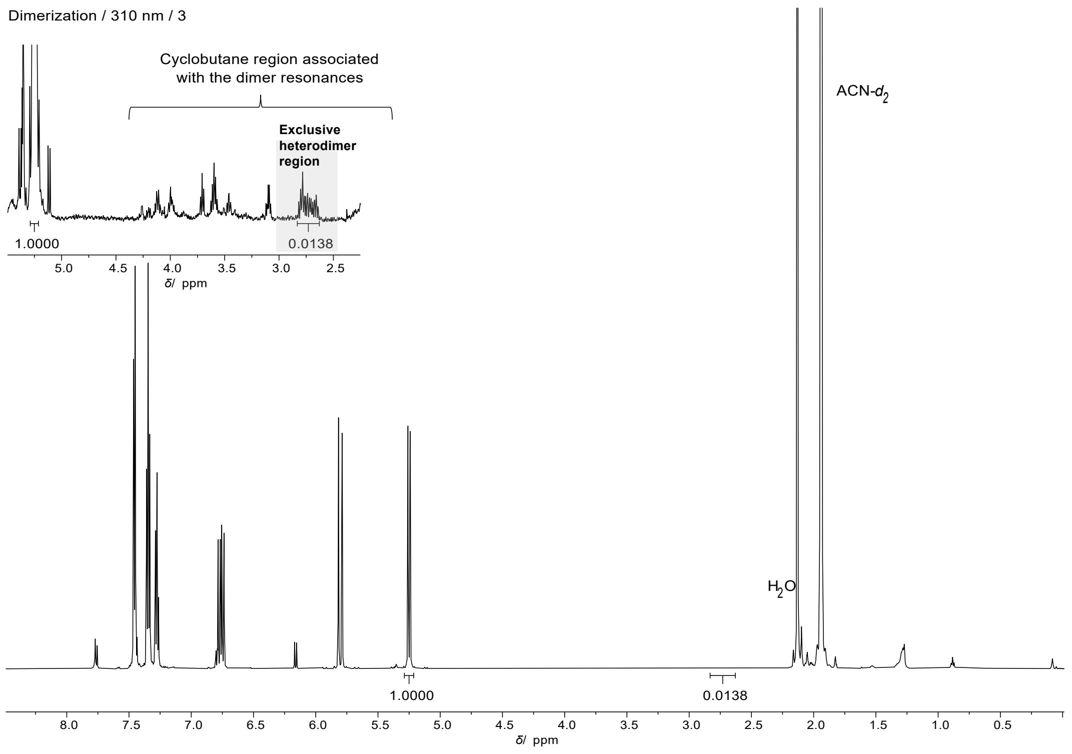

Figure S47 ^{1}H-NMR spectrum of 7-hydroxycoumarin with styrene in ACN-d_3 irradiated at 310 nm for 1 h, replicate 3. The zoomed insert visualizes the cyclobutane region and highlights the integrated area used to determine the heterodimer conversion.

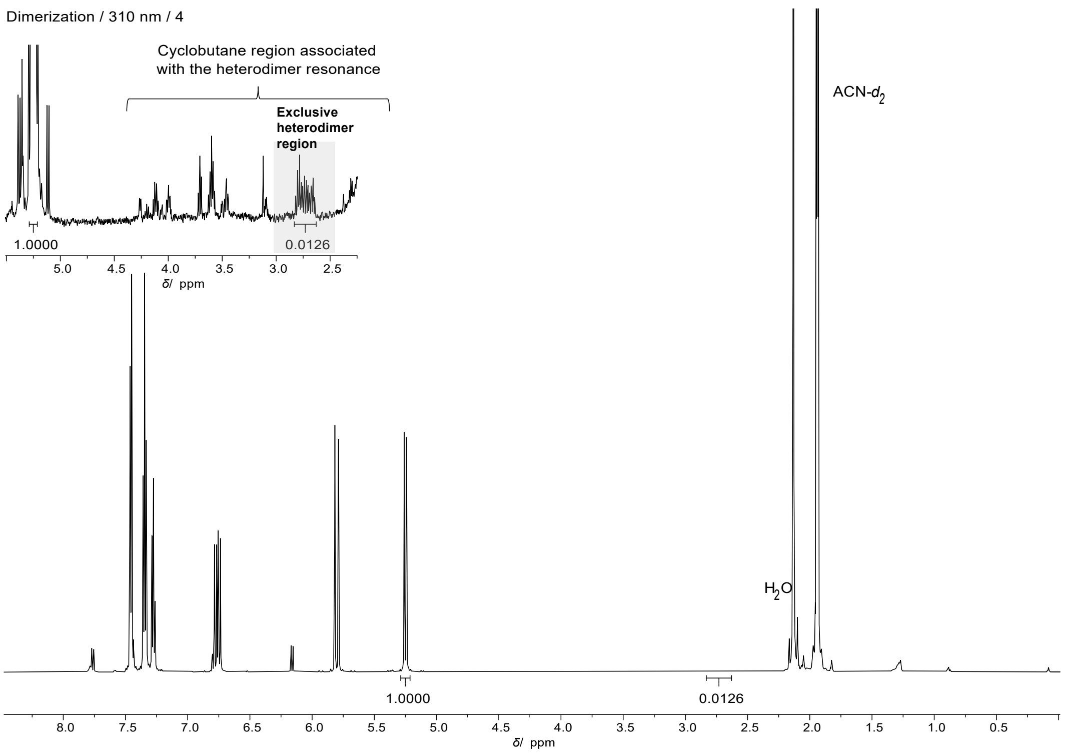

Figure S48 ^{1}H-NMR spectrum of 7-hydroxycoumarin with styrene in ACN-d_3 irradiated at 310 nm for 1 h, replicate 4. The zoomed insert visualizes the cyclobutane region and highlights the integrated area used to determine the heterodimer conversion.

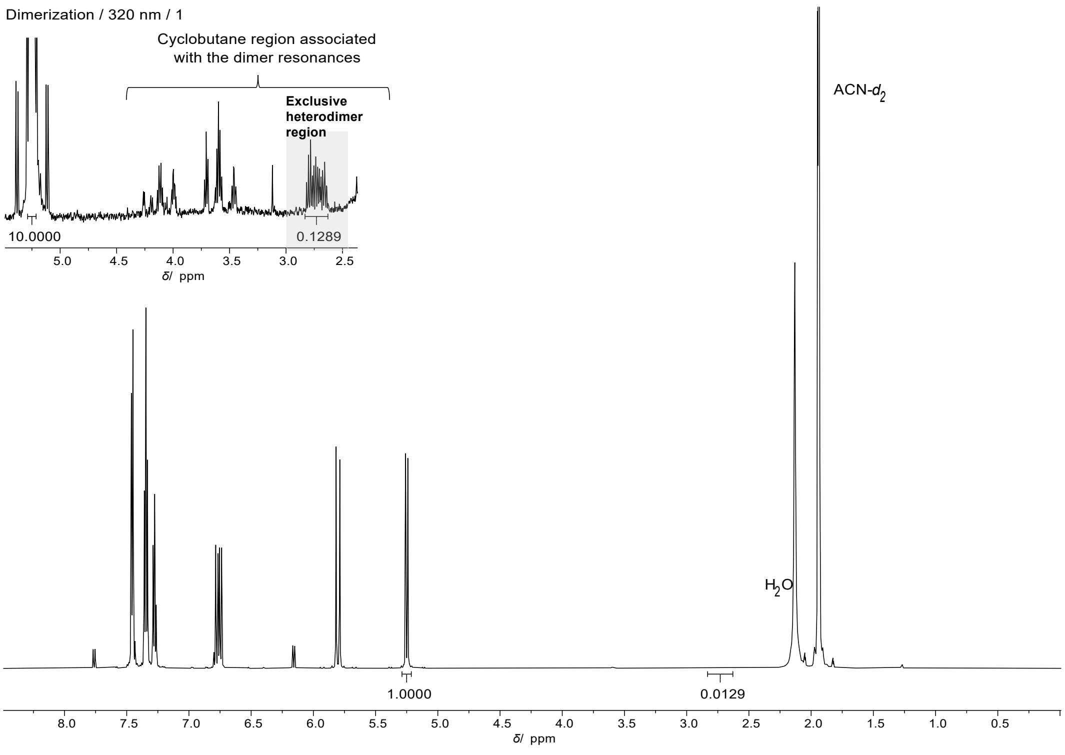

Figure S49 ^{1}H-NMR spectrum of 7-hydroxycoumarin with styrene in ACN-d_3 irradiated at 320 nm for 1 h, replicate 1. The zoomed insert visualizes the cyclobutane region and highlights the integrated area used to determine the heterodimer conversion.

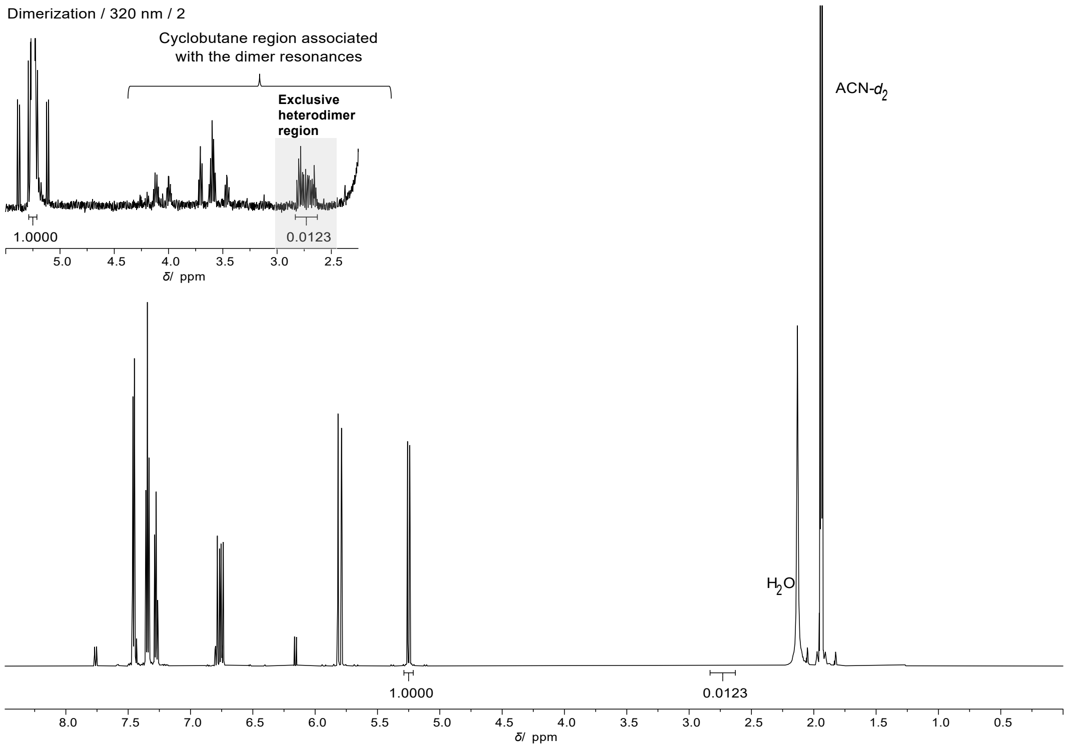

Figure S50 ^{1}H-NMR spectrum of 7-hydroxycoumarin with styrene in ACN-d_3 irradiated at 320 nm for 1 h, replicate 2. The zoomed insert visualizes the cyclobutane region and highlights the integrated area used to determine the heterodimer conversion.

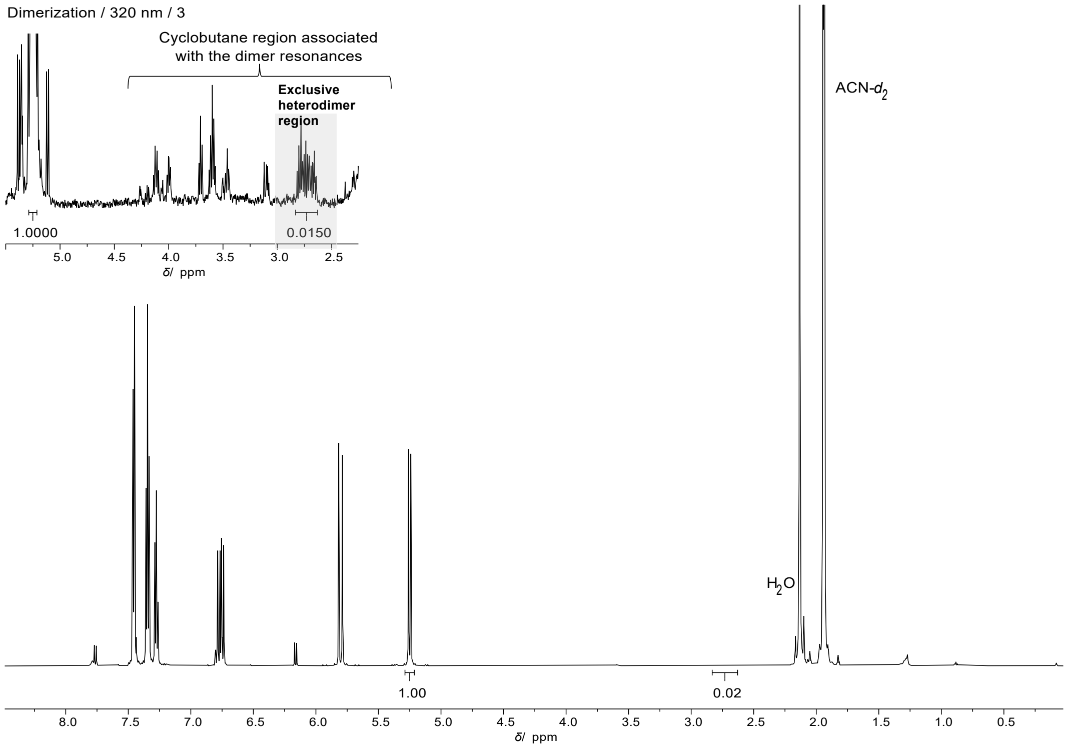

Figure S51 ^{1}H-NMR spectrum of 7-hydroxycoumarin with styrene in ACN-d_3 irradiated at 320 nm for 1 h, replicate 3. The zoomed insert visualizes the cyclobutane region and highlights the integrated area used to determine the heterodimer conversion.

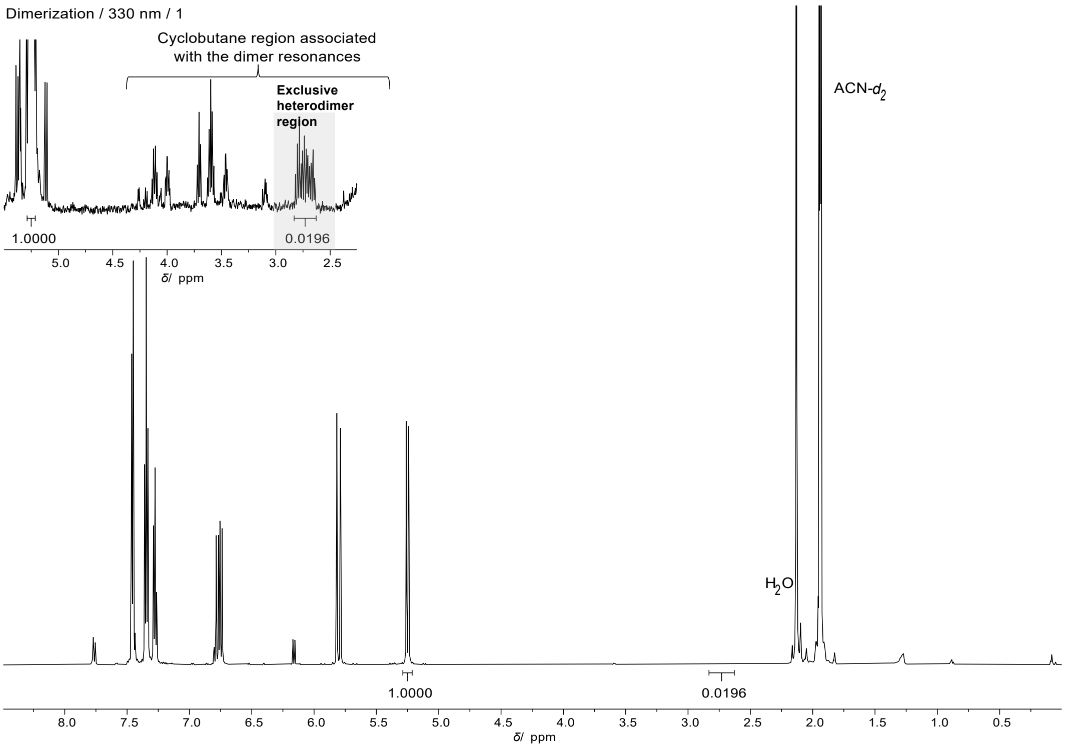

Figure S52 ^{1}H-NMR spectrum of 7-hydroxycoumarin with styrene in ACN-d_3 irradiated at 330 nm for 1 h, replicate 1. The zoomed insert visualizes the cyclobutane region and highlights the integrated area used to determine the heterodimer conversion.

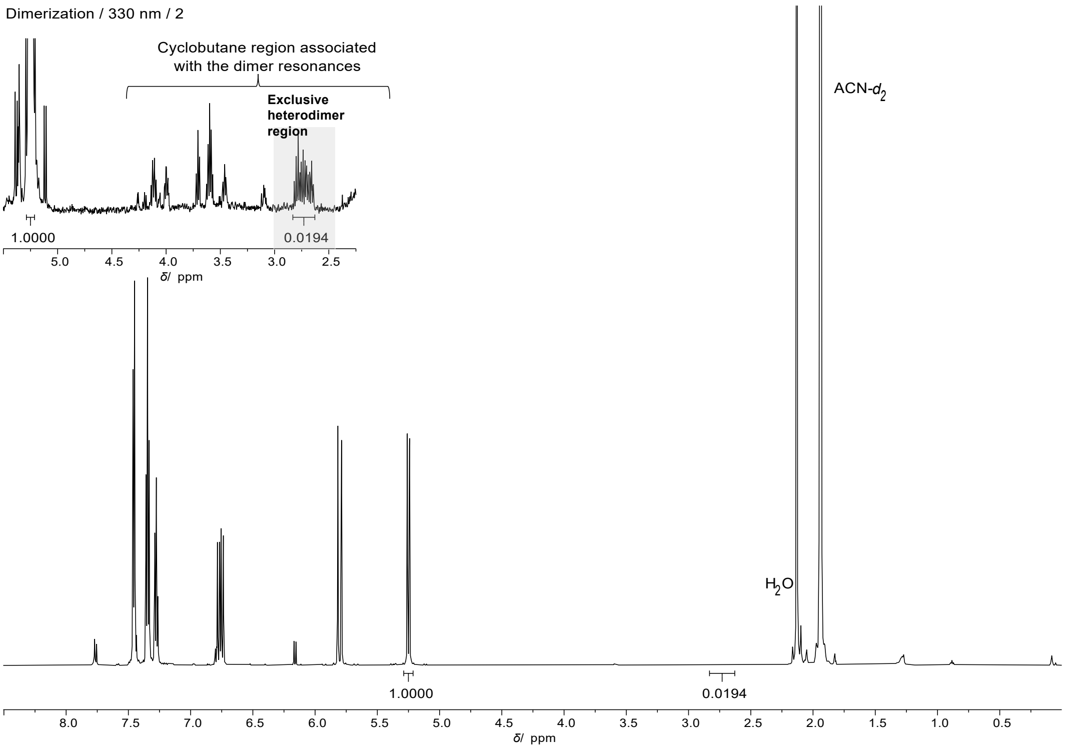

Figure S53 ^{1}H-NMR spectrum of 7-hydroxycoumarin with styrene in ACN-d_3 irradiated at 330 nm for 1 h, replicate 2. The zoomed insert visualizes the cyclobutane region and highlights the integrated area used to determine the heterodimer conversion.

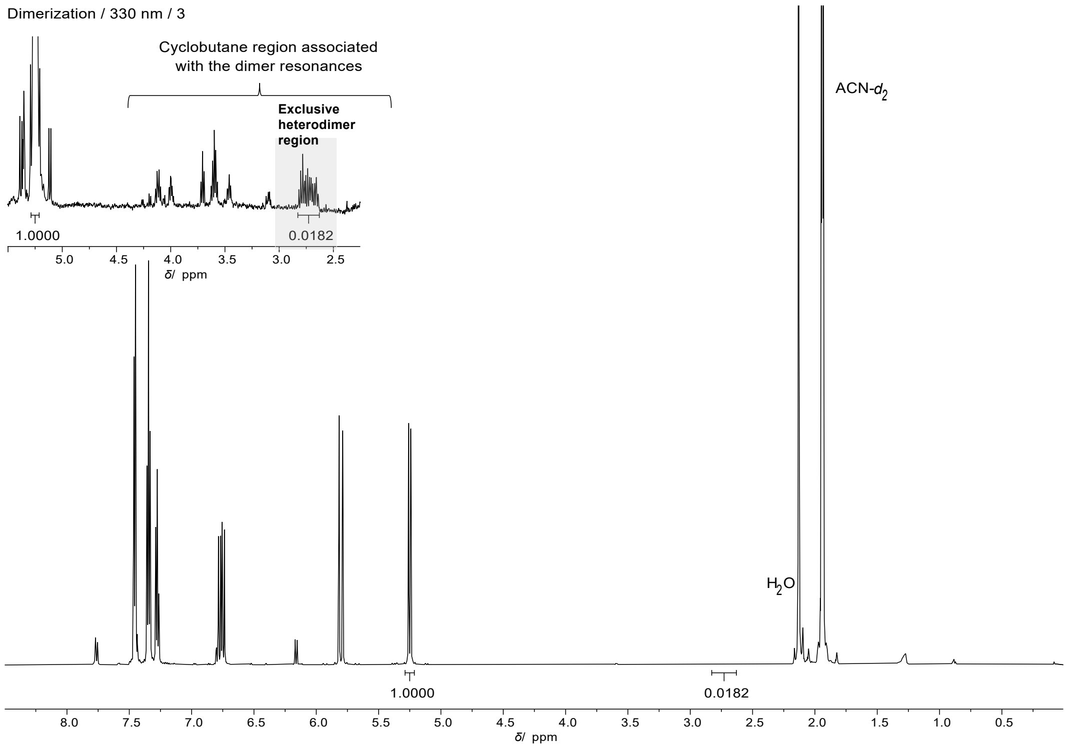

Figure S54 ^{1}H-NMR spectrum of 7-hydroxycoumarin with styrene in ACN-d_3 irradiated at 330 nm for 1 h, replicate 3. The zoomed insert visualizes the cyclobutane region and highlights the integrated area used to determine the heterodimer conversion.

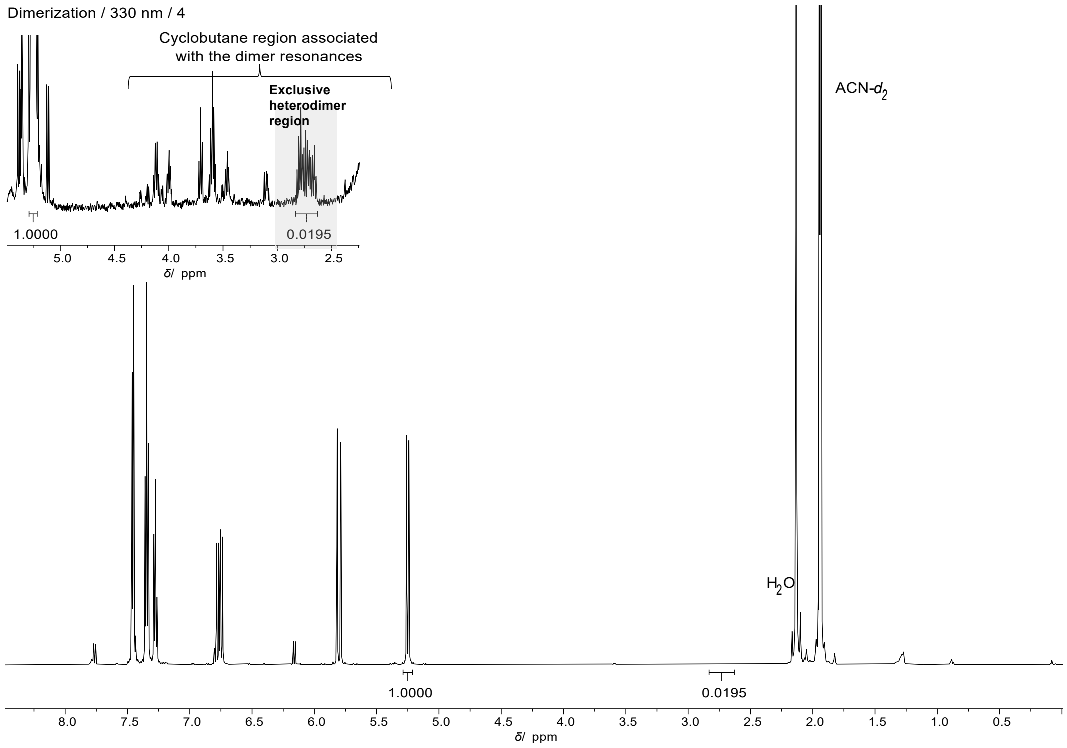

Figure S55 ^{1}H-NMR spectrum of 7-hydroxycoumarin with styrene in ACN-d_3 irradiated at 330 nm for 1 h, replicate 4. The zoomed insert visualizes the cyclobutane region and highlights the integrated area used to determine the heterodimer conversion.

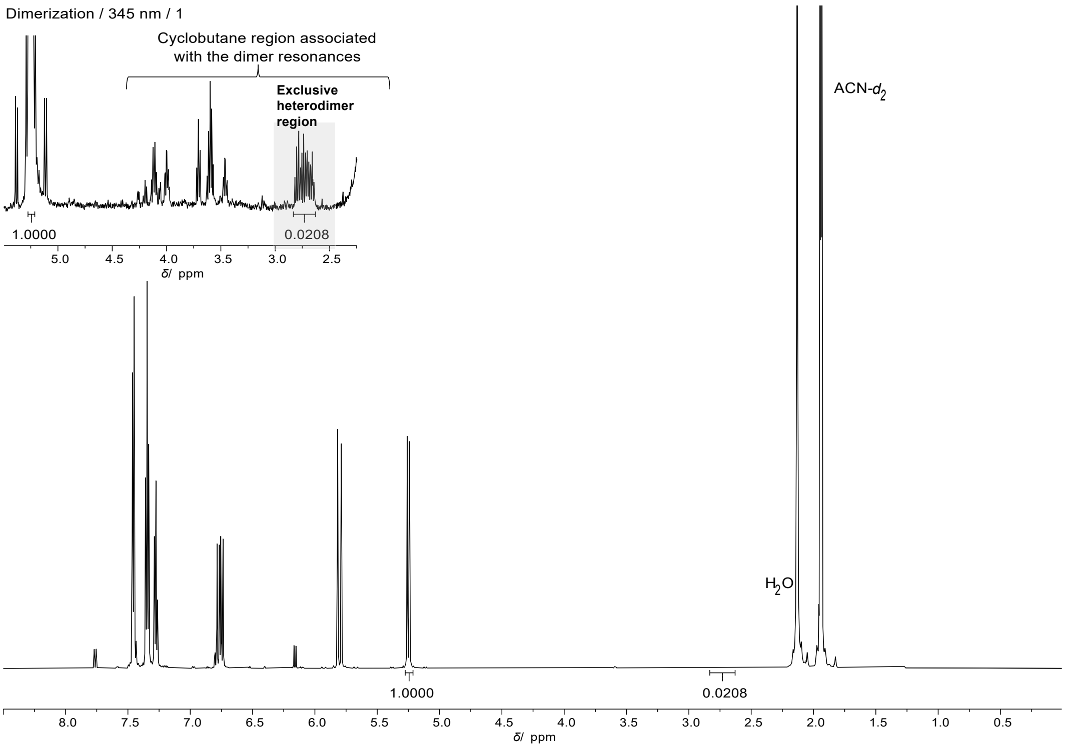

Figure S56 ^{1}H-NMR spectrum of 7-hydroxycoumarin with styrene in ACN-d_3 irradiated at 345 nm for 1 h, replicate 1. The zoomed insert visualizes the cyclobutane region and highlights the integrated area used to determine the heterodimer conversion.

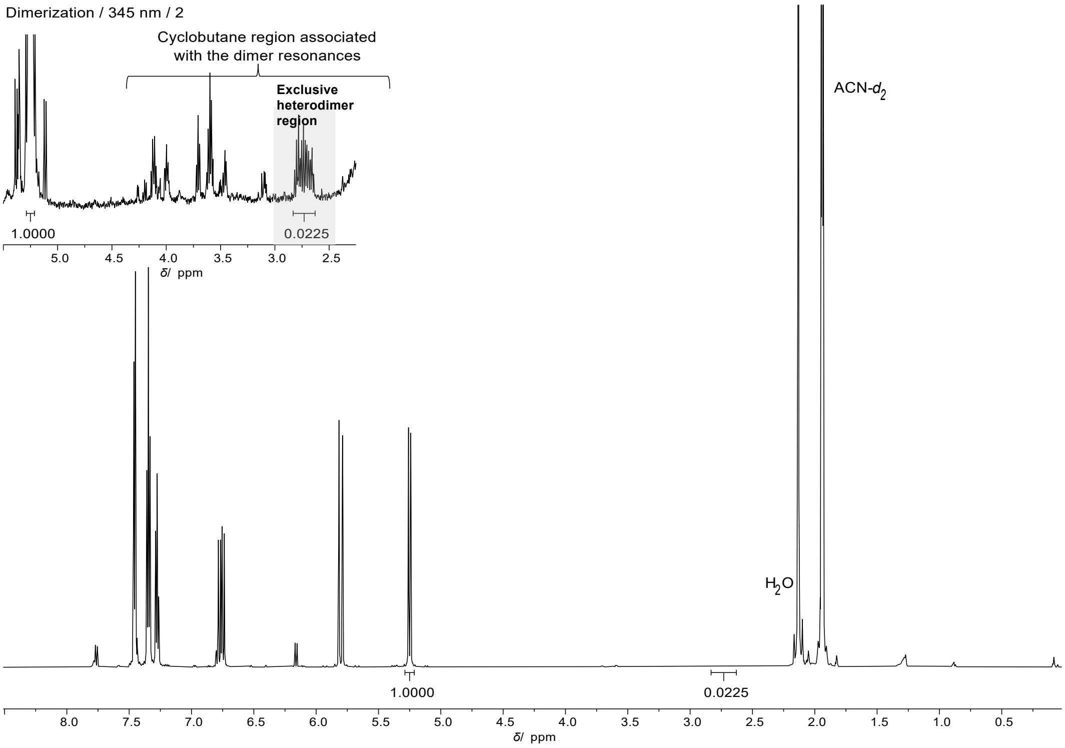

Figure S57 ^{1}H-NMR spectrum of 7-hydroxycoumarin with styrene in ACN-d_3 irradiated at 345 nm for 1 h, replicate 2. The zoomed insert visualizes the cyclobutane region and highlights the integrated area used to determine the heterodimer conversion.

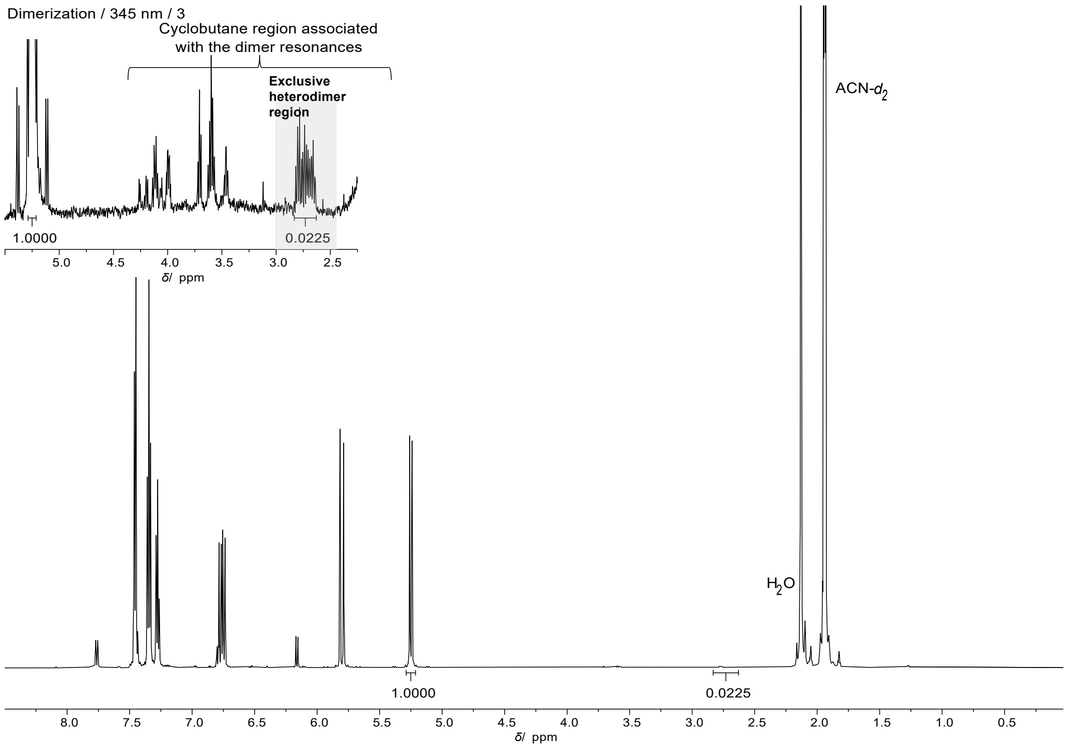

Figure S58 ^{1}H-NMR spectrum of 7-hydroxycoumarin with styrene in ACN-d_3 irradiated at 345 nm for 1 h, replicate 3. The zoomed insert visualizes the cyclobutane region and highlights the integrated area used to determine the heterodimer conversion.

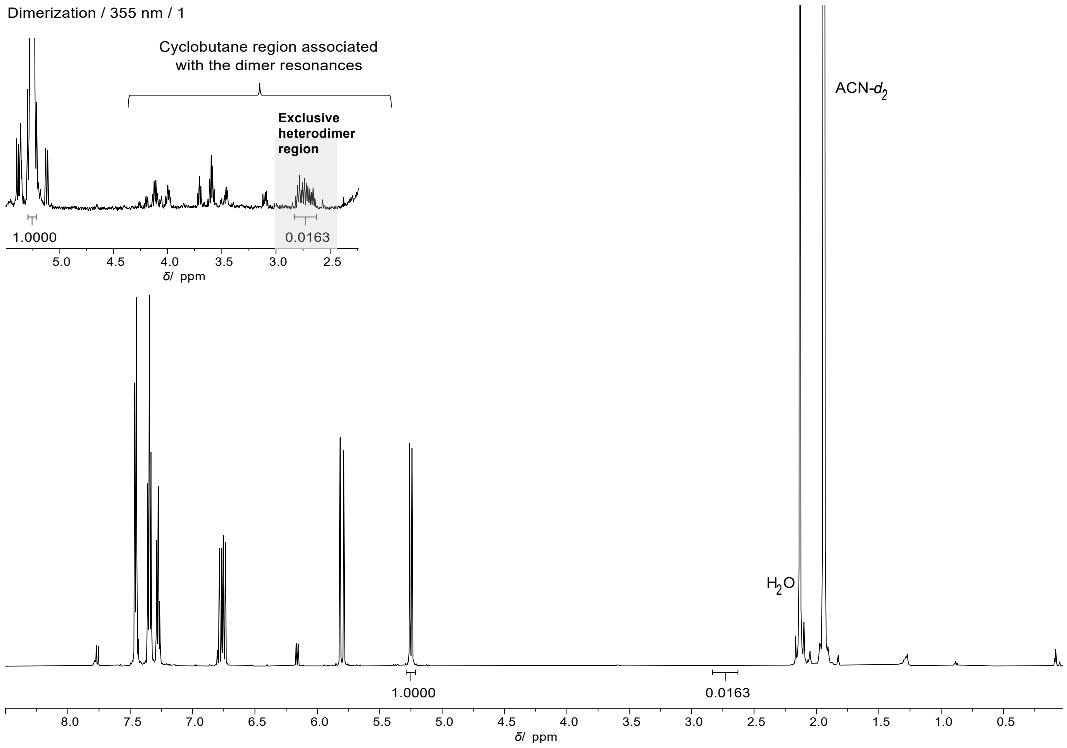

Figure S59 ^{1}H-NMR spectrum of 7-hydroxycoumarin with styrene in ACN-d_3 irradiated at 355 nm for 1 h, replicate 1. The zoomed insert visualizes the cyclobutane region and highlights the integrated area used to determine the heterodimer conversion.

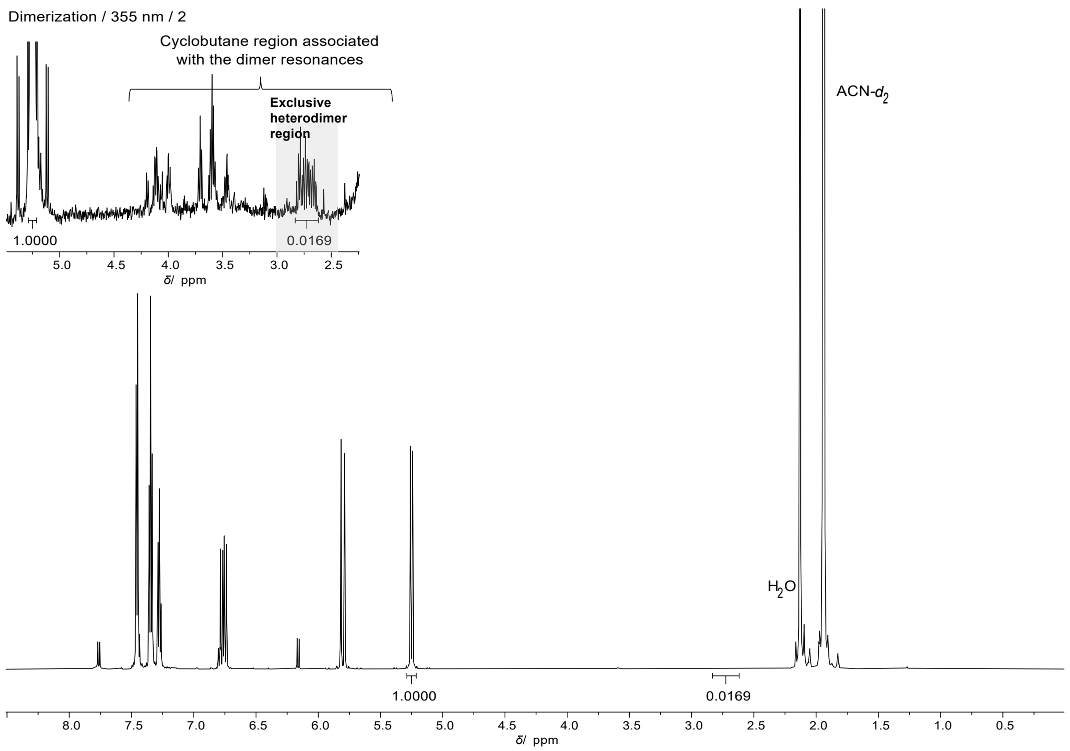

Figure S60 ^{1}H-NMR spectrum of 7-hydroxycoumarin with styrene in ACN-d_3 irradiated at 355 nm for 1 h, replicate 2. The zoomed insert visualizes the cyclobutane region and highlights the integrated area used to determine the heterodimer conversion.

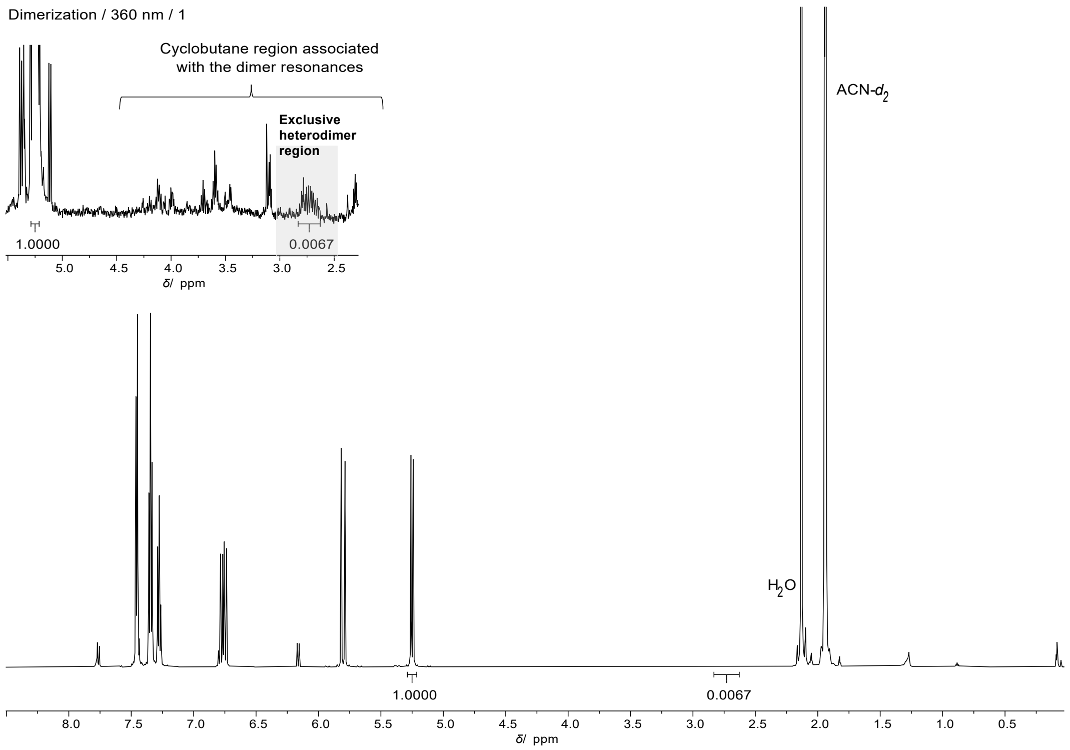

Figure S61 ^{1}H-NMR spectrum of 7-hydroxycoumarin with styrene in ACN-d_3 irradiated at 360 nm for 1 h, replicate 1. The zoomed insert visualizes the cyclobutane region and highlights the integrated area used to determine the heterodimer conversion.

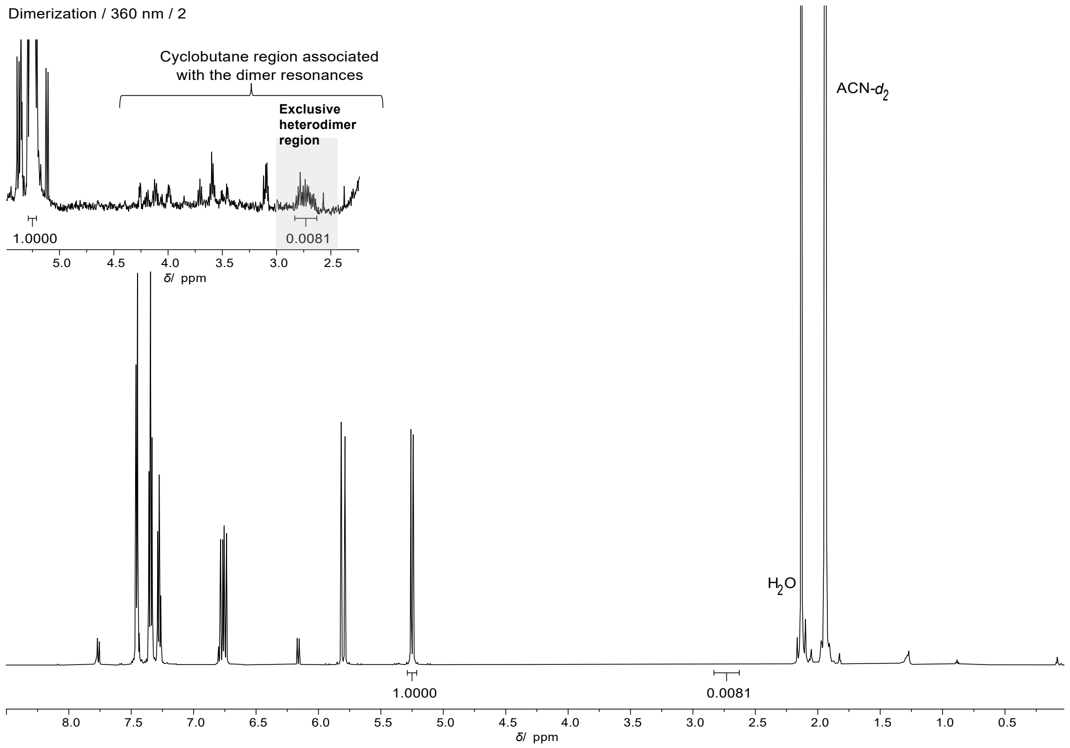

Figure S62 ^{1}H-NMR spectrum of 7-hydroxycoumarin with styrene in ACN-d_3 irradiated at 360 nm for 1 h, replicate 2. The zoomed insert visualizes the cyclobutane region and highlights the integrated area used to determine the heterodimer conversion.

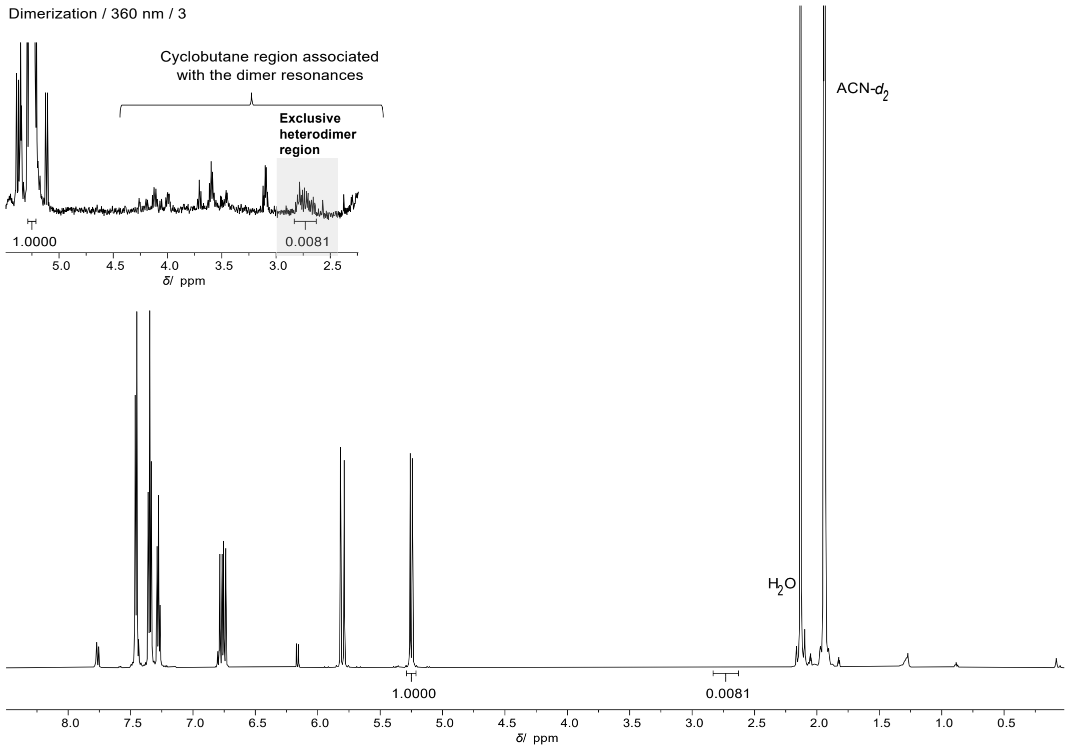

Figure S63 ^{1}H-NMR spectrum of 7-hydroxycoumarin with styrene in ACN-d_3 irradiated at 360 nm for 1 h, replicate 3. The zoomed insert visualizes the cyclobutane region and highlights the integrated area used to determine the heterodimer conversion.

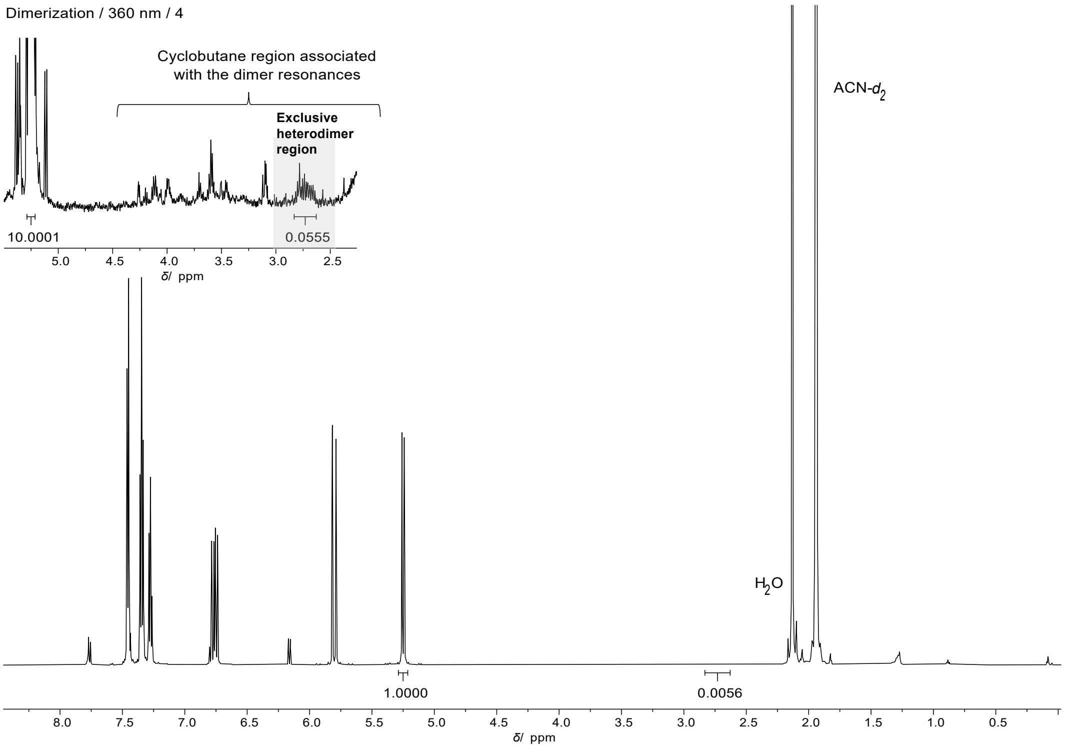

Figure S64 ^{1}H-NMR spectrum of 7-hydroxycoumarin with styrene in ACN-d_3 irradiated at 360 nm for 1 h, replicate 4. The zoomed insert visualizes the cyclobutane region and highlights the integrated area used to determine the heterodimer conversion.

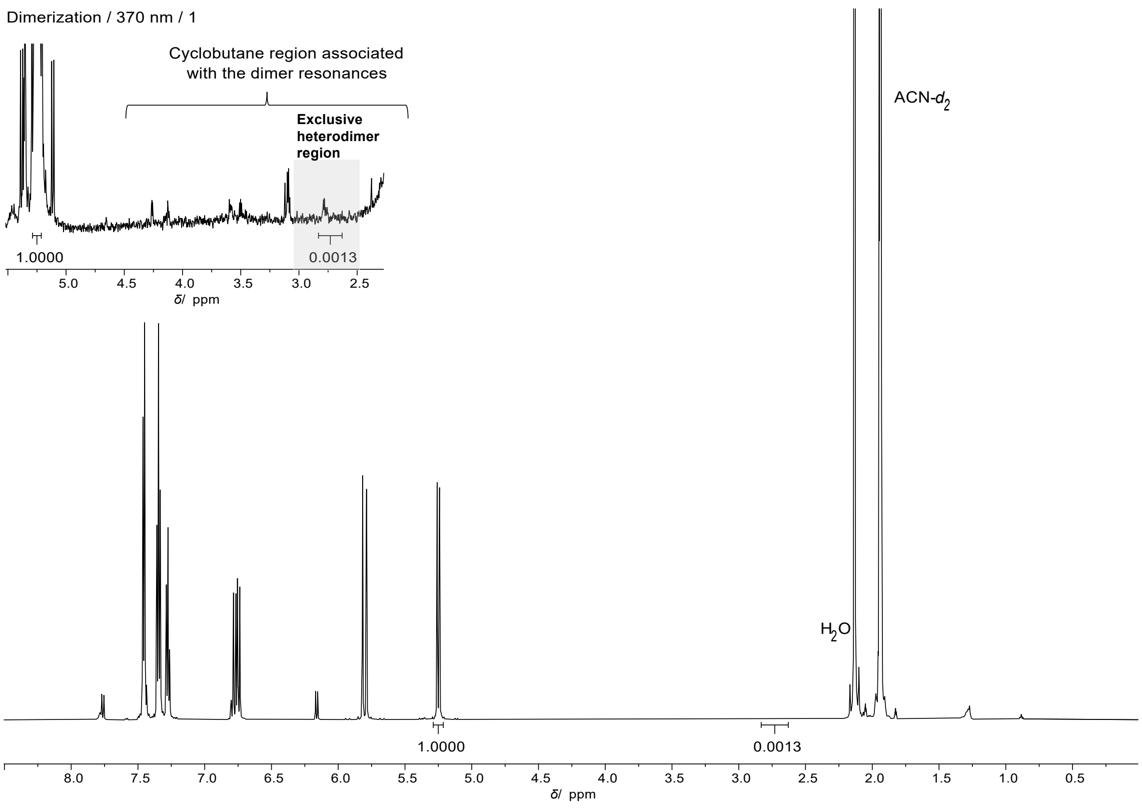

Figure S65 ^{1}H-NMR spectrum of 7-hydroxycoumarin with styrene in ACN-d_3 irradiated at 370 nm for 1 h, replicate 1. The zoomed insert visualizes the cyclobutane region and highlights the integrated area used to determine the heterodimer conversion.

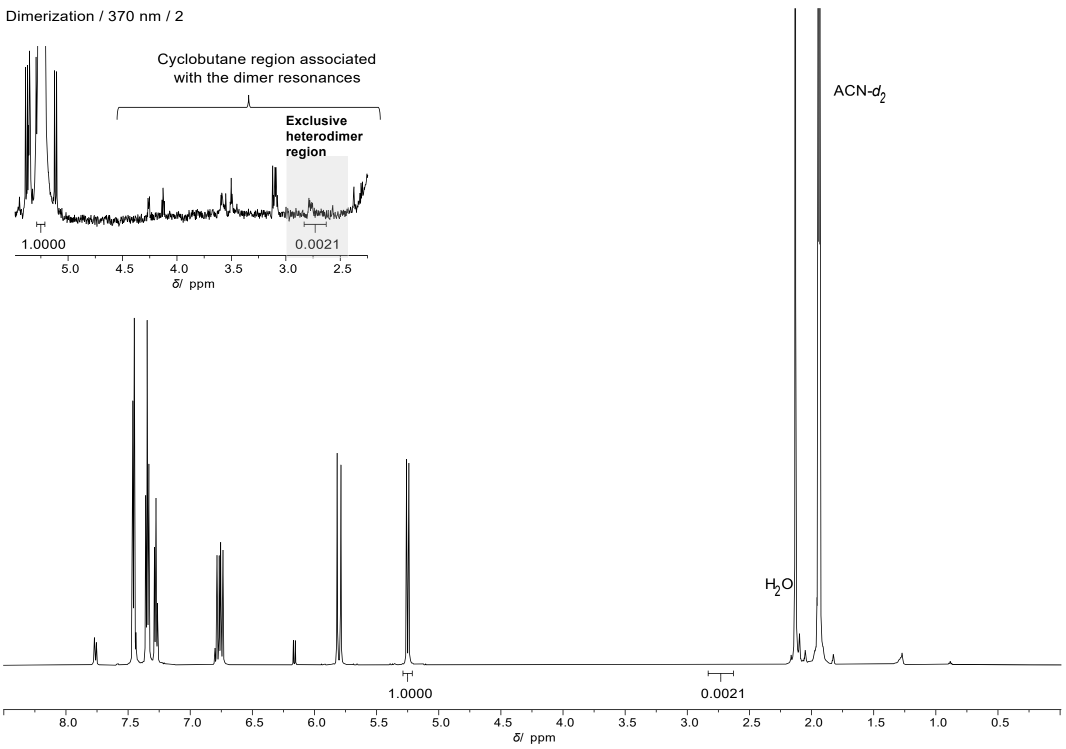

Figure S66 ^{1}H-NMR spectrum of 7-hydroxycoumarin with styrene in ACN-d_3 irradiated at 370 nm for 1 h, replicate 2. The zoomed insert visualizes the cyclobutane region and highlights the integrated area used to determine the heterodimer conversion.

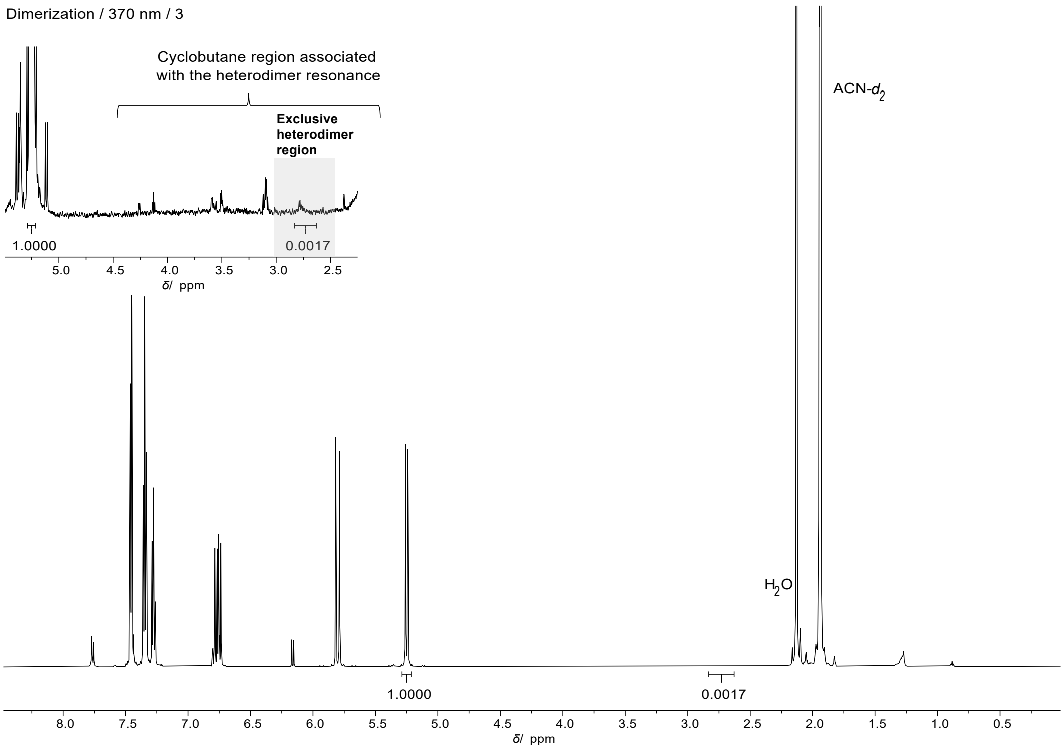

Figure S67 ^{1}H-NMR spectrum of 7-hydroxycoumarin with styrene in ACN-d_3 irradiated at 370 nm for 1 h, replicate 3. The zoomed insert visualizes the cyclobutane region and highlights the integrated area used to determine the heterodimer conversion.

4.2 Contact Angle Data for the Resin Samples

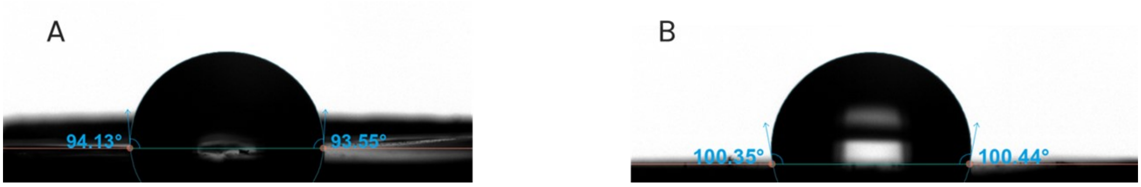

Figure S68 Contact angle measurements on the cured resin. **A** Freshly cured resin surface. **B** After the StyPFA treatment on the resin surface.

4.3 FT-IR Spectra

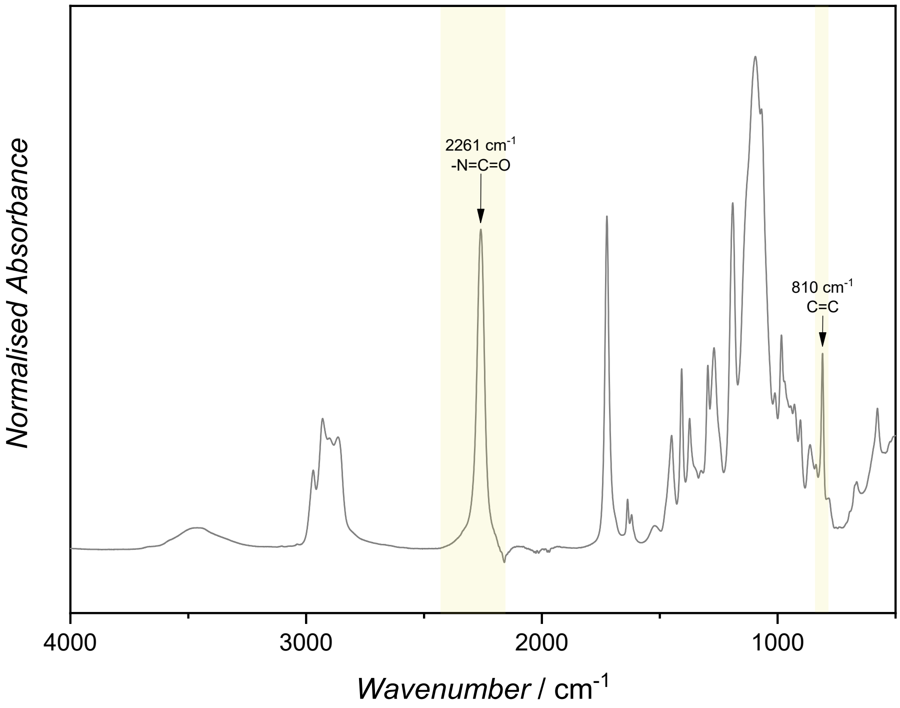

Figure S69 FT-IR spectrum of the photoresin in the range of 500 to 4000 cm^{-1}. Characteristic absorption bands are highlighted to illustrate functional groups associated with the isocyanate peak (2261 cm^{-1}) and the double bond of the acrylate (810 cm^{-1}).

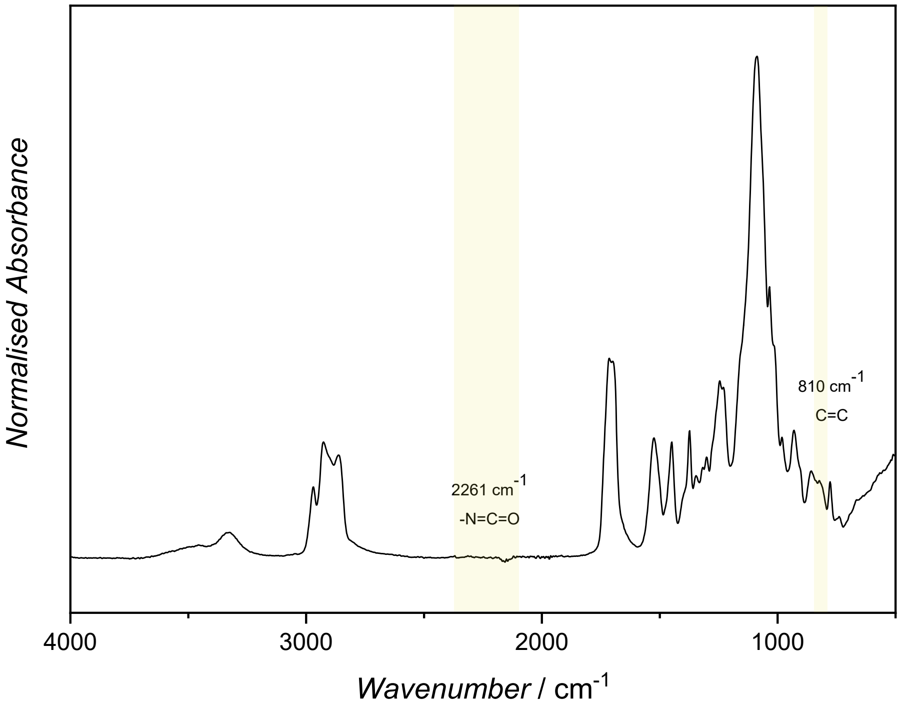

Figure S70 FT-IR spectrum of cured photoresin in the range of 500 to 4000 cm^{-1}. Characteristic absorption bands are highlighted to illustrate functional groups associated with the isocyanate peak (2261 cm^{-1}) and the double bond of the acrylate (810 cm^{-1}). Differences between the spectra indicate variations in chemical structure and network formation.

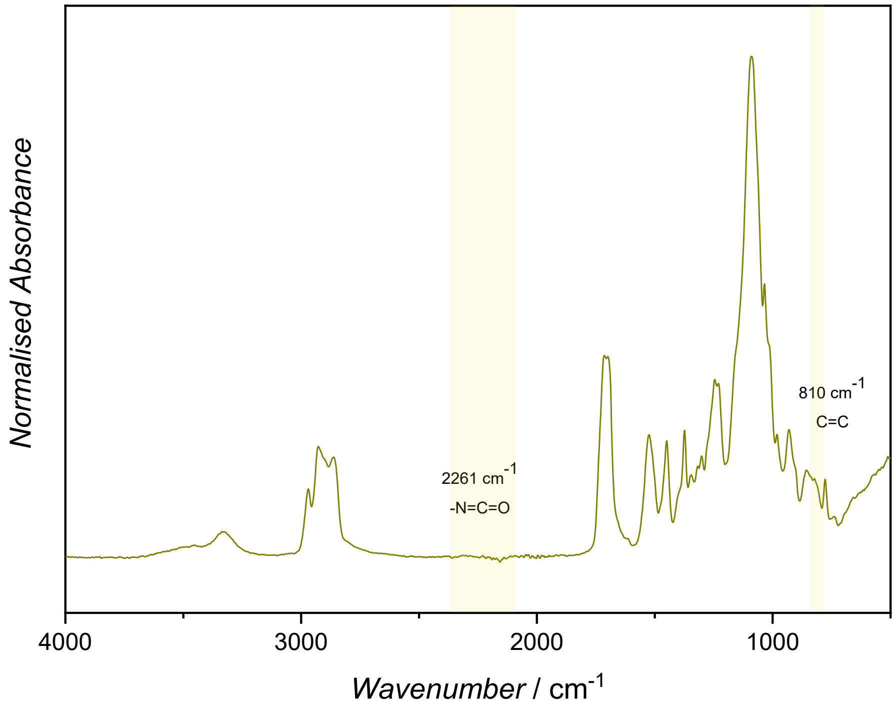

Figure S71 FT-IR spectrum of cured photoresin with coumarin in the range 500 to 4000 cm^{-1}. Characteristic absorption bands are highlighted to illustrate functional groups associated with the isocyanate peak (2261 cm^{-1}) and the double bond of the acrylate (810 cm^{-1}). Differences between the spectra indicate variations in chemical structure and network formation.

4.4 Dynamic Mechanical Analysis of the Resin Samples

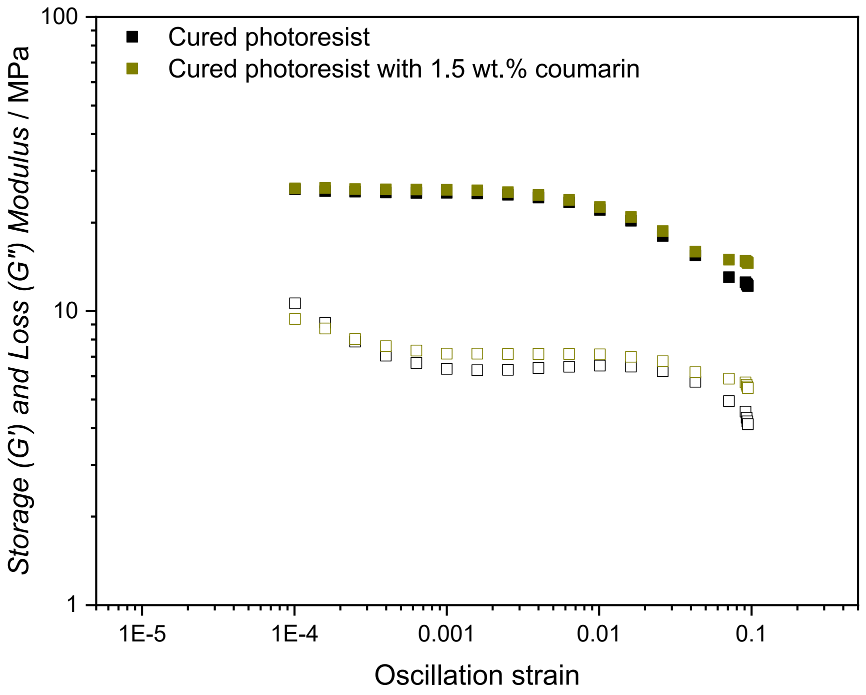

Figure S72 Amplitude sweep measured by DMA for cured photoresin and cured photoresin with coumarin at ambient temperature and 1 Hz. The storage modulus (*G*′) and loss modulus (*G*″) are plotted as a function of strain amplitude, illustrating the linear viscoelastic region (LVR) and the onset of non-linear behaviour.

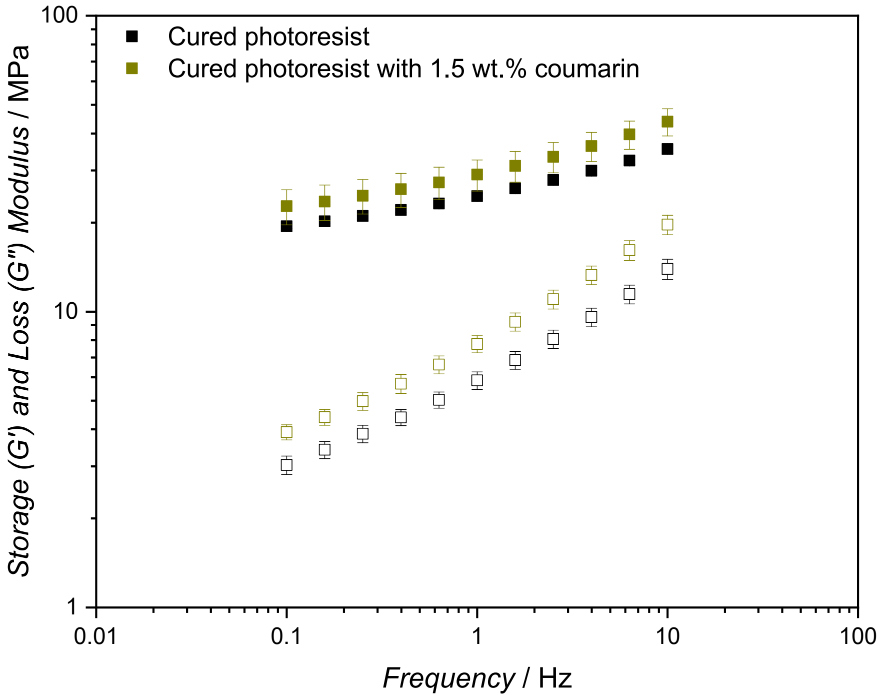

Figure S73 Frequency sweep of cured photoresin and cured photoresin with coumarin at ambient temperature in the linear viscoelastic region (oscillation strain of 0.0016). The storage modulus (*G*′) and loss modulus (*G*″) are plotted as a function of frequency (10–0.1 Hz), illustrating differences in viscoelastic behaviour and network dynamics between the two formulations.

5 References

(1) Irshadeen, I. M.; Walden, S. L.; Wegener, M.; Truong, V. X.; Frisch, H.; Blinco, J. P.; Barner-Kowollik, C. Action Plots in Action: In-Depth Insights into Photochemical Reactivity. *Journal of the American Chemical Society* **2021**, *143* (50), 21113-21126. DOI: 10.1021/jacs.1c09419.

(2) Carroll, J. A.; Pashley-Johnson, F.; Klein, M.; Stephan, T.; Pandey, A. K.; Walter, M.; Unterreiner, A. N.; Barner-Kowollik, C. Microenvironments as an Explanation for the Mismatch between Photochemical Absorptivity and Reactivity. *J Am Chem Soc* **2025**, *147* (30), 26643-26651. DOI: 10.1021/jacs.5c06961 From NLM PubMed-not-MEDLINE.

(3) Do, P. T.; Richardson, B.; Brydon, S. C.; Fulloon, T. M.; Blanksby, S. J.; Frisch, H.; Poad, B. L. J. Mass Spectrometry Directed Structural Elucidation of Isomeric [2 + 2] Photocycloadducts. *Analytical Chemistry* **2025**, *97* (14), 7711-7718. DOI: 10.1021/acs.analchem.4c05228.

(4) Marschner, D. E.; Frisch, H.; Offenloch, J. T.; Tuten, B. T.; Becer, C. R.; Walther, A.; Goldmann, A. S.; Tzvetkova, P.; Barner-Kowollik, C. Visible Light [2 + 2] Cycloadditions for Reversible Polymer Ligation. *Macromolecules* **2018**, *51* (10), 3802-3807. DOI: 10.1021/acs.macromol.8b00613.

(5) He, X.; Tian, Y.; O'Neill, R. T.; Xu, Y.; Lin, Y.; Weng, W.; Boulatov, R. Coumarin Dimer Is an Effective Photomechanochemical AND Gate for Small-Molecule Release. *Journal of the American Chemical Society* **2023**, *145* (42), 23214-23226. DOI: 10.1021/jacs.3c07883.
